# miR-182 and miR-10a Are Key Regulators of Treg Specialisation and Stability during *Schistosome* and *Leishmania*-associated Inflammation

**DOI:** 10.1371/journal.ppat.1003451

**Published:** 2013-06-27

**Authors:** Samir Kelada, Praveen Sethupathy, Isobel S. Okoye, Eleni Kistasis, Stephanie Czieso, Sandra D. White, David Chou, Craig Martens, Stacy M. Ricklefs, Kimmo Virtaneva, Dan E. Sturdevant, Stephen F. Porcella, Yasmine Belkaid, Thomas A. Wynn, Mark S. Wilson

**Affiliations:** 1 Department of Genetics, University of North Carolina at Chapel Hill, Chapel Hill, North Carolina, United States of America; 2 Division of Molecular Immunology, MRC, National Institute for Medical Research, London, United Kingdom; 3 Laboratory of Parasitic Diseases, National Institutes of Allergy and Infectious Disease, Bethesda, Maryland, United States of America; 4 Research Technologies Section, Rocky Mountain Laboratories, Hamilton, Montana, United States of America; University of Cambridge, United Kingdom

## Abstract

A diverse suite of effector immune responses provide protection against various pathogens. However, the array of effector responses must be immunologically regulated to limit pathogen- and immune-associated damage. CD4^+^Foxp3^+^ regulatory T cells (Treg) calibrate immune responses; however, how Treg cells adapt to control different effector responses is unclear. To investigate the molecular mechanism of Treg diversity we used whole genome expression profiling and next generation small RNA sequencing of Treg cells isolated from type-1 or type-2 inflamed tissue following *Leishmania major* or *Schistosoma mansoni* infection, respectively. *In-silico* analyses identified two miRNA “regulatory hubs” miR-10a and miR-182 as critical miRNAs in Th1- or Th2-associated Treg cells, respectively. Functionally and mechanistically, in-vitro and in-vivo systems identified that an IL-12/IFNγ axis regulated miR-10a and its putative transcription factor, Creb. Importantly, reduced miR-10a in Th1-associated Treg cells was critical for Treg function and controlled a suite of genes preventing IFNγ production. In contrast, IL-4 regulated miR-182 and cMaf in Th2-associed Treg cells, which mitigated IL-2 secretion, in part through repression of IL2-promoting genes. Together, this study indicates that CD4^+^Foxp3^+^ cells can be shaped by local environmental factors, which orchestrate distinct miRNA pathways preserving Treg stability and suppressor function.

## Author Summary

The diversity of pathogens that the immune system encounters are controlled by a diverse suite of immunological effector responses. Preserving a well-controlled protective immune response is essential. Too vigorous an effector response can be as damaging as too little. Regulatory T cells (Treg) calibrate immune responses; however, how Treg cells adapt to control the diverse suite of effector responses is unclear. In this study we investigated the molecular identity of regulatory T cells that control distinct effector immune responses against two discrete pathogens, an intracellular parasitic protozoa, *Leishmania major*, and an extracellular helminth parasite, *Schitsosoma mansoni*. The two Treg populations studied were phenotypically and functionally different. We identified molecular pathways that influence this diversity and more specifically, we identified that two miRNAs (miR-182 and miR-10a) act as “regulatory hubs” critically controlling distinct properties within each Treg population. This is the first study identifying the upstream molecular pathways controlling Treg cell specialization and provides a new platform of Treg cell manipulation to fine-tune their function.

## Introduction

Regulatory T (Treg) cells [1] employ an arsenal of non-overlapping mechanisms to maintain immunological homeostasis at environmental interfaces [2] and internal organs [3], preventing the development of hyper-inflammatory conditions [4], [5]. The suppressive functions of Treg cells are crucial, without which fatal lympho- and myelo-proliferative autoimmune syndromes develop [6]. Restoring immunological homeostasis with regulatory T cell-based therapy may remedy some hyper-inflammatory conditions [7]. Regulatory T cells also restrict *de novo* responses to foreign antigens, limiting immunopathologies but sometimes at the cost of preventing natural, or vaccine-mediated, immunity [8]. In this context, temporarily disarming Treg functions may increase the efficacy of vaccines and immunity to infection. Elemental to any Treg-based therapeutic strategy is manipulating the appropriate Treg cells. Expression of the transcription factor forkhead box P3 (Foxp3) in αβ^+^CD4^+^ lymphocytes activates and represses a suite of target genes [9] essential for Treg development and function. For this reason, Foxp3 expression is commonly used as a marker of Treg cells and is often used to compare Treg cells from a variety of different diseases. It has recently emerged that Foxp3^+^ Treg cells are heterogeneous and may be as diverse as the types of immune responses they regulate [10]–[14]. Foxp3^+^ Treg cells therefore represent a population of loosely related lymphocytes, still requiring greater molecular characterization.

Foxp3^+^ cell development and function is intricately controlled transcriptionally by epigenetic modifications influencing gene accessibility [15] and post-transcriptionally by microRNAs (miRNAs) [16]. miRNAs have emerged as key regulators of innate and adaptive immune responses [17] and confer robustness and adaptability to cells in response to environmental fluctuation [18]. Disrupting canonical miRNA biogenesis by ablating the miRNA processing enzymes *Dicer* or *Drosha* in T cells [19]–[21] dysregulated T cell proliferation, differentiation, survival and cytokine production leading to a reduction in Foxp3^+^ cells and subsequent lethal inflammation [19]. Deletion of the entire miRNA repertoire specifically within Foxp3^+^ cells phenocopied *Foxp3^−/−^* mice with a loss of Treg function and the development of fatal autoimmunity [22], [23]. These studies highlight the crucial role of miRNA-mediated gene regulation in Treg biology. However, which miRNAs are required for different Tregs and Treg-associated functions is poorly understood.

Several miRNAs (miR-21, miR-31, miR-24 and miR-210) [24], [25] directly target Foxp3 in human T cells, regulating Foxp3 expression and Treg development. Additionally, Foxp3 activates miRNA-mediated mechanisms [25] to repress effector pathways, including suppression of SOCS1 via induction of miR-155 [26]. These studies indicate an intricate functional relationship between Foxp3 and miRNAs. Furthermore, Lu and colleagues [27] recently identified a role for miR-146a in regulating the expression of Stat1, which is required for Treg-mediated control of Th1 responses. While such individual miRNA:target interactions are of interest, a single miRNA can target hundreds of mRNAs [28], simultaneously regulating multiple pathways.

We hypothesized that widespread miRNA-mediated regulation contributes to Foxp3^+^ cell diversity. To test this, we isolated Foxp3^+^ cells from mice chronically infected with *Schistosoma mansoni*, a parasitic helminth that invokes a polarised Th2 response, or *Leishmania major*, a parasitic protozoa controlled by Th1-mediated immunity. Microarray analysis revealed dramatically different gene expression profiles, confirming the heterogeneity of Foxp3^+^ cells. To investigate which miRNAs contribute to the observed gene expression differences, we first deep sequenced the small RNAome from these two Foxp3^+^ populations and identified several miRNAs that were significantly differentially expressed, relative to Treg cells taken from naïve mice. These miRNAs were analyzed further using our recently published *in silico* method [29] for predicting candidate ‘regulatory hubs’. miR-10a was identified as the strongest such regulatory hub in *L. major* Foxp3^+^ cells, whereas miR-182 was the most critical in *S. mansoni* Foxp3^+^ cells. Gain and loss of function experiments in vitro and in vivo using primary Foxp3^+^ cells and Foxp3^+^ cells isolated from Th1 or Th2 inflamed tissue confirmed many of the predicted targets and functions for miR-10a and miR-182. We further demonstrated that IL-4 up-regulates miR-182, potentially through the transcription factor cMaf, which is also up-regulated by IL-4. miR-182 critically restricted IL-2 production, possibly by its control of *Bach2* [30] and *Cd2ap* [31]. We also showed that IL-12/IFNγ represses both miR-10a and its candidate upstream transcription factor Creb. Reduced miR-10a correlated with an increase in miR-10a target genes, *Nr4a3* and *Fbxo30*, which have previously been shown to control IFNγ. Collectively, this study supports the concept of heterogeneity, or plasticity, within the Foxp3^+^ pool and identifies candidate ‘regulatory hub’ miRNAs, miR-10a and miR-182, which control IFNγ and IL-2 through essential gene programs.

## Results

### Foxp3^+^ Regulatory T cells employ distinct gene programs during chronic Th2-associated (*S. mansoni*) or Th1-associated (*L. major*) infection

**Figure 1:**
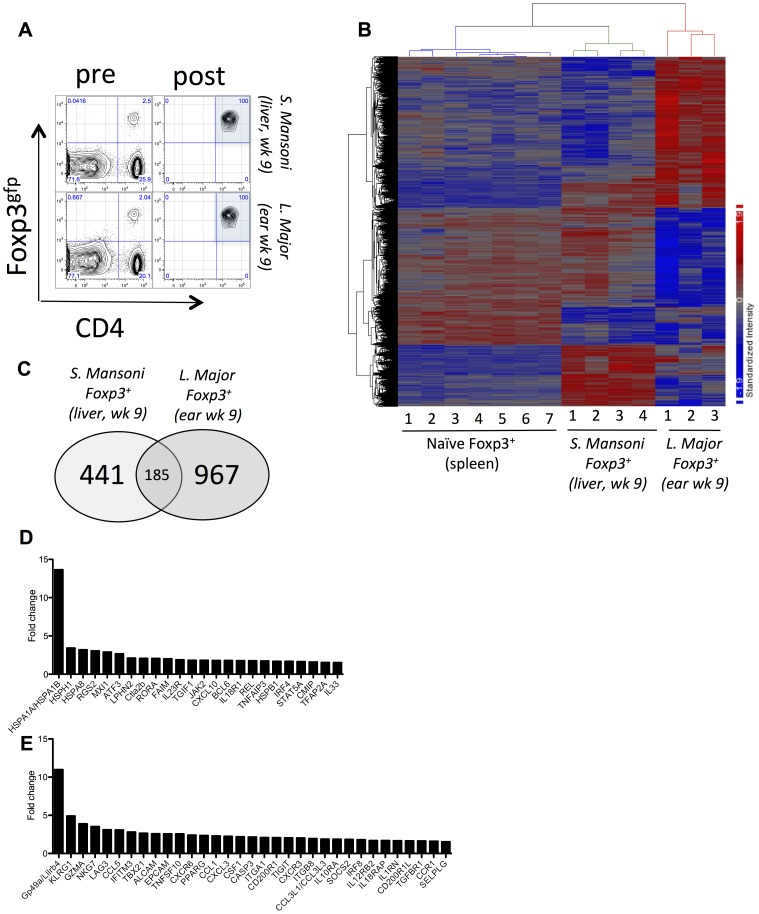
Differential gene expression in CD4^+^Foxp3^+^ cells isolated from chronic *S. Mansoni* or chronic *L. Major* infected tissue. (A) Isolation and FACS sorting of CD4^+^Foxp3^+^ cells from the liver of *S. Mansoni* or ear of *L. Major infected* mice. (B) Heat map of differential gene expression showing biological replicates *(naïve Treg = 7, S. m. Treg = 4, L. m. Treg = 3 biological replicates)* for the isolated Foxp3^+^ populations. 3944 array probes were differentially expressed at a false discovery rate (FDR) less than 0.05. The list of 3944 array probes is provided in Table S1. (C) Number of common (overlap) and unique genes that were differentially regulated between the Sm-Foxp3^+^ cells and Lm Foxp3^+^ cells, relative to ‘Naïve’ Foxp3+ cells (FDR<0.1 and fold change >1.5). (D) Immunity-associated genes up-regulated in Lm Foxp3^+^ cells. (E) Immunity-associated genes up-regulated in and Sm Foxp3^+^ cells.

Following infection with *Schistosoma mansoni* or *Leishmania major*, robust Th2 or Th1- responses develop [32], [33], accompanied by the recruitment of Foxp3^+^ Treg cells [34]–[39]. Genome-wide analysis of isolated Foxp3^+^ cells recruited to the liver of *S. mansoni* (*S. mansoni* Foxp3^+^) or ear of *L. major* (*L. major* Foxp3^+^) infected mice (**Fig. 1A**) identified distinct gene expression profiles, relative to Foxp3^+^ cells isolated from the spleen of uninfected mice (**Fig. 1B**). Of the differentially expressed genes, 185 (11.6%) were common between *L. major* and *S. mansoni*, whereas 441 (27.7%; *S. m*.) and 967 (60.7%; *L. m.*) were specific to each population (**Figure 1C**), indicating that, with respect to gene expression, these Foxp3^+^ populations were substantially different from one another. The vast majority of the 185 common genes (**Table S1**) were similarly regulated in each Treg population (**Figure S1**). Relative to Foxp3^+^ cells from the spleen of uninfected mice, *L. major* Foxp3^+^ cells upregulated several heat shock proteins (*Hsph1, Hspa8 and Hspa1a*), cytokine and chemokine-associated genes (*Il23r, Il33, IL18R1, Tgif1, Cxcl10, Rgs2, Lph2, Tnfaip3*) and a range of transcriptional regulators (*Bcl6, Mxi1, Atf3, Ror-α, Rel, Irf4, Stat5a, Tfap2a*) (**Figure 1 D**). *S. mansoni*-derived Foxp3^+^ cells, in contrast, upregulated genes associated with inhibition and killing (*Gp49a, Klrg1, Gzma, Nkg7, Lag3, Tigit Cd200r1 and Cd200r1l, Trail (Tnfsf10)*), integrins and adhesion molecules (*Alcam, Epcam, Itga1, Itgb8, Selplg (P-Selectin)*), cytokines and chemokines (*Csf1, Il10r1, Il12rb2, Il18rap, Il1rn, Tgfβr1, Socs2, Ccl5, Ccl1, Ccl3l, Cxcl3, Cxcr6, Cxcr3, Ccr1*), and different transcriptional regulators (*Tbx21*, *Pparγ* and *Irf8*) (**Figure 1E**), many of which were also observed in a previous report [36].

### Deep sequencing of small RNA species reveals distinct miRNA profiles between Foxp3^+^ cells isolated from chronic helminth (*S. m.*) or protozoan (*L. m.*) infection

**Figure 2:**
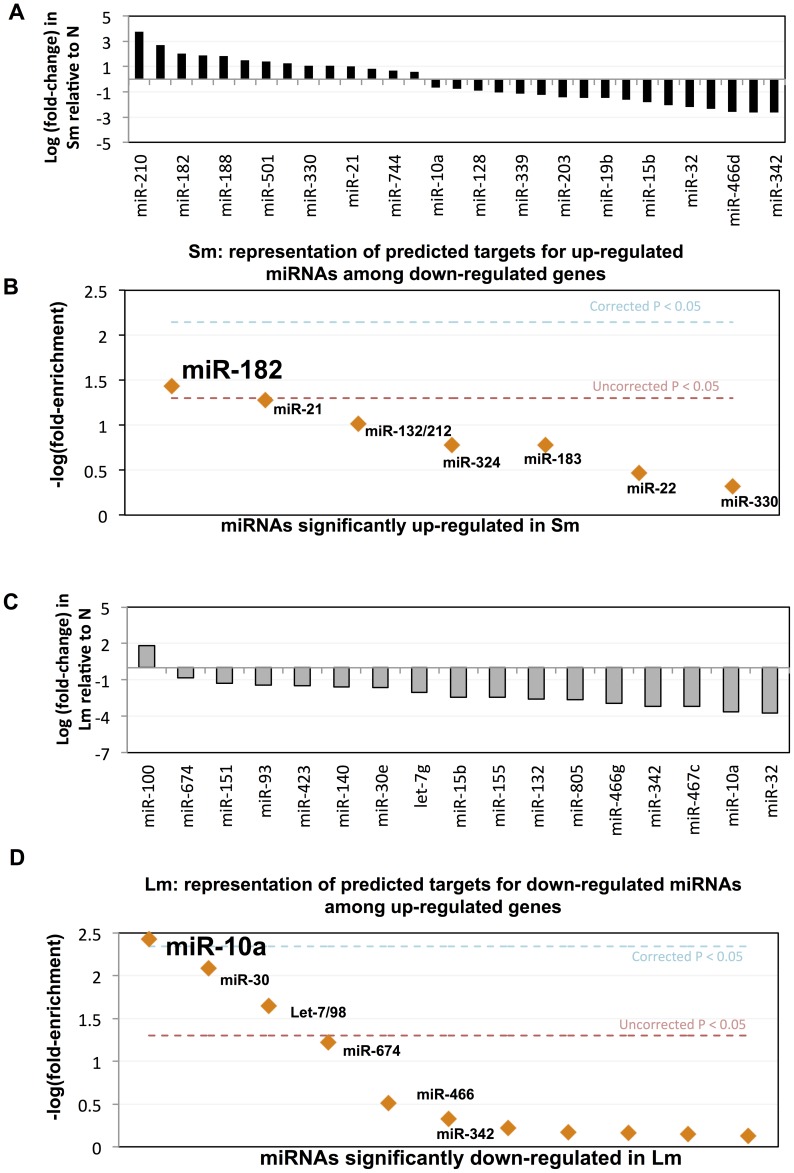
Differentially expressed miRNAs and candidate miRNA regulatory hubs in Th1- and Th2-Treg cells. miRNAs with significantly altered expression following (A) *Schistosoma mansoni* (Sm) or (C) *Leishmania major* (Lm) infection (Student's t-test p-value<0.05); y-axis: log (fold-change) of miRNA expression level. Representation of predicted targets for up-regulated miRNAs among down-regulated genes following *S. mansoni* (Sm) infection (B). Representation of predicted targets for down-regulated miRNAs among up-regulated genes following *L. major* (Lm) infection (D). Y-axis: −log of the empirical p-value of predicted target site enrichment over background expectation. Orange: miRNAs predicted to target differentially expressed genes significantly more than expected by chance, full details in Table S4. Dashed line: p-value = 0.05.

To identify miRNAs that might contribute to the different expression profiles, we deep sequenced small RNA species from each of the three Foxp3^+^ populations (*S. m*., *L. m*. and Naïve) and obtained 12–22 million reads in each sample (**Table. S2**). Within *S. mansoni* Foxp3^+^ cells, 31 miRNAs were differentially expressed (p<0.05) (**Figure 2A** **and Table. S3**). HIF1α-inducible miR-210 and 2 poly-cistronic miRNAs, miR-183 and IL-2-inducible miR-182, were the most significantly up-regulated (**Figure 2A**). Seventeen miRNAs were differentially regulated (p<0.05) in *L. major* Foxp3^+^ cells. Only one of these miRNAs, miR-100, was up-regulated; while miR-32 and miR-10a were the two most significantly down-regulated (**Figure 2C** **and Table. S3**). Notably, down-regulation of miR-10a in *L. major* Foxp3^+^ cells was relative to ‘naïve’ Foxp3^+^ Treg cells, and not relative to naïve T cells, as recently reported [40]. Several miRNAs were differentially expressed in both Foxp3^+^ populations, including miR-151, miR-30e, miR-15b, miR-132, miR-342, miR-10a and miR-32; however, not always in the same direction. For example, miR-132, which regulates interferon-stimulated genes [41], was ∼2-fold up-regulated in *S. mansoni* Foxp3^+^ cells, but ∼6-fold down regulated in *L. major* Foxp3^+^ cells.

We next employed *in silico* Monte Carlo simulation analyses to identify which, if any, of the up- or down-regulated miRNAs in each Foxp3^+^ population are predicted to target significantly more of the down- or up-regulated mRNA transcripts, respectively, than expected by chance (i.e. ‘regulatory hub’ miRNAs) [29]. This approach identified miR-182 (up-regulated in *S. mansoni* Foxp3^+^ cells) as the strongest candidate regulatory hub of the network of down-regulated genes in *S. mansoni* Foxp3^+^ cells (**Figure 2B** **and Table S4**), and miR-10a (down regulated in *L. major* Foxp3^+^ cells) as the strongest candidate regulatory hub of the network of up-regulated genes in *L. major* Foxp3^+^ cells (**Figure 2D**).

### miR-182 and miR-10a target distinct, non-overlapping genes in Foxp3^+^ cells

**Figure 3:**
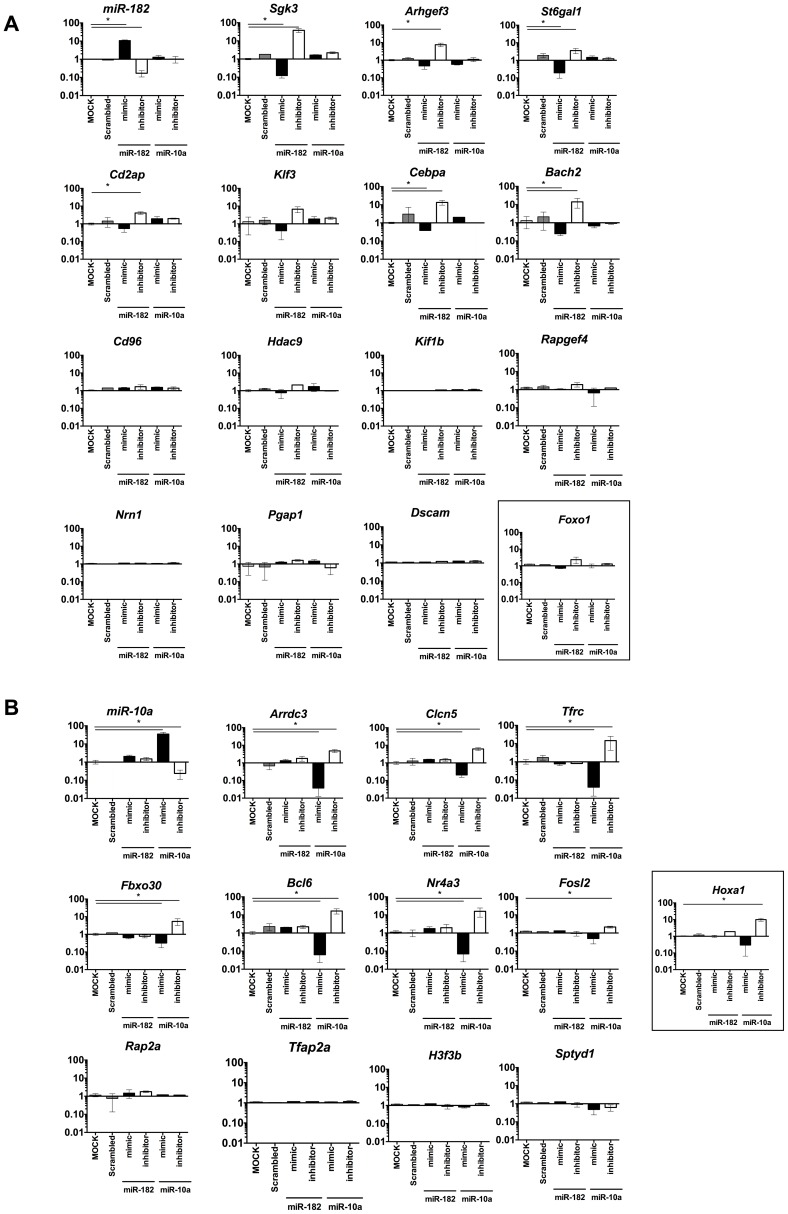
miR-182 and miR-10a target a significant number of *in-silico* predicted targets in Foxp3^+^ Treg cells. CD4^+^Foxp3^+^ cells isolated from naïve mice and transfected with miR-182 mimics or hairpin inhibitors (A) or miR-10a mimics or hairpin inhibitors (B) to identify predicted target gene regulation. RNA was extracted 24 hours post transfection for analysis. One of 3 individual experiments shown, with 3 biological replicates in each experiment. p-value = 0.05. with data expressed as mean ±SEM.

To validate the predicted target genes of miR-182 and miR-10a, we isolated primary Foxp3^+^ cells (predominantly nTreg cells), over-expressed or inhibited miR-182 or miR-10a using miRNA mimics or hairpin inhibitors, and measured miRNA and target mRNA expression. Transfection at >80% efficiency (**Figure S2**) increased (20-fold) or decreased (10-fold) miR-182 using specific mimics or inhibitors (**Figure 3A**). In contrast to naïve T cells [42], expression of a previously reported miR-182 target, *Foxo1*, was only marginally regulated by miR-182 in Treg cells failing to reach statistical significance (**Figure 3A** **, boxed**). Of the 14 predicted targets in *S. mansoni* Foxp3^+^ cells (**Table S5**), 6 were significantly regulated (>1.5 fold) in response to miR-182 mimics or inhibitors. Similarly, miR-10a significantly regulated *Hoxa1*, a previously validated miR-10a target [43], along with 7 of the 11 genes in *L. major* Foxp3^+^ cells predicted to be targets of miR-10a (**Figure 3B** **and Table S5**). Collectively, using gain and loss of function for miR-182 and miR-10a in primary Foxp3^+^ cells, these data identify that miR-182 regulates 6 of the predicted genes identified in Th2-Treg cells and miR-10a regulates 7 of the predicted genes identified within Th1-Foxp3^+^ cells.

### Foxp3^+^ Treg cells recruited to Th2 or Th1-mediated airway inflammation up-regulate miR-182 and down-regulate miR-10a, respectively

**Figure 4:**
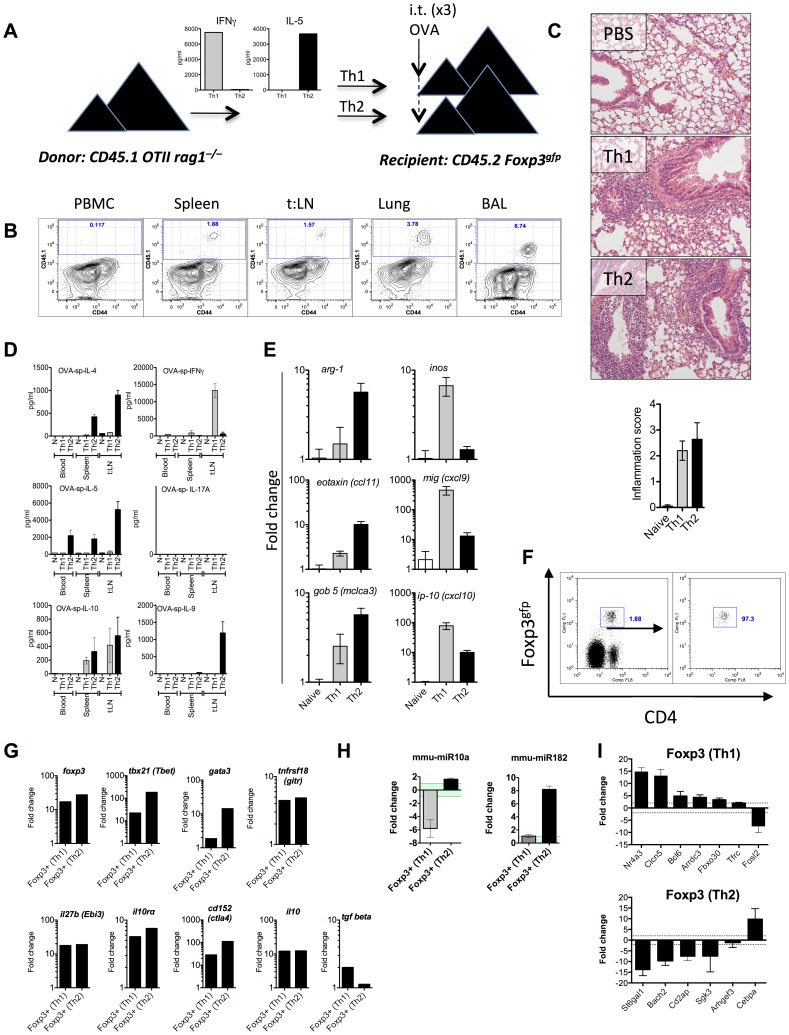
Differential miR-182 and miR-10a expression in Foxp3^+^ Treg cells isolated from Th2 or Th1-mediated pulmonary inflammation. (**A**) Naïve T cells (CD4^+^CD44^−^CD62L^hi^CD25^−^) from C57BL/6 CD45.1 OTII RAG2^−/−^ were polarised under Th1 or Th2 conditions and adoptively transferred into naïve CD45.2 Foxp3^gfp^ mice. (**B**) Donor cells were enumerated in the circulation (PBMC), spleen, thoracic lymph nodes (t:LN), lung tissue and broncho-alveolar air spaces (BAL). (**C**) Th1- and Th2-mediated pulmonary pathology was determined in H&E stained lung sections. (**D**) PBMC, splenocytes and local lymph nodes were re-stimulated with OVA for 3 days with cytokines measured in the supernatant after 3 days. (**E**) RNA was isolated from pulmonary tissue and gene expression analysed by qRT-PCR and expressed as fold change relative to naïve mice. RNA was isolated from recipient CD4^+^Foxp3^gfp+^ cells (**F**) with mRNA (**G and I**) and miRNA (**H**) expression analysed by qRT-PCR and expressed relative to RNA isolated from CD4^+^Foxp3^gfp+^ cells from naïve mice. Data expressed as mean ±SEM. Data presented **in A, B and G** are representative of one of three individual experiments.

To validate the functional significance of these miRNA:target interactions, and to determine whether differential expression of miR-182 and miR-10a was restricted to Foxp3^+^ cells from *S. mansoni* and *L. major* infections, we developed a Th1 and Th2-driven airway inflammation model. This system allowed us to eliminate pathogen influences, tissue-specific responses and any other factors that may have contributed to the observed Treg profiles observed above. Briefly, naïve T cells (CD4^+^CD44^lo^CD62L^hi^CD25^−^) from congenic and transgenic C57BL/6 mice (CD45.1^+^OTII^+^RAG2^−/−^) were polarized in vitro under Th1 or Th2 conditions, secreting high levels of IFNγ or IL-5 respectively (**Figure 4A**), and adoptively transferred into C57BL/6 CD45.2 Foxp3^gfp^ mice. One-day prior to transfer (d-1) and one and three days following transfer (d1 and d3), recipient mice received an intra-tracheal delivery of OVA into the lower airways (**Figure 4A**). Adoptively transferred cells migrated to the lung and broncho-alveolar (BAL) spaces (**Figure 4B**) and caused peri-bronchial and peri-vascular inflammation (**Figure 4C**). Antigen recall assays demonstrated that recipients of Th1 cells produced IFNγ and IL-10 (**Figure 4D**) and increased the expression of *Inos*, *Mig (Cxcl9)* and *Ip-10 (Cxcl10)* within the lung (**Fig. 4E**). Mice that received Th2 cells secreted IL-4, IL-5 and IL-9 (**Figure 4D**) and up-regulated *Arg1*, *Eotaxin (Ccl11)* and *Gob5 (Clca3)* within the lung (**Figure 4E**), characteristic of Th1 or Th2-mediated airway inflammation. CD4^+^Foxp3^+^ cells isolated from Th1- or Th2-inflammed lungs (**Figure 4F**) up-regulated *Tbx21, Gata3, Foxp3, Ctla4, Gitr (Tnfrsf18), Il10rα, Ebi3* and *Il10 with a small increase in Tgfβ* in Th1-Treg cells only (**Figure 4G**). As predicted, Foxp3^+^ cells from Th1 inflamed lungs down-regulated miR-10a with no change in miR-182 (**Figure 4 H**), similar to Foxp3^+^ cells from *L. major* infected mice (**Figure 2**). Foxp3^+^ cells from Th2-inflamed lungs up-regulated miR-182, with a marginal increase in miR-10a, similar to Foxp3^+^ cells from mice infected with *S. mansoni* (**Figure 2**). With the exception of *Fosl* and *Cebpa*, we also observed a very similar target gene expression profile in Th1-Treg or Th2-Treg cells isolated from the inflamed lung as compared to Treg cells from *L. major* or *S. mansoni* infected mice (**Figure 4I**).

These data support the notion that down regulation of miR-10a and up-regulation of miR-182 within Foxp3^+^ cells is associated with Th1 or Th2 biased immune environments, respectively.

### Th1-, but not Th2-, associated Treg cells can efficiently suppress both Th1 and Th2 cells

To test whether Th1 and Th2-associated Treg cells were functionally distinct from each other, we fluorescently-labeled OVA-specific Th1 or Th2 Teff (CD4^+^CD44^+^Foxp3^−^) cells isolated from Th1- or Th2-inflamed lungs, or naïve Teff cells as a control population, and co-cultured these cells with Th1- or Th2-Treg cells (CD4^+^Foxp3^+^) from respective Th1 or Th2-inflamed lungs, or with Treg cells isolated from the opposing inflammatory environment in a series of ‘cross-over’ assays. In these assays, Th1-Treg cells potently suppressed Th1-Teff cells (**Figure S3A**) and Th2-Teff cells (**Figure S3B**), whereas Th2-Treg cells only suppressed Th2 cells and not Th1 cells (**Figure S3C and S3D**).

### miR-182 and miR-10a are required for Foxp3^+^ Regulatory T cell-mediated suppression of Th2 and Th1 cell proliferation in vitro, respectively

**Figure 5:**
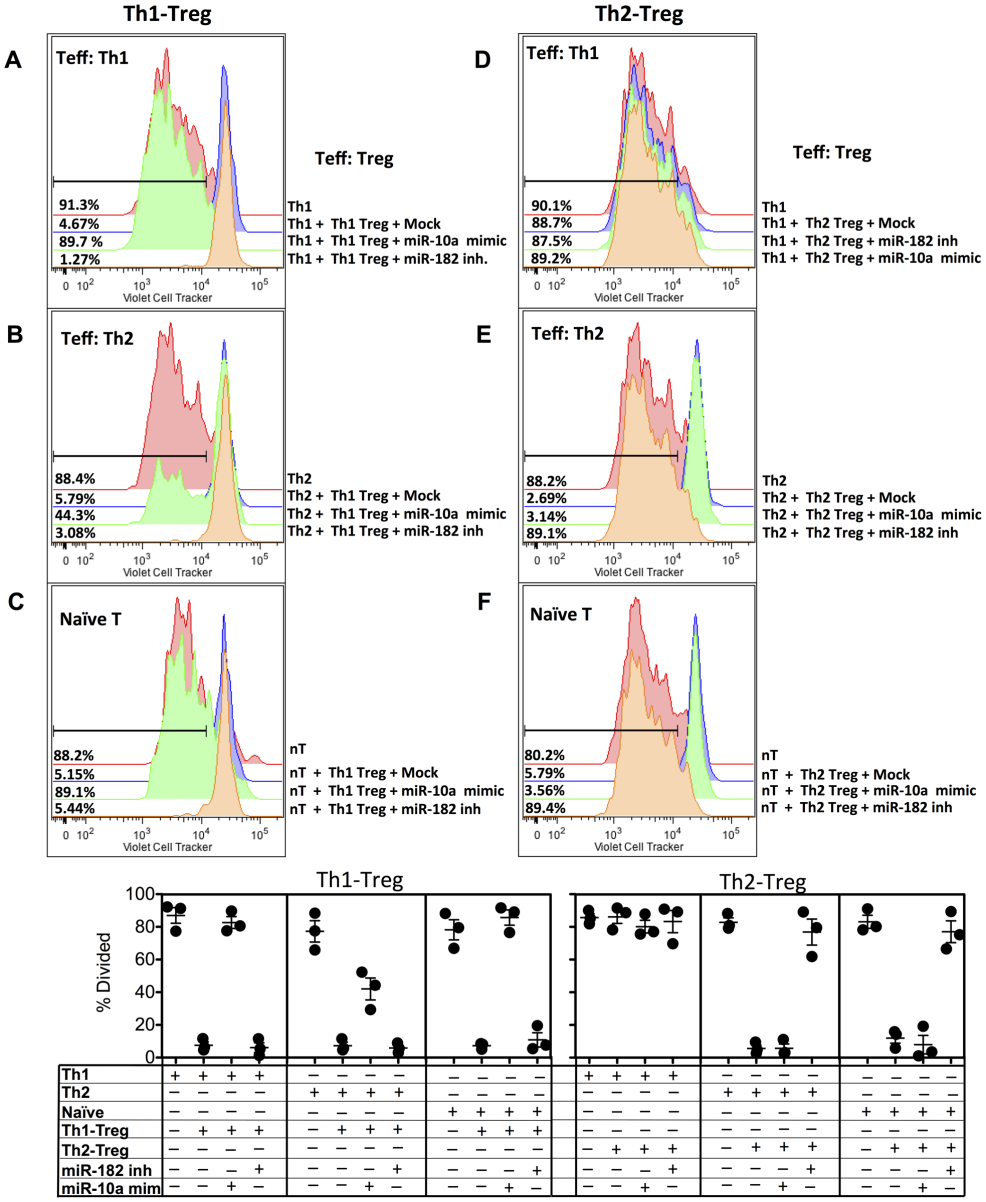
Up-regulated miR-182 and down-regulated miR-10a expression in Th2- and Th1-Treg cells Treg cells confers suppressive capacity in vitro. Th1 (A & D) or Th2 (B & E) T effector (Teff, CD4^+^CD44^+^Foxp3^gfp−^) and Th1-Treg (A, B & C) or Th2-Treg (D, E & F) (CD4^+^Foxp3^gfp+^) cells were isolated from the lungs of recipient mice, as in Figure 4. As a control, naïve T cells (C & F) were also isolated from the spleen of OTII mice. Teff or naïve T cells (10^4^) were labeled with cell trace violet (Invitrogen) and cultured alone, or in equal ratios (1∶1) with mock transfected Treg cells, Treg cells transfected with miR-10a mimics or Treg cells transfected with miR-182 inhibitors, as indicated, for 3 days with irradiated splenocytes (2×10^5^) and OVA (10 µg/ml). One of 3 individual experiments is shown, with technical replicates shown in the scatter plot. Add statistical test results?

We next tested whether down-regulated miR-10a and up-regulated miR-182 was functionally required for Th1- and Th2-Treg-mediated suppression, respectively. Th1-Treg cells isolated from the lungs of mice were transfected with miR-10a mimics (**Figure S3E**), to overturn the down-regulated miR-10a observed in Th1-Treg cells (**Figure 4H**). Following the observation that miR-182 was upregulated in Th2-associated Foxp3^+^ cells (**Figure 4H**), Th2-Treg cells were transfected with miR-182 hairpin inhibitors (**Figure S3E**). Mock-transfected Th1-Foxp3^+^ cells efficiently suppressed Th1 (**Figure 5A**), Th2 (**Figure 5B**) and naive (**Figure 5C**) T cell proliferation. However, Th1-Treg cells transfected with miR-10a mimics were compromised in their ability to suppress Th1 cells (**Figure 5A**) and naïve T cells (**Figure 5C**), but retained the ability to partially suppress Th2 cells (**Figure 5B**). As a further control, we transfected Th1-Treg cells with miR-182 inhibitors, as miR-182 was not differentially regulated in Th1-Treg cells (**Figure 4H**) and this did not influence Th1-Treg mediated suppression of Th1, naïve or Th2 cells (**Figure 5A, B and C**). Th2-Treg cells were unable to suppress Th1 cells (**Figure 5D**) but were fully capable of suppressing Th2 (**Figure 5E**) and naïve T cells (**Figure 5F**). Transfection with miR-10a mimics had no impact on Th2-Treg mediated suppression. However, Th2-Treg cells transfected with miR-182 inhibitors compromised their ability to suppress Th2 and naïve T cell proliferation, indicating that elevated miR-182 was required for Th2-Treg function. Treg cells isolated from the spleen of naïve animals were unable to control OVA-specific Th1 or Th2 cells (**Figure S4A and S4B**), but were fully capable of suppressing naïve T cells (**Figure S4C**). Transfection of Treg cells from naïve mice with miR-182 inhibitors or miR-10a mimics also compromised their suppressive capacity. Taken together, these data indicate that down-regulation of miR-10a is critically required for Th1-Treg cells to control Th1 cells and naïve T cells, while up-regulated miR-182 is required for Th2-Treg-mediated suppression of Th2 cells and naïve T cells, highlighting the divergence of these two Treg populations, while Treg cells from naïve mice were dependent upon both tightly regulated miR-10a and miR-182.

### IL-4 regulates *cMaf* and miR-182, while IL-12/IFNγ regulates *Creb* and miR-10a in natural and inducible Treg cells

To determine the upstream factors that may contribute to miR-182 and miR-10a expression in Treg cells, we screened for transcription factor binding sites in the promoters of the primary transcripts of both miR-182 and miR-10a using Pwm-Scan (as described in the methods). We identified putative binding sites in the miR-182 promoter for IL-4-regulated transcription factors (TFs), including cMaf, and IL-12/IFNγ-regulated TFs, including Creb, in the miR-10a promoter (**Figure S5A**). Concordant with the *in-silico* predictions, exposure of natural (nTreg) or in vitro generated inducible Treg (iTreg) cells (**Figure S5B**) to IL-4, mimicking a Th2 environment, up-regulated *cMaf* (**Figure S5C**), and miR-182 (**Figure S5E**), similar to ex vivo Th2-Treg cells (**Figure 2** **, and Table S1**). IL-12/IFNy treatment of nTreg and iTreg, mimicking the Th1 environment, down-regulated *Creb* (**Figure S5D**) and miR-10a (**Figure S5F**) in Treg cells, relative to naïve T cells, pheno-copying miR-10a expression in ex vivo Th1-Treg cells (**Figure 2**). Following recent studies indicating that Foxp3-mediated epigenetic modifications may be altered in Foxp3^gfp^-reporter mice [44], [45], we compared miR-182 and miR-10a expression in freshly isolated nTreg cells and in vitro generated iTreg cells from Foxp3^rfp^ and Foxp3^gfp^-reporter mice, but did not observe any appreciable difference in miR-182 or miR-10a expression, relative to naïve T cells (**Figure S6**).

### miR-182 and miR-10a control IL-2 and IFNγ in Treg cells

CD2, via Cd2ap and BACH2, regulates IL-2 production through direct binding to the IL-2 promoter [30], [31]. Following the observation that miR-182 targeted Cd2ap and Bach2, and that IL-4 regulated miR-182 (**Figure S5**), we tested whether IL-4 influenced the expression of miR-182, *Bach2, Cd2ap* and subsequent IL-2 production. IL-4 treated nTreg or iTreg cells had reduced *Bach2* and *Cd2ap* relative to naïve T cells or untreated Treg cells (**Figure S7A, S7B**). We therefore assayed for IL-2 following IL-4 treatment, to determine whether IL-4-regulated miR-182, and subsequent changes in *Bach2* and *Cd2ap* had any influence on IL-2 responses. *Il2* mRNA and protein levels were not altered following IL-4 treatment alone (**Figure S7E**), however inhibition of miR-182, with or without IL-4 treatment, led to a 50-fold induction of *Il2* transcription and IL-2 secretion (**Figure S7E**). These data indicate that miR-182 controls IL-2 production in Treg cells, possibly via *Cd2ap* and *Bach2*, and that IL-4 re-enforces miR-182-mediated control of IL-2.

Previous reports have identified that Nr4a3 induces Foxp3 expression and represses IFNγ [46]. Following the observation that miR-10a targeted Nr4a3 we assayed for IFNγ following miR-10a over expression, with or without IL-12/IFNγ treatment.

IL-12/IFNγ treatment alone induced IFNγ in Treg cells (4-fold, **Figure S7F**), similar to previous reports [47], [48], however IFNγ was increased 40-fold when combined with miR-10a over-expression (**Figure S7F**). Interestingly, miR-10a over-expression alone also led to an increase in IFNγ (9-fold). Thus, type-2 regulated miR-182 and type-1-regulated miR-10a, respectively, contribute to the regulation of IL-2 and IFNγ responses in Th2- and Th1-Treg cells.

### Down-regulation of miR-10a and up-regulation of miR-182 is essential for Foxp3^+^ Regulatory T cell-mediated control of Th1- or Th2-driven airway inflammation, respectively

**Figure 6:**
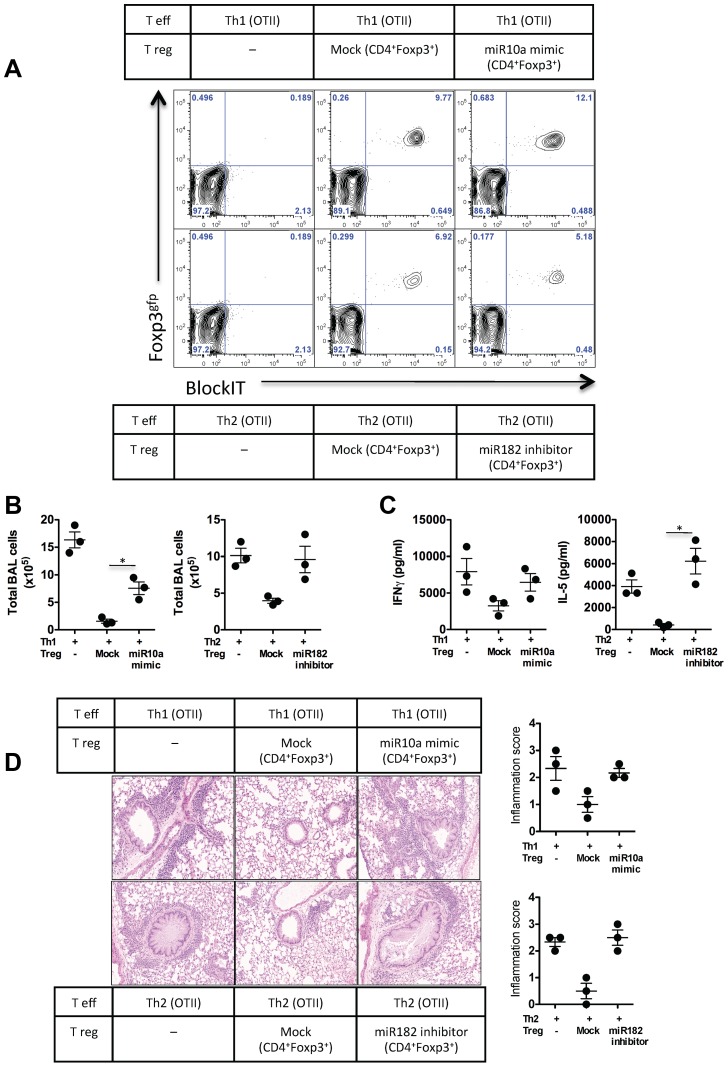
Up-regulated miR-182 and down-regulated miR-10a expression in Foxp3^+^ cells is required for Th2-Tregs to suppress Th2 response and Th1-Tregs to suppress Th1 response in vivo, respectively. In vitro-polarised Th1 or Th2 T effector (Teff) cells were adoptively transferred with Th1-Treg (CD4^+^Foxp3^gfp+^) or Th2-Treg isolated from the lungs of mice with Th1 or Th2-driven pulmonary inflammation, respectively and as indicated. Co-transferred Foxp3+ Treg cells were either mock transfected or transfected with miR-10a mimics or miR-182 inhibitors, as indicated. (A) Recruitment of donor Treg cells to the lungs of recipient mice, as a percentage of total CD4^+^ cells in the lung. (B) Broncho-alveolar infiltrates of mice one day following final OVA challenge. (C) Local lymph nodes were isolated and re-stimulated with OVA. IFNγ or IL-5 was measured in the supernatants after 3 days. (D) Lungs were removed, sectioned and stained with H&E for pathology. One of 2 individual experiments shown. p-value = 0.05 with data expressed as mean ±SEM.

To determine whether miR-10a and miR-182 was required for Treg survival, migration and control of Th1 and Th2-mediated inflammation in vivo, we designed a double adoptive transfer system (**Figure S8**). Briefly, Th1- or Th2-associated Foxp3^+^ Treg cells were isolated from Th1 or Th2–inflamed tissue, as above (**Figure 4**). A second recipient mouse received Teff (OTII-Th1 or OTII-Th2) cells alone or a combination of mock-transfected Treg cells, miR-10a mimic transfected Th1-Treg cells with Th1-Teff cells, or miR-182-inhibitor transfected Th2-Treg cells with Th2-Teff cells. Following intra-tracheal delivery of OVA, similar percentages of transferred Treg cells were observed in the lung of recipient mice (**Figure 6A**), indicating that all Treg cells experienced similar survival irrespective of transfection treatments. Significant numbers of inflammatory cells were recovered from the airspaces of mice receiving Th1 or Th2 cells (**Figure 6B**), however the co-transfer of mock-transfected Treg cells significantly reduced the number of inflammatory cells. Co-transfer of Th1 cells and miR-10a mimic transfected Th1-Treg cells, or Th2 cells with miR-182-inhibitor transfected Th2-Treg cells failed to suppress inflammatory cell recruitment. The requirement for down-regulated miR-10a in Th1-Treg cells and up-regulated miR-182 in Th2-Treg cells was also reflected by uncontrolled IFNγ or IL-5 secretion in re-stimulated lymph nodes, compared to mice receiving mock-transfected Treg cells (**Figure 6C**). Mock-transfected Treg cells potently reduced pulmonary pathology (interstitial inflammation, mucus plugs and epithelial elongation), which was compromised when miR-10a or miR-182 was specifically deregulated in Th1- or Th2-Tregs, respectively (**Figure 6D**). Taken together these studies highlight two diverse Foxp3 populations that develop to control Th1 or Th2 inflammatory events. The molecular programs in these Foxp3^+^ Tregs are in-part regulated by distinct upstream regulatory miRNA hubs, miR-182 and miR-10a, which target non-overlapping and essential genes within these diverse Foxp3^+^ populations.

## Discussion

In this study we identified distinct populations of Foxp3^+^ Treg cells recruited to Th1 or Th2 inflammatory environments expressing unique gene and miRNA profiles. Several genes and miRNAs were comparably regulated between the two subsets including miR-30e, miR-15b, miR-32, miR-151 and miR-342, with other miRNAs highlighting a clear divergence. For example miR-132 was significantly down regulated in Foxp3^+^ cells from Th1 rich surroundings (−2.56 fold) and up regulated in Foxp3^+^ cells in Th2 environments (+2.09 fold). Using miRNA target prediction algorithms and Monte Carlo simulations we identified two miRNA regulatory hubs that target multiple genes contributing to the divergent gene expression profiles. Specifically, Th1 inflammation, following chronic *L. major* infection or acute Th1-induced inflammation, recruited Foxp3^+^ Treg cells that up-regulated a suite of genes regulated by miR-10a. In contrast, Foxp3^+^ cells isolated from Th2 environments following chronic S. *mansoni* infection or acute Th2-driven inflammation down-regulated a suite of genes under the control of miR-182. These data support the notion that Foxp3^+^ cells are heterogeneous, or adaptable to their inflammatory environment [10]–[12], [14], [49] and provide an upstream molecular mechanism contributing to Foxp3^+^ heterogeneity.

Previously, T-bet has been singled out as a co-transcription factor required for Treg cells to control anti-mycobacterial Th1 responses [12]. In our studies, Foxp3^+^ cells isolated from *L. major* infected tissue did not up-regulate *T-bet*, which may be explained by different infections, different stages of infection or different tissues studied. In support of the latter, and in agreement with the previous study, Foxp3^+^ cells isolated from Th1-inflamed lung tissue up-regulated T-bet (∼20-fold, **Figure 4G**), similar to pulmonary *M.Tb*. Foxp3^+^ cells. Interestingly, T-bet was greater than 200-fold up-regulated in Foxp3^+^ cells isolated from Th2-driven inflammation or from the liver of *S. mansoni* infected mice (2.6-fold). If Foxp3^+^T-bet^+^ cells are potent suppressors of Th1 responses, it is tempting to speculate that Foxp3^+^T-bet^+^ cells contribute to a dominant Th2 environment by potently suppressing Th1 responses. Similarly, Irf4, a transcription factor involved in several T helper cell subsets [50], [51], was recently identified in Foxp3^+^ cells restraining Th2 responses. Irf4 however was not up-regulated in Th2-associated Treg cells isolated from schistosome infected mice, relative to Foxp3^+^ cells from the spleen of naïve mice, and was only slightly up regulated in Th1-associated Foxp3^+^ cells (1.68-fold). Strinkingly, Th1- Foxp3^+^ cells up-regulated a collection of transcriptional regulators, including Stat-3 (1.98 fold), *Bcl6* (1.80-fold), *Ap1* (2.14 fold) and *Runx2* (2.02 fold). Similarly, *Th2-derived* Foxp3^+^ cells co-expressed *Blimp1* (3.78 fold), *Tbx21* (T-bet) (2.64), *Hif2α* (2.08 fold), *E4bp4* (1.91 fold), *Runx2* (1.68 fold) and *Egr2* (1.60 fold). These data suggest that there is either significant heterogeneity, or plasticity, within Foxp3^+^ populations [52] or that co-opting multiple transcription factors is common and does not restrict control to one particular T helper subset, but rather broadens regulatory function. Indeed, Treg cells isolated from Type-1 inflamed tissue had the capacity to suppress Th1 and Th2 cells, while Th2-Treg cells could only control Th2 cells. We hypothesize that suppression of Th2 cells by Th1-Treg cells could be mediated by TGF-β, which was slightly elevated in Th1-, but not Th2-, Treg cells (**Figure 4G**) and can potently inhibit Th2 cells [53]. However, given that TGFβ is highly regulated post-translationally, surface bound or secreted bioactive TGFβ may not be increased. Alternatively, the continued ability of Th1-Treg cells to control Th2 cells, but not Th1 cells, following over-expression of miR-10a, is most likely due to the increased IFNγ, which can also inhibit Th2 cell responses.

Computational analysis [29] identified miR-182 in Th2-Foxp3^+^ cells and miR-10a in Th1-Foxp3^+^ cells as potential regulatory miRNA hubs, which targeted multiple differentially regulated genes. We focused on miR-182 and miR-10a for functional studies, as these were the top candidate regulatory hubs from the Monte Carlo analyses in Foxp3 cells from infected mice. In support of this, down regulated miR-10a and up-regulated miR-182 was also observed in Foxp3^+^ cells isolated from Th1- or Th2-inflammed lungs, analogous to the chronic infection studies.

It was recently demonstrated that IL-2/STAT5 regulated miR-182 in helper and regulatory T cells [42] targeting *Foxo1* and permitting helper cell proliferation. Despite the high consumption of IL-2 by Foxp3^+^ T cells and the requirement for *Foxo1, and Foxo3*, for Treg cell survival and function [54], [55], a role for miR-182 in Treg cells was not thoroughly investigated. Our systematic approach identified putative binding sites in the promoter of miR-182 for the IL-4-regulated transcription factor, *cMaf*. In agreement with this, IL-4-treated Treg cells up-regulated *cMaf*, similar to previous reports in macrophages and T cells [56], [57]. Unlike naïve T cells, which produce IL-4 and IL-2 and up-regulate *cMaf* following IL-4 treatment, Treg cells did not produce IL-4 (data not shown) or IL-2, in part through a miR-182-dependent pathway. The phosphorylation state of cMaf, additional pathways including IL-2 [42] and other transcriptional regulators may also contribute to miR-182, as *cMaf* transcript levels in untreated iTreg and nTreg were indistinguishable from naive T cells, despite elevated miR-182. Nevertheless, IL-4-treated Treg cells up-regulated *cMaf* and miR-182, in line with other studies identifying that IL-4-treated human [58], [59] and murine [60] Treg cells develop distinct and potent suppressive phenotypes. The precise mechanism from these studies, however, was unclear.

It has long been appreciated that anergic and regulatory T cells do not produce IL-2, through reduced JNK and ERK signaling [61] and remodeling of the *Il2* locus [62]. We identified two miR-182-regulated genes that can control IL-2 production, *Bach2*, a basic leucine zipper transcription factor [30] and *Cd2ap* [31]. As predicted, the up-regulation of *cMaf* and miR-182 by IL-4 led to a reduction of *Bach2* and *Cd2ap* expression in Treg cells (**Figure S5**), with no IL-2 production. Disrupting this pathway, through inhibition of miR-182, led to an increase in *Bach2* and *Cd2ap* and a significant increase in transcription and secretion of IL-2, indicating that IL-2 is critically regulated by miR-182, potentially via control of *Bach2* and *Cd2ap*. Although other important molecular pathways are under the control of miR-182, including those controlled by *C/EBPα*, *Arhgef3* and *Hdac9* which are also intimately involved in Treg biology [63]–[66], together with previous reports, we propose that IL-2 and IL-4 reinforce a negative feedback loop in Treg cells, with IL-2 induced [42] and IL-4-re-enforced miR-182 inhibiting IL-2 secretion.

miR-10a was up-regulated in ex vivo Treg cells and naïve T cells polarized into iTreg with TGFβ in vitro [40], [67]. We also observed an increase in miR-10a in ex vivo nTreg and iTreg cultures, relative to naïve T cells. However, our study design identified that miR-10a was subsequently reduced in Treg cells in Th1 environments. Whether splenic nTreg cells migrate to peripheral sites or de-novo iTreg cells respond to inflammatory events is unclear. To investigate the pathways involved in miR-10a regulation, we identified several putative TF binding sites in the miR-10a promoter, including the TGF-β [68], IL-2 [69], IL-12 [70] and IFNγ [71]-regulated transcription factor, CREB. CREB stabilizes Foxp3 in Treg cells [72] and is inhibited by IFNγ [71], [73], [74]. Creb expression was slightly elevated in ex vivo nTreg and in vitro-generated iTreg cells, relative to naïve T cells, but was successively decreased, below naïve T cell levels, following exposure to type-1 inflammatory signals, IL-12 and IFNγ. Furthermore, miR-10a followed a similar expression pattern as *Creb*, with reduced miR-10a following IL-12/IFNγ treatment, suggesting that Creb expression may influence miR-10a. Although multiple factors can influence miR-10a and Creb expression, these data indicate that Treg cells undergo dynamic molecular modifications upon exposure to various inflammatory signals, in this case along an IL-12/IFNγ, *Creb*, miR-10a axis.

We identified several miR-10a-regulated genes in Foxp3^+^ cells, including *Arrdc*, an α-arrestin family member that degrades phosphorylated integrin β4 (CD104) [75] and β2-adrenergic receptors [76], two pathways required for the development [77] and survival [78] of Foxp3^+^ T cells. miR-10a also regulated the transcriptional repressor, *Bcl6*, an important pathway recently identified in iTreg cells, preventing iTreg conversion in to T_FH_ cells [40]. Furthermore, co-expression of Bcl6 with Blimp1, Cxcr5 and PD-1 (Pdcd1) in Foxp3^+^ in Treg cells identified as T_FH_-Reg cells, have also been reported [11], [13]. Dissimilar to these studies we did not observe a T_FH_-Reg, or T_FH_ phenotype, as phenotypic markers of T_FH_ cells, beyond Bcl6, were reduced or unchanged (*Cxcr5* −3.22-fold, *Btla* −2.0 fold, unchanged *Il21*, *Cd40l*, *Cd200*, *Cd30l*, *Cd57*, and *Fyn*). The relatively subtle changes in miR-10a and Bcl6 in Th1-Treg cells may retain Treg function, without conversion into TFH cells, or T_FH_-Reg cells. For example, we observed that miR-10a was reduced 3.5-fold in Th1-Treg relative to naïve Treg cells, in contrast to the study identifying iTreg cell conversion into TFH cells [40] when iTreg cells were transduced with a miR-10a sponge to significantly sequester miR-10a. Similarly, we observed a relatively subtle increase in *Bcl6* (1.79-fold, **Table S1**) compared to the ∼10-fold increase in T_FH_-Reg cells [11], [13].

In addition to *Bcl6*, we identified *Fbxo30* (also known as *Fbxw7* and *Fbw7*) and the TGFβ-signaling molecule, *Nr4a3* [79], [80] as miR-10a-regulated genes in Th1-Treg cells. Conditional deletion of *Fbxw7* in CD4^+^ cells [81], or deletion of *Nr4a3* and the closely related *Nr4a1*, resulted in hyper-proliferation of T cells, thymic lymphoma's and lethal lymphoproliferation [82], a phenotype similar to *Foxp3* ^−/−^ mice. Furthermore, ectopic expression of *Nr4a3* induced Foxp3 expression and repressed IFNγ production [46]. IL-12/IFNγ treatment, which reduced *Creb* and miR-10a expression, resulted in a small increase in miR-10a-regulated genes, Fbxo30 and Nr4a3 and a small increase in *Ifnγ* transcription. Similar observations have been made in mouse and human Treg cells, with IL-12-treatment converting Foxp3^+^ cells into IFNγ^+^Foxp3^+^ cells [47], [48]. Disrupting this molecular pathway, by over-expressing miR-10a, coupled with IL-12/IFNγ treatment, dramatically increased *Ifnγ* transcription, indicating that reduced miR-10a permitted tight control over IFNγ in Treg cells, possibly via Nr4a3 [46]. IFNγ secretion by Th1-Treg cells transfected with miR-10a mimics provides a plausible explanation as to how Th1-Treg cells retained their ability to partially control Th2 cells following miR-10a manipulation. Collectively, we have identified a suite of miR-10a targets in Th1-Foxp3^+^ cells, which regulate G-protein coupled receptor function (*Aardc3*), gene transcription (*Bcl6*), ion transport (*Clcn5* and *Rap2a*), iron metabolism (*Tfrc*) and TGF-β signaling (*Fbxo30/Fbxw7* and *Nr4a3*). Furthermore, we have identified a mechanistic pathway of IL-12/IFNγ-regulated miR-10a expression that critically controls IFNγ production in Treg cells.

In summary, Th1- or Th2-associated Foxp3^+^ cells developed distinct molecular profiles, influenced by local cytokine signaling pathways. IL-12/IFNγ-influenced miR-10a controlled subsequent IFNγ production in Th1-Treg cells, while IL-4-regulated miR-182 critically prevented IL-2 production in Th2-Treg cells. In addition, we propose that miR-182 and miR-10a function as regulatory hubs, coordinating a variety of pathways in Th2-Treg and Th1-Treg cells. These data strongly support the concept that different Foxp3^+^ cells activate distinct gene programs, shaped by different inflammatory signals. We also provide evidence for an upstream miRNA-mediated pathway regulating Foxp3^+^ cell specialization and functional stability.

## Materials and Methods

### Animals

Female C57BL/6, C57BL/6 CD45.2 Foxp3^gfp^ [83], C57BL/6 Foxp3^rfp^ [84], C57BL/6 CD45.1 OTII RAG2^−/−^ 6–8 weeks' old were bred and kept in the specific pathogen–free facility at the National Institute for Medical Research, or National Institutes of Health.

### Ethics statement

All animal experiments were approved by UK National Institute for Medical Research Ethical Review Panel and NIAID animal care and use committee and carried out according to institutional guidelines (UK National Institute for Medical Research Ethical Review Panel), UK Home Office regulations (Project licence no. 80/2506) and according to The NIAID animal care and use committee in accordance with the recommendations in the Guide for the Care and Use of Laboratory Animals of the National Institutes of Health. A minimum of 5 mice per group was used in each experiment, unless indicated.

### Parasites and experimental infections

Percutaneous infections were carried out with 35 *S. mansoni* cercariae (Biomedical Research Institute, Rockville, MD), as previously described [85]. Mice were infected in the ear dermis with 10^5^ *L. major* metacyclic promastigotes using a 27.5 G needle in a volume of 10 µl [38].

### FACS sorting, staining and analysis

Cells were isolated from infected or inflamed tissue by mechanical disruption followed by percoll gradient separation and were stained with anti-mouse CD4 (RM4-5, BD Biosciences, Pacific Blue (V450) or APC), CD3ε (17A2, BD Biosciences, FITC or Alexa flour 700), CD44 (IM7, BD Biosciences, PE-Cy7 or Alexa flour 700), CD25 (PC61, BD Biosciences, PE or FITC) and CD45.1 (A20, BD Biosciences, APC or PE) diluted in PBS with 0.1% FCS before analysis using a BD LSRII and TreeStar FlowJo.

### In-vitro suppression assay

For proliferation/suppression assays, 10^4^ Teff cells were labeled with cell trace violet (Invitrogen) as per manufacturers guidelines and stimulated with irradiated splenocytes (2×10^5^) and OVA (10 µg/ml) for 3 days in the presence or absence of Treg cells, at the indicated ratios before analysis using a BD LSRII and TreeStar FlowJo.

### RNA extraction, microarray and Next Generation sequencing

FACS purified cells were stored in RLT lysis buffer at −80°C until RNA was extracted. For mRNA analysis, RNA was extracted using RNeasy spin columns (Qiagen) followed by DNAse treatment. cDNA was generated from 5 ng of total RNA using WT-Ovation Pico system (version 1) RNA Amplification System followed by double stranded cDNA synthesis using WT-Ovation Exon Module. cDNA quality was determined using an Agilent BioAnalyzer and through hybridization performance on Affymetrix GeneChip mouse gene 1.0 ST arrays. For miRNA analysis, small RNA species (20–200 bp) were collected from the same samples and used for sequencing on the ABI SOLiD sequencer (Applied Biosystems, Santa Clara, CA). Hybridization, fluidics and scanning were performed according to standard Affymetrix protocols (http://www.affymetrix.com). GeneChip Operating Software (GCOS v1.4, http://www.Affymetrix.com) was used to convert the image files to cell intensity data (cel files). The array data were quantile normalized and analyzed using Partek Genomics Suite software (Partek, inc. St. Louis, Mo., v6.4-6.09.0129). We identified differentially expressed genes using ANOVA and t-tests. Genes with false discovery rate corrected p-values less than 0.1 and fold change values ≥1.5 were considered significant. The resulting data were analyzed with IPA (Ingenuity Pathway Systems, www.ingenuity.com). Libraries for SOLiD sequencing were prepared using the SOLiD Small RNA Expression Kit (Applied Biosystems) following the manufacturer's protocol. Templated beads for sequencing were prepared using a 1 pM library input following the Applied Biosystems SOLiD 3 Templated Bead Preparation Guide (Applied Biosystems, Foster City CA). Small RNA libraries were run on the ABI SOLID 3.0. Reads were mapped to Mus musculus microRNAs (miRBase v13.0) [86] using the Small RNA Analysis Tool v0.4 (Applied Biosystems). Read counts below 25 (including miR-96) were removed from further analysis with read counts between samples normalized based on the total number of uniquely mapped reads in each sample.

### Identification of miRNA regulatory hubs

Candidate miRNA regulatory hubs were identified using Monte Carlo simulation analysis as described previously [29]. First, we used the seed-based target prediction algorithm TargetScanS to determine for each miRNA the number of predicted targets among our gene set of interest (e.g. up/down-regulated transcripts in Foxp3^+^ cells in response to pathogen). We repeated this procedure 10,000 times with a new set of randomly selected genes from the genome each time, in order to generate a background expectation of the number of predicted target genes for each miRNA, which was then used to calculate an empirical p-value for the number of predicted target genes in the gene set of interest. To account for differences in the average 3′ UTR length between the genes of interest and the randomly selected genes in each simulation, the number of predicted target genes was normalized to the average 3′ UTR length.

### Prediction of transcription factor binding sites

The genomic locations of the miR-182 and miR-10a transcription start sites (TSS) were identified using previously published methods [87], [88]. We defined the promoter region as 1 kb upstream and 500 bp downstream of the TSSs. Within these promoters, we identified putative transcription factor binding sites using PWMSCAN [89], which searches for sequences that match any known transcription factor binding site motif recorded in TRANSFACv10.2. A match score with a p-value<5×10^−6^ was considered to be a high-confidence binding site prediction.

### Quantitative RT-PCR for mRNA and miRNA

RNA was isolated using RNeasy mini spin columns followed by miScript RT or Quantitect RT according to manufacturer's recommendations (Qiagen). Real-time RT-PCR was performed on an ABI Prism 7900HT Sequence Detection System (Applied Biosystems) with relative quantities of mRNA determined using SYBR Green PCR Master Mix (Applied Biosystems) and by the comparative threshold cycle method as described by Applied Biosystems for the ABI Prism 7700/7900HT Sequence Detection Systems. mRNA levels were normalized to HPRT and miRNA levels were normalized to RNU6B and then expressed as a relative increase or decrease compared with levels in controls.

### miRNA mimic and hairpin inhibitor transfection

Treg cells were isolated, as described above and transfected with 100 nM of miR-182 or miR-10a mimics or hairpin inhibitors (Thermo Scientific Dharmacon) or MOCK transfected using Nucelofection reagents according to manufacturer's recommendations (Amaxa). Ex-vivo nTreg cells were cultured in rIL-2 (10 ng/ml)-supplemented media for 24 hours before washing and use in suppression assays or transfer in-vivo. BlockiT fluorescent oligos (Invitrogen) were used to determine transfection efficiency. miRNA mediated impacts on mRNA expression was determined 24–48 hours post transfection.

### T cell polarisation, adoptive transfer, airway inflammation model

Naïve T cells (CD4^+^CD44^−^CD62L^hi^CD25^−^) were FACS purified and polarised under Th1 (IL-12, 10 ng/ml; anti-IL-4, 10 ug/ml)), Th2 (IL-4, 10 ng/ml; IL-2, 10 ng/ml; anti-IFNγ, 10 ng/ml) or iTreg (TGFβ, 10 ng/ml, Retinoic acid, 10 nM) conditions in the presence or absence of OVA-pulsed irradiated splenocytes as APC's for seven days, as indicated.

Freshly isolated nTreg or in vitro generated iTreg cells were washed and cultured with either IL-4 (10 ng/ml), IL-12/IFNy (both at 10 ng/ml) or media only. Cells were harvested after 24 hours or supernatant was collected after 3 days. For adoptive transfer experiments, recipient mice were given OVA (Sigma, Grade V) via the trachea one day before adoptive transfer of 10^6^ Th1 or Th2 cells. For intra-tracheal (i.t.) inoculation, mice were anaesthetized with ketamine and medetomidine and given 20 µl of OVA (10 µg) in PBS directly into the trachea. Recipient mice were given OVA i.t. on day 1 and day 3-post transfer before analysis on day 4. In some experiments, cells were isolated from recipient mice, transfected as above, and either adoptively transferred with newly generated Th1 or Th2 cells into a second recipient or used in proliferation/suppression. For 2^nd^ adoptive transfer experiments, 10^6^ newly generated Th1 or Th2 cells were co-transferred with 10^6^ isolated and transfected Treg cells from recipient mice. Twenty-four hours after the OVA i.t., mice were anaesthetized with pentobarbital. The trachea was cannulated and airspaces lavaged with 500 µl of sterile PBS for cellular analysis. For histopathological analysis lungs were removed, formalin (4% paraformaldehyde in PBS) fixed embedded in paraffin and stained with Hematoxylin and eosin (H&E). Inflammation was scored on an arbitrary 1–4+ basis taking into account both the degree of inflammation and its distribution. Local lymph nodes were isolated, prepared into a single cell suspension and cultured with OVA (10 µg/ml) for 3 days.

### ELISA

Cytokines were measured by ELISA using suppliers' guidelines. Capture and biotinylated detection antibodies for IL-4, IL-5, IL-10, IFNγ, IL-17A and IL-9 were from R&D Systems. The concentration of analytes in the sample was determined from a serial-fold diluted standard curve with OD read at 405 nm in an ELISA reader.

## Supporting Information

Figure S1 **Number and direction of common regulated genes in Lm- and Sm-Foxp3^+^ cells.** 185 common genes that were differentially regulated within Lm- Foxp3^+^ cells and Sm- Foxp3^+^ cells, indicating up or down regulated expression.
(TIFF)

Figure S2 **Isolation and transfection of primary Foxp3+ cells.** Foxp3^+^ cells were isolated from the spleen or inflamed tissue, as indicated, made into single cell suspensions (**A**), stained and FACS sorted (**B**). Purified Foxp3 cells were transfected at 2×10^5^ cells per well with miRNA mimics or inhibitors with BlockiT or SiGlo transfection indicators (**C**).
(TIFF)

Figure S3 **Th1-Treg cells potently suppress Th1 and Th2 cells in-vitro, while Th2-Treg cells only suppress Th2 cells.** Th2 or Th1 T effector (Teff, CD4^+^CD44^+^Foxp3^gfp−^) and Treg (CD4^+^Foxp3^gfp+^) cells were isolated from the lungs of recipient mice, as in Figure 4. Teff cells (10^4^) were labeled with cell trace violet and cultured alone, or in the indicated ratios with Th1-Treg or Th2-Treg cells for 3 days (**A–D**). One of 2 individual experiments shown. Freshly isolated Th1-Treg cells were transfected with miR-10a mimics or Th2-Treg cells were transfected with miR-182 inhibitors (**E**), as indicted. RNA was extracted after 24 hours and miRNA levels were quantified by RT-PCR.
(TIFF)

Figure S4 **Treg cells from naïve mice cannot suppress pathogenic Th1 or Th2 Teff cells.** Th1 (A) and Th2 (B) T effector (Teff, CD4^+^CD44^+^Foxp3^gfp−^) cells were isolated from the lungs of recipient mice, as in Figure 4 and Figure 5. As a control, naïve T cells (C) were also isolated from the spleen of OTII mice. Naïve Treg cells were isolated from naive mice. Teff or naïve T cells (10^4^) were labeled with cell trace violet (Invitrogen) and cultured alone, or in equal ratios (1∶1) with mock transfected Treg cells (B), Treg cells transfected with miR-10a mimics or Treg cells transfected with miR-182 inhibitors, as indicated, for 3 days with irradiated splenocytes (2×10^5^) and OVA (10 µg/ml). One of 2 individual experiments shown, with technical replicates shown in the scatter plot.
(TIFF)

Figure S5 **IL-4 regulates cMaf and miR-182, while IL-12/IFNγ regulate** ***Creb*** **and miR-10a expression in nTreg and iTreg cells.** In silco predicted Transcription factor binding within the promoter of miR-182 and miR-10a using PWMSCAN and TRANSFACv10.2. (**A**). FACS purified ex vivo nTreg or in vitro generated and FACS purified iTreg cells (**B**) were stimulated with IL-4 (10 ng/ml) or IL-12 (10 ng/ml)/IFNγ (10 ng/ml) for 24 hours before RNA was extracted, and mRNA (**C, D**) or miRNA (**E, F**) transcript abundance was determined by RT-PCR. One of 2 individual experiments shown. * p-value<0.05 with data expressed as mean ±SEM.
(TIFF)

Figure S6 **nTreg or in vitro generated iTreg cells from Foxp3^rfp^ and Foxp3^gfp^ mice do not differ in miR-182 or miR-10 expression.** Ex vivo isolated nTreg (**A**) and in vitro generated iTreg cells (**B**) were FACS purified from Foxp3^gfp^ or Foxp3^rfp^ reporter mice. RNA was immediately extracted and miR-182 and mIR-10a levels were determined by RT-PCR, and expressed relative to Foxp3^−^ cells with data expressed as mean ±SEM.
(TIFF)

Figure S7 **IL-4-regulated miR-182 and IL-12/IFNγ-regulated miR-10a control IL-2 and IFNγ production, respectively.** FACS purified ex vivo nTreg or in vitro generated and FACS purified iTreg cells were stimulated with IL-4 (10 ng/ml) or IL-12 (10 ng/ml)/IFNγ (10 ng/ml) for 24 hours before RNA was extracted and mRNA (**A–E**) transcript abundance determined by RT-PCR. FACS purified nTreg and iTreg cells were transfected with miR-182 inhibitors (**E**) or miR-10a mimics (**F**) before treatment with IL-4 or IL-12/IFNγ. Cells were recovered after 24 hours for mRNA analysis or supernatants were recovered after 3 days of culture for protein analysis (**E**). One of 2 individual experiments shown. * p-value<0.05 with data expressed as mean ±SEM.
(TIFF)

Figure S8 **Adoptive transfer system.** One million Th1 or Th2 polarised cells from C57BL/6 CD45.1 OTII RAG2^−/−^ mice were adoptively transferred into CD45.2 Foxp3^gfp^ mice (Recipient 1) one day after i.t. OVA treatment. Recipient Mice were given 2 additional OVA treatments 1 and 3 days post transfer. CD4^+^Foxp3^gfp^ cells were isolated from the lungs of recipient mice and either untreated, Mock transfected or transfected with miRNA mimics or inhibitors (as in Figure S2). Fresh Th1 or Th2 polarised cells from C57BL/6 CD45.1 OTII RAG2^−/−^ mice were generated and co-transferred with the treated CD4^+^Foxp3^gfp^ cells into a third mouse (Recipient 2), one day after OVA challenge. Recipient 2 mice with treated with OVA i.t. 1 and 3 days post transfer and were anlaysed on day 4 post transfer.
(TIFF)

Table S1 **Significantly differentially regulated genes in Foxp3^+^ populations represented in Heat map (left table) and in samples, as indicated (right 3 tables).**
(PDF)

Table S2 **Mapping of Deep Sequencing reads.** Mapping of data to SOLiD dataset, miRBase and the mouse genome. (**A**) Uniquely mapped reads, (**B**) All mapped reads, (**C**) Representation of other RNA species in dataset. Representative workflow of mapping strategy. Sm = *Schistosoma mansoni*-derived Treg. Lm = *Leishmania major*-derived Treg.
(PDF)

Table S3 **Significantly differentially regulated miRNAs in Foxp3^+^ populations.** Fold change of significantly regulated miRNAs.
(PDF)

Table S4 **Candidate master regulatory miRNAs identified from Monte Carlo simulation.**
(PDF)

Table S5 **Predicted mRNA targets of miR-182 and miR-10a identified from Monte Carlo analysis.**
(PDF)
