# Supplementary figures and images for: miR-182 and miR-10a Are Key Regulators of Treg Specialisation and Stability during Schistosome and Leishmania-associated Inflammation
Source: PLoS Pathog. 2013 Jun 27;9(6):e1003451. doi: 10.1371/journal.ppat.1003451 (PMC3695057; doi:10.1371/journal.ppat.1003451)

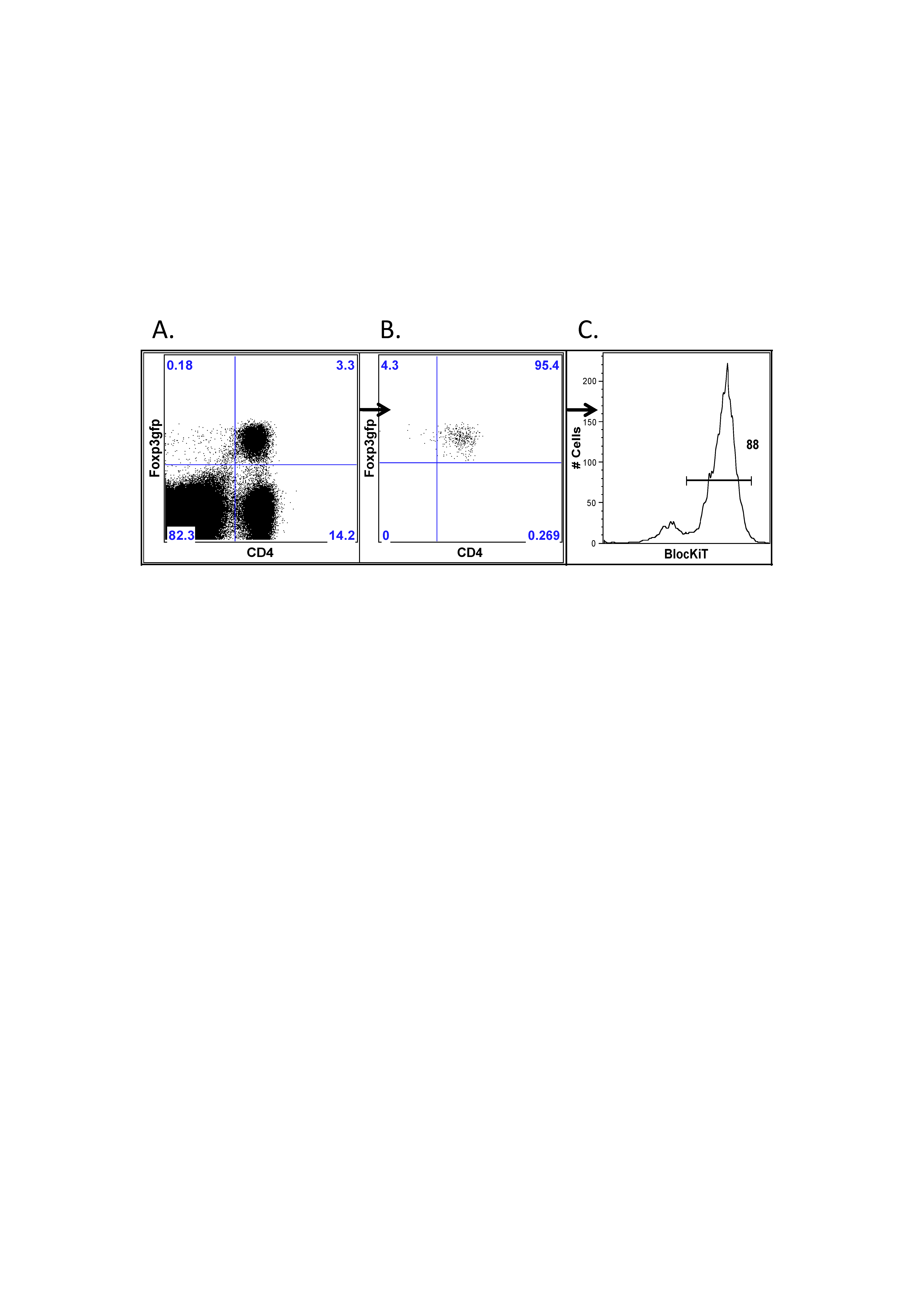

Supplement: Figure S2 — Isolation and transfection of primary Foxp3+ cells. Foxp3+ cells were isolated from the spleen or inflamed tissue, as indicated, made into single cell suspensions (A), stained and FACS sorted (B). Purified Foxp3 cells were transfected at 2×10^5^ cells per well with miRNA mimics or inhibitors with BlockiT or SiGlo transfection indicators (C). (TIFF) [file ppat.1003451.s002.tif]

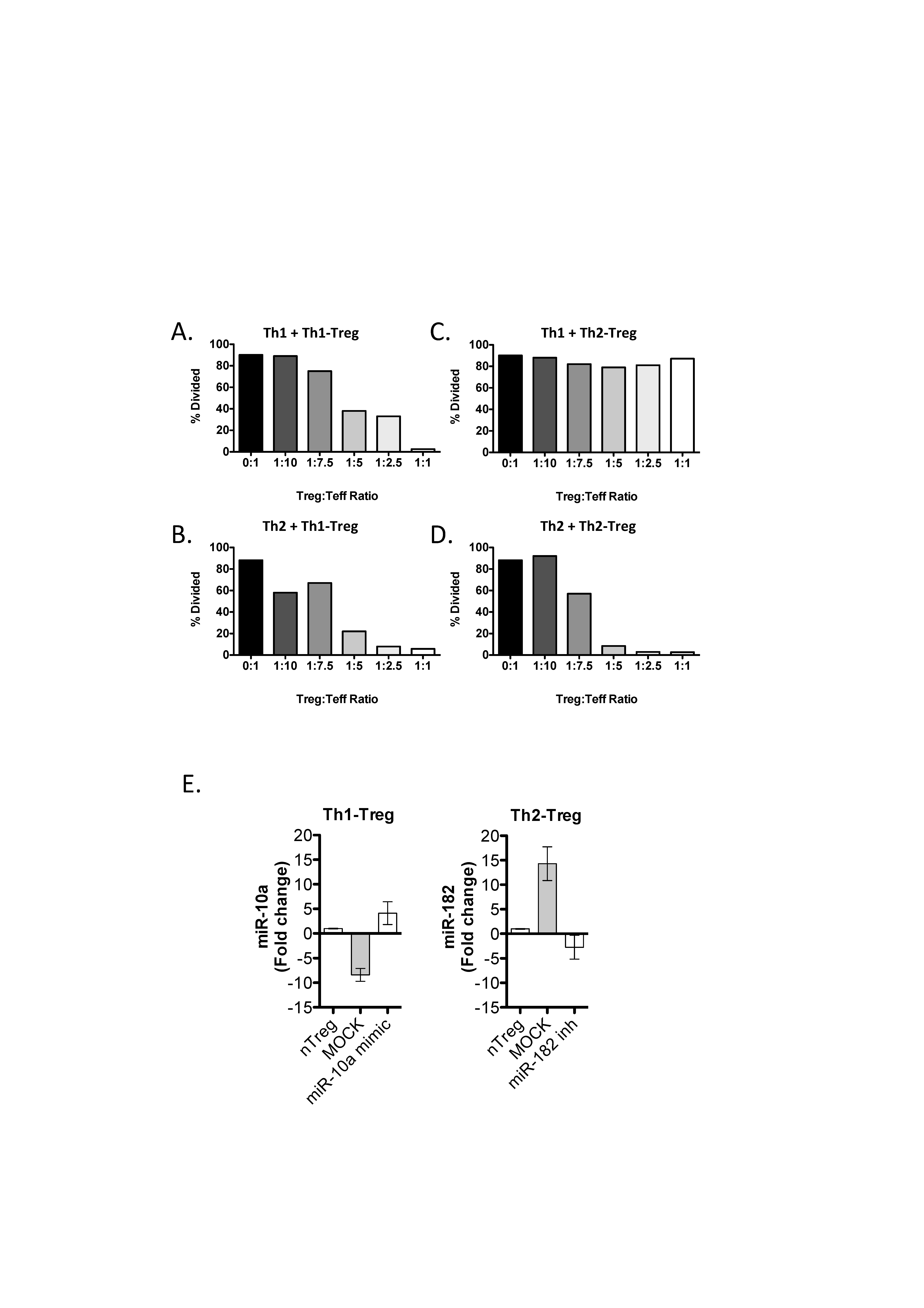

Supplement: Figure S3 — Th1-Treg cells potently suppress Th1 and Th2 cells in-vitro, while Th2-Treg cells only suppress Th2 cells. Th2 or Th1 T effector (Teff, CD4+CD44+Foxp3gfp−) and Treg (CD4+Foxp3gfp+) cells were isolated from the lungs of recipient mice, as in Figure 4. Teff cells (104) were labeled with cell trace violet and cultured alone, or in the indicated ratios with Th1-Treg or Th2-Treg cells for 3 days (A–D). One of 2 individual experiments shown. Freshly isolated Th1-Treg cells were transfected with miR-10a mimics or Th2-Treg cells were transfected with miR-182 inhibitors (E), as indicted. RNA was extracted after 24 hours and miRNA levels were quantified by RT-PCR. (TIFF) [file ppat.1003451.s003.tif]

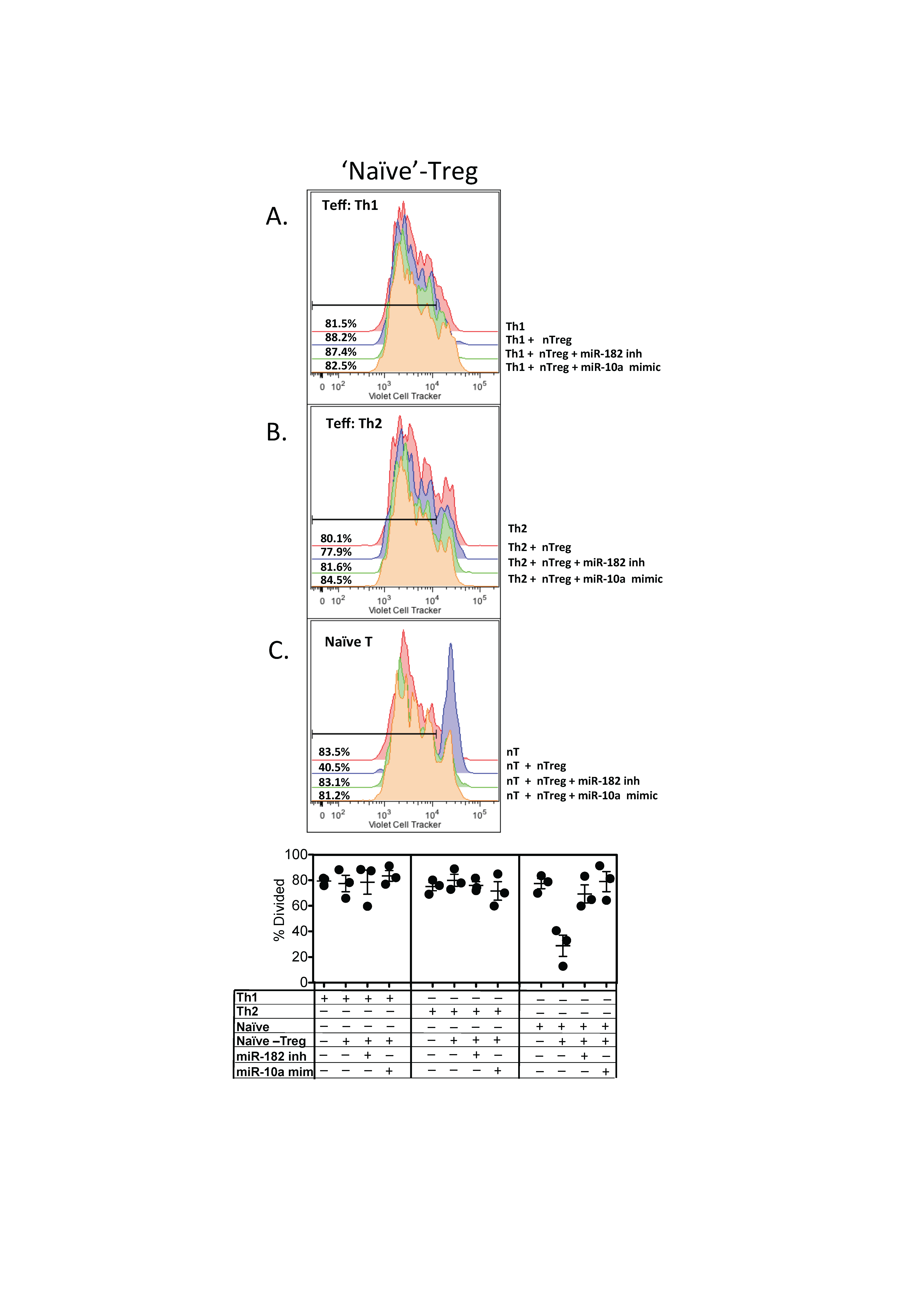

Supplement: Figure S4 — Treg cells from naïve mice cannot suppress pathogenic Th1 or Th2 Teff cells. Th1 (A) and Th2 (B) T effector (Teff, CD4+CD44+Foxp3gfp−) cells were isolated from the lungs of recipient mice, as in Figure 4 and Figure 5. As a control, naïve T cells (C) were also isolated from the spleen of OTII mice. Naïve Treg cells were isolated from naive mice. Teff or naïve T cells (104) were labeled with cell trace violet (Invitrogen) and cultured alone, or in equal ratios (1∶1) with mock transfected Treg cells (B), Treg cells transfected with miR-10a mimics or Treg cells transfected with miR-182 inhibitors, as indicated, for 3 days with irradiated splenocytes (2×10^5^) and OVA (10 µg/ml). One of 2 individual experiments shown, with technical replicates shown in the scatter plot. (TIFF) [file ppat.1003451.s004.tif]

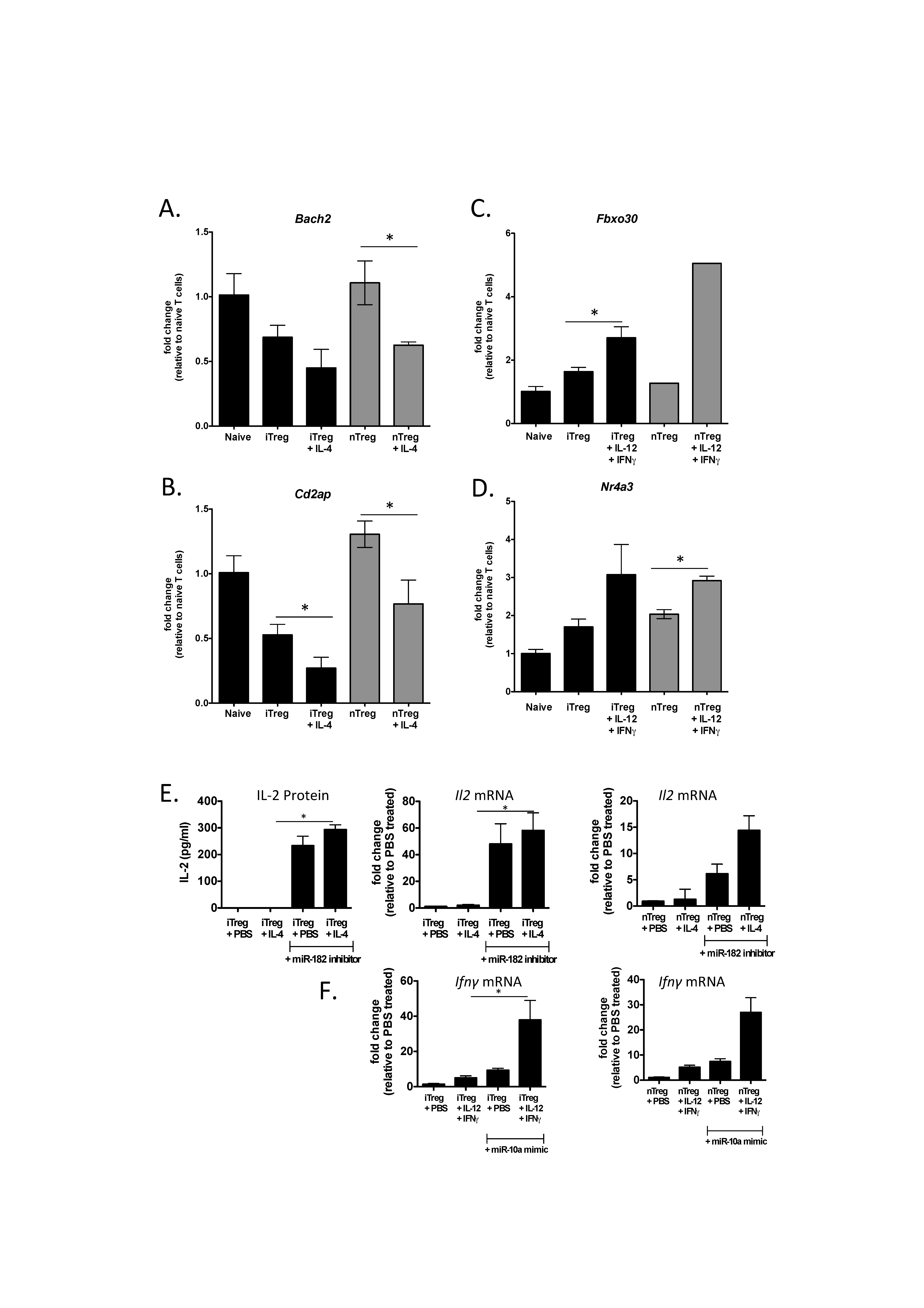

Supplement: Figure S7 — IL-4-regulated miR-182 and IL-12/IFNγ-regulated miR-10a control IL-2 and IFNγ production, respectively. FACS purified ex vivo nTreg or in vitro generated and FACS purified iTreg cells were stimulated with IL-4 (10 ng/ml) or IL-12 (10 ng/ml)/IFNγ (10 ng/ml) for 24 hours before RNA was extracted and mRNA (A–E) transcript abundance determined by RT-PCR. FACS purified nTreg and iTreg cells were transfected with miR-182 inhibitors (E) or miR-10a mimics (F) before treatment with IL-4 or IL-12/IFNγ. Cells were recovered after 24 hours for mRNA analysis or supernatants were recovered after 3 days of culture for protein analysis (E). One of 2 individual experiments shown. * p-value<0.05 with data expressed as mean ±SEM. (TIFF) [file ppat.1003451.s007.tif]

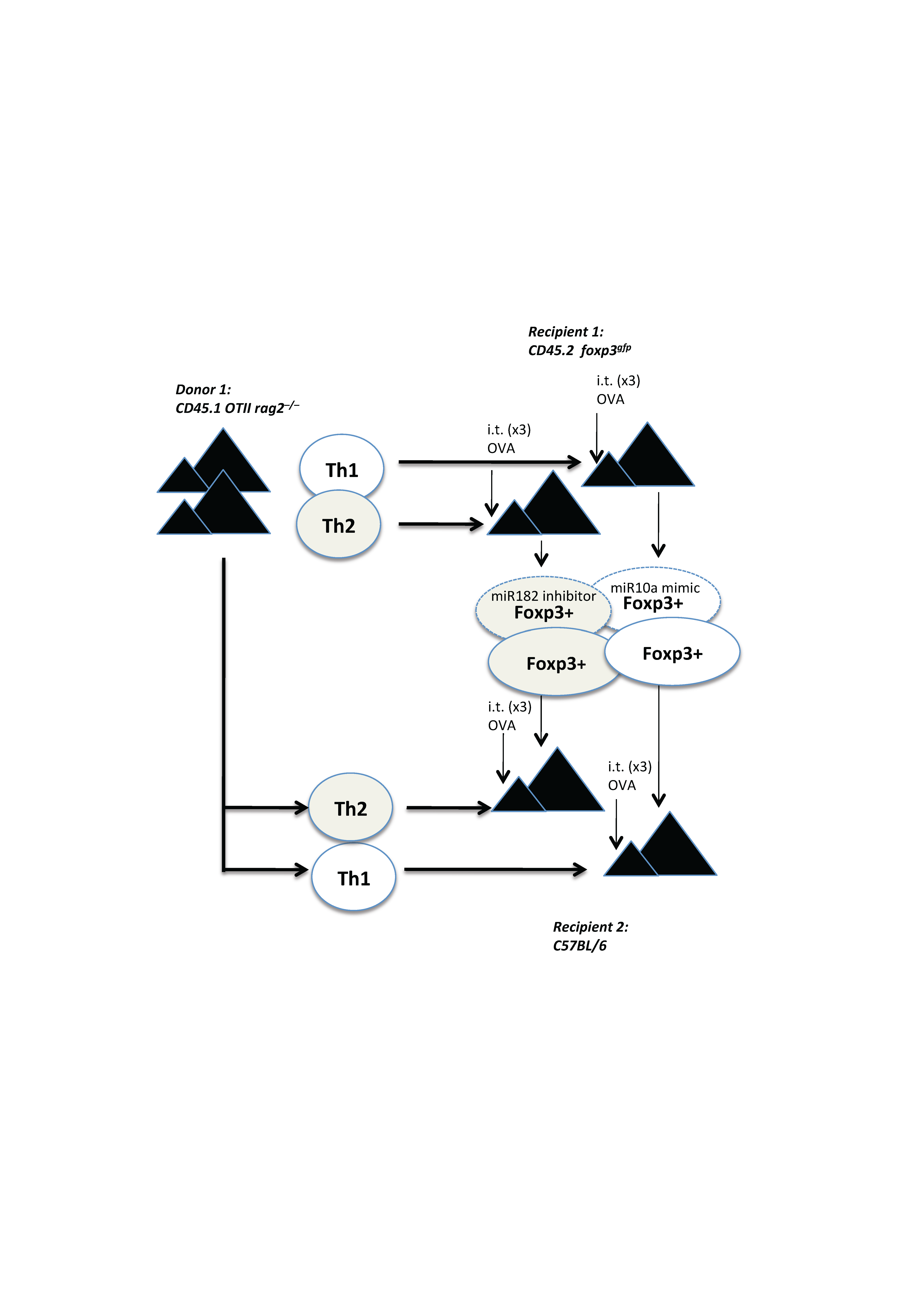

Supplement: Figure S8 — Adoptive transfer system. One million Th1 or Th2 polarised cells from C57BL/6 CD45.1 OTII RAG2−/− mice were adoptively transferred into CD45.2 Foxp3gfp mice (Recipient 1) one day after i.t. OVA treatment. Recipient Mice were given 2 additional OVA treatments 1 and 3 days post transfer. CD4+Foxp3gfp cells were isolated from the lungs of recipient mice and either untreated, Mock transfected or transfected with miRNA mimics or inhibitors (as in Figure S2). Fresh Th1 or Th2 polarised cells from C57BL/6 CD45.1 OTII RAG2−/− mice were generated and co-transferred with the treated CD4+Foxp3gfp cells into a third mouse (Recipient 2), one day after OVA challenge. Recipient 2 mice with treated with OVA i.t. 1 and 3 days post transfer and were anlaysed on day 4 post transfer. (TIFF) [file ppat.1003451.s008.tif]
