# Supplementary material for: miR-182 and miR-10a Are Key Regulators of Treg Specialisation and Stability during Schistosome and Leishmania-associated Inflammation
Source: PLoS Pathog. 2013 Jun 27;9(6):e1003451. doi: 10.1371/journal.ppat.1003451 (PMC3695057; doi:10.1371/journal.ppat.1003451)
Supplement: Table S1 — Significantly differentially regulated genes in Foxp3+ populations represented in Heat map (left table) and in samples, as indicated (right 3 tables). (PDF) [file ppat.1003451.s009.pdf]

Significantly expressed array probes

Transcript ID	Gene Symbol	RefSeq	p-value
10412211	Gzma	NM_010370	5.68E-09
10402325	Asb2	NM_023049	1.45E-08
10405785	0610007P08Rik	NM_023507	1.66E-08
10546855	Srgap3	NM_080448	1.89E-08
10384458	Plek	NM_019549	1.96E-08
10349603	Il10	NM_010548	2.23E-08
10545921	Mxd1	NM_010751	2.35E-08
10513145	Ptpn3	NM_011207	2.38E-08
10354418	Obfc2a	NM_028696	2.53E-08
10378286	Itgae	NM_008399	3.16E-08
10550509	Pglyrp1	NM_009402	4.31E-08
10413928	1810011H11Rik	AK007434	5.76E-08
10346843	Nrp2	NM_001077403	6.91E-08
10519983	Fgl2	NM_008013	8.53E-08
10539135	Capg	NM_007599	1.11E-07
10535065	Adap1	NM_172723	1.20E-07
10525591	Kntc1	NM_001042421	1.20E-07
10398039	Serpina3f	NM_001033335	1.23E-07
10598013	Ccr5	NM_009917	1.36E-07
10590635	Ccr5	NM_009917	1.36E-07
10357345	E030049G20Rik	NM_172484	1.51E-07
10546853	Srgap3	NM_080448	1.81E-07
10540897	Pparg	NM_001127330	1.85E-07
10455784	Gramd3	NM_026240	2.17E-07
10511282	Tnfrsf4	NM_011659	2.63E-07
10415844	Ctsb	NM_007798	3.14E-07
10421517	Cysltr2	NM_133720	3.48E-07
10375145	Lcp2	NM_010696	3.89E-07
10414817	Tcra	U07662	3.95E-07
10389064	Ccl1	NM_011329	4.00E-07
10471555	Angptl2	NM_011923	4.08E-07
10574532	Ces2	NM_145603	4.36E-07
10592847	Myl6	NM_010860	4.39E-07
10408850	Nedd9	NM_001111324	5.15E-07
10513141	Ptpn3	NM_011207	5.16E-07
10542006	D6Wsu163e	BC016429	5.43E-07
10521678	Cd38	NM_007646	5.46E-07
10420308	Gzmb	NM_013542	5.49E-07
10473125	Itga4	NM_010576	5.64E-07
10533729	Vps37b	NM_177876	5.96E-07
10350102	Ptpn7	NM_177081	6.05E-07
10593050	Il10ra	NM_008348	6.44E-07
10477187	Tpx2	NM_001141977	6.53E-07
10494001	Tdpoz4	NM_207272	6.63E-07
10487508		---	6.85E-07
10523182	Areg	NM_009704	7.06E-07
10571975		---	7.21E-07
10353420	Mcm3	NM_008563	7.27E-07
10488237	Snx5	NM_024225	7.51E-07
10479833	Optn	NM_181848	7.52E-07
10562000	Psenen	NM_025498	7.63E-07
10342896		---	7.78E-07
10534456	Hip1	NM_146001	7.86E-07
10508182	Psmb2	NM_011970	8.52E-07
10365426	Psma5	NM_011967	1.03E-06
10467852	Slc25a28	NM_145156	1.05E-06
10354372	Myl6	NM_010860	1.07E-06
10504159	Ccl19	NM_011888	1.14E-06
10512322	Ccl19	NM_011888	1.14E-06
10578557	Ccdc111	NM_001001184	1.17E-06
10390328	Tbx21	NM_019507	1.35E-06
10409240	Sema4d	NM_013660	1.41E-06
10382797	Fam100b	NM_176902	1.43E-06
10503399	Myl6	NM_010860	1.50E-06
10515836	Ccnb1	NM_172301	1.58E-06
10429926	Dgat1	NM_010046	1.60E-06
10541482	Nhp2l1	NM_011482	1.61E-06
10555063	Ints4	NM_027256	1.66E-06
10512372	Ccl19	NM_011888	1.66E-06
10527936	Fzd1	NM_021457	1.69E-06
10592515	Ubash3b	NM_176860	1.84E-06
10476945	Cst7	NM_009977	1.85E-06
10562192	Fxyd5	NM_008761	1.91E-06
10339165		---	1.97E-06
10420011	Myl6	NM_010860	2.01E-06
10376208	Gm2a	NM_010299	2.08E-06
10605674	Pola1	NM_008892	2.10E-06
10469151	Itih5	NM_172471	2.16E-06
10429564	Ly6a	NM_010738	2.19E-06
10402800	Gpr132	NM_019925	2.29E-06
10575777	4933407C03Rik	BC158118	2.34E-06
10568714	Mki67	NM_001081117	2.38E-06
10391931	Lrrc37a	XM_137868	2.42E-06
10434467	Psmd2	NR_027485	2.47E-06
10512125	Psenen	NM_025498	2.53E-06
10552406	Nkg7	NM_024253	2.55E-06
10407097	Pde4d	NM_011056	2.70E-06
10590242	Ccr8	NM_007720	2.74E-06
10504188	Ccl19	NM_011888	2.79E-06
10586227	Dennd4a	NM_001162917	2.87E-06
10362615	Traf3ip2	NM_134000	2.95E-06
10491081		---	2.97E-06
10452633	Tgif1	NM_009372	3.06E-06
10568792	Stk32c	NM_021302	3.10E-06
10586246	Dennd4a	NM_001162917	3.15E-06
10381172	Stat5a	NM_011488	3.29E-06
10373396	Myl6	NM_010860	3.34E-06
10462363	Jak2	NM_008413	3.38E-06
10501629	Cdc14a	NM_001080818	3.52E-06
10409278	Nfil3	NM_017373	3.55E-06
10346970	Pikfyve	NM_011086	3.72E-06
10440406	Nrip1	NM_173440	3.77E-06
10460102	1700034H14Rik	BC034297	3.79E-06
10462796	Kif11	NM_010615	3.89E-06
10417561	Fam107a	NM_183187	4.02E-06
10591816	Dpy19l1	NM_172920	4.20E-06
10533182	Dtx1	NM_008052	4.32E-06
10486875	Frmd5	NM_172673	4.42E-06
10343098		---	4.43E-06
10521913	Rbpj	NM_009035	4.60E-06
10496204	Cenpe	NM_173762	4.72E-06
10444800	Nhp2l1	NM_011482	4.79E-06
10488090	Tasp1	NM_175225	4.80E-06
10531724	Plac8	NM_139198	4.81E-06
10485687	Mett5d1	NM_029790	4.92E-06

S. mansoni-derived Foxp3$^+$

gene symbol	Fold Change	p-value
Gp49a	10.98	8.71E-10
Lilrb4	9.00	6.91E-08
Klrg1	4.90	2.46E-07
Gm14005	4.40	3.31E-08
Plac8	4.24	7.68E-06
Cenpk	4.14	2.11E-06
Neb	3.98	6.98E-08
1810011H11Rik	3.94	4.69E-07
Gzma	3.88	3.50E-08
Cenph	3.73	8.89E-05
Arl5a	3.68	2.52E-09
Nuf2	3.67	5.08E-06
Hmmr	3.65	1.23E-06
Nkg7	3.52	1.76E-07
Serpina3f	3.48	1.75E-08
Plek	3.46	4.81E-08
Tdpoz4	3.23	3.21E-07
Prr11	3.22	7.06E-06
Ncapg	3.21	1.04E-06
Tpx2	3.18	2.72E-06
Casc5	3.17	6.05E-05
Bub1	3.15	1.11E-04
Lag3	3.10	1.73E-06
Cenpe	3.09	7.63E-06
Ccl5	3.07	1.76E-06
Prelid2	3.05	5.55E-07
Gm8721	3.00	3.68E-05
Kif4	2.97	4.28E-05
Ifitm3	2.80	7.67E-06
Nusap1	2.80	2.52E-05
Srgap3	2.75	1.00E-07
Depdc1a	2.75	2.23E-05
Cenpf	2.69	8.96E-06
Shcbp1	2.69	1.50E-03
Dstn	2.68	3.72E-05
Tbx21	2.65	7.57E-09
Rbpj	2.63	7.73E-11
Ccnb2	2.61	5.31E-06
Ttk	2.59	4.27E-05
Ncapg2	2.59	8.99E-06
C79407	2.59	7.57E-05
Alcam	2.58	1.36E-06
Snx9	2.58	4.45E-08
Ccna2	2.58	6.08E-05
Epcam	2.56	5.22E-07
Tnfsf10	2.56	7.97E-06
Kif15	2.55	8.27E-05
Hells	2.55	8.99E-06
Arhgap11a	2.54	1.56E-06
Spc25	2.53	1.81E-04
Ctsw	2.52	7.47E-08
2810417H13Rik	2.52	3.95E-04
N4bp1	2.51	1.66E-05
Nckap5	2.51	6.18E-07
AW112010	2.47	1.94E-06
Cks1b	2.46	2.86E-04
E2f8	2.44	9.39E-05
Cxcr6	2.40	2.17E-05
Ckap2	2.39	3.78E-05
Nckap5	2.36	2.73E-06
Aspm	2.34	2.95E-05
Pparg	2.34	2.70E-07
Lgals1	2.33	4.94E-07
Gsg2	2.30	1.28E-03
Ccl1	2.30	1.60E-07
Ccdc46	2.30	3.96E-07
Esco2	2.29	4.24E-06
Sgol2	2.28	6.85E-04
Kntc1	2.27	3.53E-07
Stil	2.27	3.21E-05
LOC100504914	2.27	1.84E-07
Cdca8	2.26	4.93E-05
Neil3	2.24	2.17E-04
Chek1	2.24	7.36E-06
Cxcl2	2.24	1.62E-03
Lztfl1	2.24	2.06E-04
Ly6a	2.24	4.71E-10
Entpd1	2.22	7.59E-06
Bub1b	2.21	1.01E-04
Nampt	2.19	1.57E-03
Csf1	2.19	5.06E-06
Sema7a	2.19	6.52E-06
Glrx	2.18	1.02E-05
Rrm1	2.17	2.95E-05
Gm2a	2.17	4.87E-09
Pglyrp1	2.16	4.00E-08
Birc5	2.16	3.80E-04
Tacc3	2.16	3.43E-04
Dscc1	2.15	2.00E-05
Casp3	2.14	1.62E-06
Hip1	2.14	5.84E-08
Mapre2	2.13	7.41E-05
Fmnl2	2.13	2.48E-07
Rad51	2.12	9.01E-05
Mastl	2.12	1.14E-05
Rrm2	2.11	9.70E-05
Kif14	2.09	1.08E-04
Itga1	2.08	1.04E-05
Fabp5	2.08	3.09E-06
Cdk6	2.07	3.06E-06
Cd200r1	2.07	1.05E-05
Hist1h4f	2.06	5.08E-03
Anxa2	2.05	1.11E-07
Anln	2.05	6.30E-04
Cdkn3	2.05	1.54E-04
Capg	2.04	2.59E-07
Tigit	2.04	3.92E-07
Anxa4	2.03	1.40E-05
Srxn1	2.03	2.22E-06
Cxcr3	2.03	8.92E-06
Gins1	2.02	2.91E-05
Nedd4	2.01	1.47E-07
Actr3b	1.99	7.61E-05
Ncaph	1.99	1.97E-04
Gda	1.99	6.66E-05

Common genes

gene symbol	S. mansoni-derived Foxp3$^+$		L. major-derived Foxp3$^+$	
	Fold Change	p-value	Fold Change	p-value
Gzmb	11.81	1.19E-08	6.49	1.87E-04
Ccr5	8.92	5.55E-10	5.39	5.79E-05
Il10	7.98	8.80E-08	6.67	1.19E-06
Ccr2	5.49	3.11E-08	5.11	6.72E-04
Kif11	4.79	2.27E-06	1.60	5.80E-03
Snord118	4.53	2.10E-03	4.74	1.26E-02
Ccr8	4.42	5.44E-06	3.92	7.90E-05
Il1rl1	4.41	1.37E-08	4.31	1.11E-03
Havcr2	3.86	2.04E-07	5.06	2.25E-04
Hist1h2bl	3.86	1.24E-05	2.85	7.73E-03
Id2	3.84	6.36E-07	2.30	7.84E-03
Hist1h2bk	3.80	1.03E-05	2.72	6.14E-03
Stmn1	3.80	1.19E-05	2.71	3.26E-04
Prdm1	3.79	4.87E-09	2.46	3.55E-03
Mki67	3.71	1.96E-06	1.57	2.55E-03
Fgl2	3.71	8.08E-10	1.56	3.94E-03
Areg	3.71	7.74E-07	3.77	1.20E-05
Itgae	3.60	2.61E-09	2.45	3.69E-05
Hist1h2bf	3.50	1.49E-05	2.60	8.61E-03
Hist1h2bj	3.49	1.49E-05	2.54	6.80E-03
Top2a	3.09	4.91E-05	1.76	9.27E-03
Cysltr2	2.99	1.70E-06	2.08	1.94E-04
Ccl4	2.89	2.55E-05	2.11	1.33E-03
Ctla2a	2.85	1.48E-05	3.61	1.66E-03
Ccnb1	2.80	6.37E-06	1.69	2.81E-03
Insm1	2.79	1.24E-03	6.55	1.31E-04
Egln3	2.75	6.55E-07	2.33	1.02E-02
C330027C09Ri	2.70	7.80E-05	1.65	7.10E-03
Hist1h2bn	2.68	9.17E-04	2.38	1.55E-02
Hist1h2ao	2.58	7.25E-05	1.77	2.37E-03
Cks2	2.55	7.16E-06	2.46	2.43E-04
Cep55	2.53	5.58E-05	1.59	1.56E-02
Gm5486	2.48	3.85E-08	2.47	1.87E-03
Prc1	2.48	6.47E-06	1.62	3.60E-03
Mt2	2.35	6.28E-07	2.45	9.10E-03
Ell2	2.35	1.52E-06	3.29	1.24E-04
Sv2c	2.34	2.02E-04	2.15	1.59E-02
Ttc39c	2.30	6.40E-06	2.86	6.56E-03
Tpi1	2.25	4.49E-07	2.21	9.27E-03
Lmnb1	2.24	7.07E-06	2.15	1.15E-04
Brip1	2.23	6.48E-06	1.51	4.31E-03
Clcn5	2.22	2.64E-06	1.84	7.91E-03
Atp2b4	2.20	1.43E-08	2.13	1.30E-02
Kif23	2.20	5.67E-05	2.09	1.11E-03
Gem	2.18	5.43E-03	4.17	9.78E-10
Hist1h2ai	2.16	1.21E-04	1.70	4.96E-03
Hist1h2an	2.16	1.44E-04	1.84	3.78E-03
Myo1d	2.16	4.67E-05	1.61	7.37E-03
Hist1h2af	2.14	1.32E-04	1.82	3.40E-03
Lamc1	2.13	1.47E-06	2.14	1.94E-03
Hist1h2ah	2.12	1.30E-04	1.81	2.71E-03
Rgs16	2.10	2.54E-04	1.54	2.10E-03
Epas1	2.08	7.32E-07	1.57	9.56E-04
Mt1	2.07	6.70E-05	2.43	3.63E-05
Ccr3	2.06	1.69E-04	1.62	9.07E-03
Cd74	2.06	1.47E-05	2.25	1.40E-04
Cpd	2.05	2.13E-06	1.85	1.21E-02
Hist1h2ak	2.05	3.74E-05	1.91	4.52E-03
Gas2l3	2.03	4.19E-07	2.44	2.95E-03
Tnfrsf4	2.03	8.76E-08	1.64	1.53E-04
Odc1	2.02	3.15E-04	2.56	4.90E-04
Tnfrsf9	2.02	4.18E-06	2.34	2.65E-03
Cdc25c	1.99	6.65E-04	1.53	7.95E-05
Ptpn3	1.98	7.34E-05	-1.56	1.35E-03
Gadd45b	1.97	2.65E-04	1.85	1.48E-03
Nr4a2	1.97	2.00E-03	2.46	8.78E-07
Nr4a3	1.96	2.64E-04	2.27	1.04E-03
Jun	1.94	4.26E-03	2.14	7.08E-04
Dusp16	1.93	2.32E-05	1.65	2.44E-03
Nfil3	1.91	4.53E-06	2.43	3.37E-05
Myadm	1.91	7.92E-07	1.89	4.60E-04
Dgat1	1.89	3.38E-06	4.45	1.56E-05
Ero1l	1.85	3.43E-07	3.43	1.21E-03
Nr4a1	1.83	3.11E-04	2.11	4.62E-06
Rbbp8	1.83	1.07E-04	1.97	8.09E-04
Pgk1	1.83	7.68E-07	1.62	1.63E-02
Bhlhe40	1.80	3.83E-03	2.89	2.01E-04
Itgav	1.80	4.68E-06	2.02	5.02E-03
Lyn	1.78	8.21E-07	1.50	1.66E-03
Slc15a3	1.77	3.68E-06	1.85	5.08E-05
Gm10291	1.75	1.84E-05	1.83	4.54E-03
Chd7	1.75	1.95E-03	1.74	6.87E-03
Tnfrsf1b	1.74	1.30E-05	1.52	1.91E-03
Dennd4a	1.72	3.49E-03	4.32	6.31E-05
Cst7	1.71	5.84E-06	-1.65	7.06E-04
Nhp2l1	1.71	1.03E-04	2.00	1.41E-05
Runx2	1.71	1.39E-04	2.27	5.52E-05
Hist1h3h	1.71	5.29E-04	1.57	5.75E-03
Hist1h3d	1.70	2.05E-04	1.55	6.96E-03
Samsn1	1.70	1.24E-07	1.64	1.09E-03
Apaf1	1.70	1.79E-06	1.82	1.26E-02
Scpep1	1.69	1.35E-03	1.71	4.63E-03
Aldoa	1.69	3.12E-06	1.67	6.19E-03
Cish	1.69	4.43E-05	1.50	5.47E-03
Hist2h2aa1	1.68	2.86E-04	1.53	3.42E-03
Hist1h3b	1.68	2.96E-04	1.53	9.59E-03
Mreg	1.66	1.10E-04	2.13	1.60E-02
Gm5177	1.66	1.05E-05	1.66	1.42E-03
H2-Ab1	1.65	2.82E-05	1.81	9.91E-03
Hsf2	1.65	7.89E-04	1.50	1.15E-02
Gm2451	1.65	1.00E-05	1.81	3.08E-03
Runx2	1.64	1.30E-05	1.79	7.73E-04
Vim	1.64	4.88E-07	1.63	5.10E-03
Hist1h3i	1.64	1.36E-04	1.52	9.78E-03
Hist1h2bm	1.63	2.77E-04	1.69	1.24E-03
Cdkn1a	1.63	1.88E-04	3.34	5.00E-04
Hist2h3b	1.62	3.33E-04	1.51	9.32E-03
Got1	1.62	2.32E-03	3.96	2.91E-05
Atp8a2	1.61	1.14E-05	1.57	8.18E-05
Uhrf1	1.60	3.95E-05	1.53	1.36E-03
Fam46a	1.59	1.16E-03	1.89	1.63E-02
Hist1h3c	1.59	5.36E-04	1.51	8.94E-03
Tnfrsf18	1.57	4.19E-04	1.59	6.44E-03
Hist1h3e	1.57	3.23E-04	1.52	5.91E-03
Egr2	1.57	9.00E-05	-1.57	1.70E-03
Icos	1.57	3.15E-05	2.28	7.35E-05

L. major-derived Foxp3$^+$

gene symbol	Fold change	p-value
Hspa1a	12.12	3.10E-10
Vps37b	4.54	8.99E-06
Hist1h1c	3.54	2.50E-04
Hsph1	3.42	9.36E-03
Snord14c	3.19	6.11E-06
Snord52	3.18	7.21E-04
Gcnt1	3.11	1.52E-04
Rgs2	3.05	9.20E-08
Ifrd1	3.02	5.53E-06
Dusp4	2.99	9.90E-07
Mxi1	2.89	1.62E-03
Mir16-1	2.85	1.71E-02
Fosb	2.77	6.56E-07
Fosl2	2.77	1.28E-05
Errfi1	2.72	2.89E-05
Coq10b	2.68	4.89E-04
Atf3	2.66	1.19E-06
7330403C04Rik	2.57	4.34E-05
Snord14e	2.52	2.45E-04
Gla	2.50	4.60E-05
Slc3a2	2.48	8.21E-06
Tex2	2.44	2.00E-03
Rnf157	2.43	1.46E-04
Polg2	2.34	2.40E-05
Rasgef1b	2.33	4.55E-06
Armcx5	2.31	5.48E-03
Ets2	2.30	1.06E-05
H2-Aa	2.24	1.03E-02
Slc2a1	2.20	5.71E-04
Gm10694	2.19	1.72E-03
Gm10008	2.18	3.78E-03
B930036N10Rik	2.16	1.34E-02
Mxd1	2.15	1.27E-09
Fam100b	2.14	1.06E-05
Slc5a3	2.12	6.16E-03
Lphn2	2.11	7.69E-03
Snord38a	2.11	2.60E-03
Rhob	2.10	1.32E-03
3110043O21Rik	2.10	8.90E-05
Crem	2.09	3.11E-04
Wbp5	2.09	5.31E-03
Ctla2b	2.06	3.47E-04
Ptp4a1	2.06	2.80E-04
Rora	2.06	2.05E-04
Ppp1r15a	2.05	1.69E-03
2810026P18Rik	2.02	4.03E-04
Ramp3	2.02	5.41E-04
Zbtb10	2.02	8.14E-03
AI452195	2.02	3.08E-03
Fabp5l2	2.01	1.24E-03
Lmna	2.01	4.01E-05
Faim	2.00	2.33E-04
Tinf2	1.99	1.99E-05
Plk2	1.98	3.40E-04
P4ha1	1.96	4.06E-03
Gm13363	1.96	3.04E-05
1700047G07Rik	1.96	1.35E-03
Spty2d1	1.95	2.13E-04
Sik1	1.95	1.24E-05
Btg3	1.95	1.23E-05
Stat3	1.94	5.40E-05
Phlpp1	1.91	2.65E-03
Hk2	1.91	3.65E-03
Dnajb4	1.90	4.43E-05
Ndrg1	1.90	2.04E-03
Uap1	1.88	3.77E-05
Il23r	1.88	1.15E-02
Syne2	1.87	1.11E-02
Neurl3	1.86	2.26E-05
Zdhhc23	1.85	8.05E-03
Sh3rf1	1.85	3.39E-04
Gramd3	1.84	3.49E-06
1190002H23Rik	1.84	5.16E-04
Sparc	1.83	8.45E-03
Lmo7	1.83	6.35E-03
Tgif1	1.82	1.50E-05
Dnajb1	1.82	1.47E-03
Jak2	1.82	2.51E-05
3010003L21Rik	1.81	2.26E-03
Mir24-1	1.80	1.78E-03
Tmtc2	1.79	5.65E-03
Cxcl10	1.79	2.50E-04
Bcl6	1.79	2.18E-03
Tctex1d1	1.79	1.12E-02
Il18r1	1.77	1.12E-03
Rel	1.76	4.07E-05
Tiparp	1.76	1.55E-02
Tpd52	1.75	1.33E-03
Gm10293	1.75	4.52E-04
Arl5b	1.75	4.94E-04
Snx18	1.74	5.75E-03
Jmy	1.74	5.78E-03
Slc16a3	1.74	5.56E-03
Tnfaip3	1.74	2.11E-05
Per1	1.74	3.05E-05
Fos	1.73	4.67E-04
Gapdh	1.73	4.86E-04
Arrdc3	1.72	2.56E-05
Vgll4	1.72	7.31E-06
Clcn3	1.71	1.02E-03
Tfrc	1.71	9.56E-03
Zfand2a	1.71	1.37E-03
Furin	1.71	5.79E-04
Olfr1372-ps1	1.71	9.62E-06
Gnb4	1.71	1.12E-03
Ust	1.70	1.25E-02
Plcxd2	1.69	1.15E-03
Hsp90aa1	1.69	2.93E-03
Irf4	1.68	2.27E-05
Hspb1	1.68	9.97E-03
Homer2	1.68	6.68E-03
Krt17	1.68	1.16E-02
Emb	1.68	2.85E-06
Fbxo30	1.68	6.76E-03
Klhdc3	1.68	5.17E-03

10586933	Nedd4	NM_010890	5.19E-06
10585091	Nhp2l1	NM_011482	5.20E-06
10589030	Qrich1	NM_001114119	5.26E-06
10463070	Entpd1	NM_009848	5.31E-06
10499394	Lmna	NM_001002011	5.36E-06
10476207	Atrn	NM_009730	5.41E-06
10350630	Fam129a	NM_022018	5.43E-06
10487506	Gm14005	ENSMUST00000	5.50E-06
10582669	Ttc13	NM_145607	5.58E-06
10601581	9230105E10Rik	NM_001146007	5.62E-06
10411899	2410002O22Rik	NM_025879	5.63E-06
10520862	Fosl2	NM_008037	5.77E-06
10547916	Ptms	NM_026988	5.79E-06
10525419	P2rx7	NM_011027	5.88E-06
10428672	Dscc1	NM_183089	6.06E-06
10377467	Hes7	NM_033041	6.08E-06
10493177	Mef2d	NM_133665	6.32E-06
10359948	Uap1	NM_133806	6.56E-06
10409276		---	6.57E-06
10445357	Runx2	NM_001145920	6.81E-06
10447317	Epas1	NM_010137	6.86E-06
10420532	Atp8a2	NM_015803	6.91E-06
10529689	Wdr1	NM_011715	7.00E-06
10423663	Vps13b	NM_177151	7.14E-06
10452709	Ndc80	NM_023294	7.15E-06
10557560	Cdipt	NM_026638	7.45E-06
10547906	Lag3	NM_008479	7.57E-06
10461558	Slc15a3	NM_023044	7.62E-06
10353844	Neurl3	NM_153408	7.63E-06
10389606	Prr11	NM_175563	7.78E-06
10395273	Gdap10	BC052902	7.82E-06
10531610	Rasgef1b	NM_145839	7.95E-06
10455238	Ndfip1	NM_022996	7.97E-06
10382200	Ccdc46	NM_029606	7.99E-06
10485963	Arhgap11a	NM_181416	8.02E-06
10394674	Socs2	NM_007706	8.10E-06
10445640	Trerf1	NM_001097623	8.30E-06
10386916	BC046404	BC053455	8.32E-06
10500034	Psmb4	NM_008945	8.41E-06
10400030	Bzw2	NM_025840	8.42E-06
10390707	Top2a	NM_011623	8.46E-06
10578191		---	8.60E-06
10559837	Vmn2r29	NR_003555	8.68E-06
10540795	Irak2	NM_172161	8.72E-06
10575302	Ap1g1	NM_009677	8.75E-06
10501164	Csf1	NM_007778	8.80E-06
10483381	Stk39	NM_016866	8.88E-06
10360985	Cenpf	NM_001081363	8.95E-06
10406254	Ell2	NM_138953	8.97E-06
10581308	Myl6	NM_010860	9.04E-06
10467842	Got1	NM_010324	9.24E-06
10381526	Ppih	NM_001110130	9.61E-06
10472994	Mtx2	NM_016804	9.76E-06
10367945	Phactr2	NM_001033257	9.81E-06
10538142	Gimap5	NM_175035	9.93E-06
10519951	Pion	NM_175437	9.94E-06
10342579		---	9.95E-06
10379060	Proca1	NM_001045516	1.00E-05
10389025	Myo1d	NM_177390	1.01E-05
10407327	Emb	NM_010330	1.02E-05
10586844	Adam10	NM_007399	1.03E-05
10534873	Fbxo24	ENSMUST00000	1.03E-05
10414256	Rbmxrt	NM_009033	1.04E-05
10411019	Msh3	NM_010829	1.09E-05
10552143	Slc7a10	NM_017394	1.14E-05
10373709	Eif4enif1	NM_023743	1.14E-05
10562637	Ccnb1	NM_172301	1.16E-05
10510574	Errfi1	NM_133753	1.17E-05
10455813	Lmnb1	NM_010721	1.18E-05
10465059	Ctsw	NM_009985	1.21E-05
10472860	Rapgef4	NM_019688	1.24E-05
10424779	Cks2	NM_025415	1.25E-05
10512030	3110043O21Rik	BC076612	1.25E-05
10445875	Btg3	NM_009770	1.27E-05
10560315	Ppp5c	NM_011155	1.28E-05
10550332	Slc1a5	NM_009201	1.29E-05
10606948	Morc4	NM_029413	1.29E-05
10546066	Isy1	NM_133934	1.30E-05
10581926	Adat1	NM_013925	1.32E-05
10411739	Ccnb1	NM_172301	1.36E-05
10571312	Dusp4	NM_176933	1.37E-05
10605090	Idh3g	NM_008323	1.38E-05
10403076		---	1.38E-05
10523766	Lrrc8c	NM_133897	1.41E-05
10554629	Eftud1	NM_175317	1.41E-05
10399087	Ncapg2	NM_133762	1.41E-05
10401428	C130039O16Rik	ENSMUST00000	1.42E-05
10361771	Plagl1	NM_009538	1.43E-05
10353004	Cks2	NM_025415	1.45E-05
10404840	Cd83	NM_009856	1.45E-05
10513154	Ptpn3	NM_011207	1.46E-05
10407012	Sfrs12ip1	NM_026075	1.46E-05
10576034	Irf8	NM_008320	1.47E-05
10511416	Tox	NM_145711	1.47E-05
10370259	Col18a1	NM_009929	1.48E-05
10481634	Slc25a25	NM_146118	1.49E-05
10379721	Ccl4	NM_013652	1.49E-05
10523354	Gapdh	NM_008084	1.50E-05
10384552	Gapdh	NM_008084	1.50E-05
10532027	Gapdh	NM_008084	1.50E-05
10405400	Nsd1	NM_008739	1.50E-05
10509568	Camk2n1	NM_025451	1.52E-05
10513818	Stmn1	NM_019641	1.53E-05
10513181	Gapdh	NM_008084	1.53E-05
10391301	Stat3	NM_213659	1.54E-05
10508986	Stmn1	NM_019641	1.55E-05
10488195	Rrbp1	NM_024281	1.56E-05
10407211	Ppap2a	NM_008247	1.56E-05
10539026	Immt	NM_029673	1.56E-05
10483046	Dpp4	NM_010074	1.57E-05
10501895	Myoz2	NM_021503	1.59E-05
10547590	Klrg1	NM_016970	1.61E-05
10458589	Prelid2	NM_029942	1.61E-05
10586240	Dennd4a	NM_001162917	1.62E-05
10346799	Icos	NM_017480	1.62E-05
10483110	Ifih1	NM_027835	1.63E-05
10522024	Tbc1d1	NM_019636	1.65E-05
10507112	Stil	NM_009185	1.67E-05
10393881	Mafg	NM_010756	1.69E-05
10574027	Mt1	NM_013602	1.70E-05
10381006	Thra	NM_178060	1.72E-05
10412298	Itga1	NM_001033228	1.76E-05
10363070	Gp49a	NM_008147	1.76E-05
10580969	Got2	NM_010325	1.76E-05
10384579	Ugp2	NM_139297	1.77E-05
10550179		---	1.79E-05
10573637	Phkb	NM_199446	1.80E-05
10511952	Orc3l	NM_015824	1.83E-05

Rasgrf1	1.98	3.43E-07
Plp2	1.98	6.69E-05
Ppil5	1.98	2.73E-04
Srgn	1.97	3.84E-05
F630043A04Rik	1.97	4.51E-04
Cdca5	1.96	2.33E-03
Spag5	1.96	5.18E-04
Dtl	1.95	3.42E-04
Frmd5	1.95	4.04E-07
Gm6132	1.95	1.17E-03
Prim1	1.95	1.38E-03
Ect2	1.95	7.00E-05
Kpna2	1.95	3.00E-04
Itgb8	1.94	3.64E-05
Gm4951	1.94	3.82E-04
Stk32c	1.94	2.31E-07
Wee1	1.94	1.67E-03
Clic4	1.93	3.62E-05
Mlkl	1.93	9.35E-04
Ppp2r3a	1.93	4.55E-06
Tipin	1.93	6.40E-04
Clspn	1.92	7.63E-04
Plk3	1.91	3.72E-05
S100a6	1.91	7.27E-07
Asns	1.91	1.06E-05
Smc2	1.91	9.12E-04
Iigp1	1.89	1.92E-04
Hist1h4d	1.89	2.44E-03
Hist1h2ab	1.89	9.54E-04
Raph1	1.89	5.41E-06
Hist1h4m	1.88	3.49E-03
Ccl3	1.87	2.17E-04
Il10ra	1.87	4.86E-06
Cenpw	1.87	2.76E-04
Diap3	1.86	1.12E-03
4930412O13Rik	1.86	1.27E-05
Lig1	1.85	1.68E-05
Kif18a	1.85	7.21E-05
Pcsk1	1.85	2.95E-05
Dlgap5	1.85	5.52E-04
Hmgb2	1.85	3.13E-05
Racgap1	1.85	3.02E-04
Wars	1.85	5.05E-05
Socs2	1.84	4.85E-05
Fam129a	1.84	9.70E-06
Hist2h2bb	1.84	4.20E-05
Hist1h4b	1.84	3.54E-03
Ndc80	1.83	3.11E-05
Higd1a	1.83	4.73E-03
Ints4	1.82	2.83E-06
Mapkapk2	1.82	2.51E-06
Gm14005	1.81	1.41E-08
Ctnna1	1.81	8.66E-06
Angptl2	1.81	1.11E-06
Adam8	1.81	2.47E-06
Aspn	1.80	4.63E-03
BC023105	1.80	2.18E-03
Serpinb6a	1.80	9.25E-05
Irf8	1.80	5.54E-07
Bnip3	1.79	2.96E-04
Mlf1	1.79	1.13E-04
Mad2l1	1.79	4.63E-05
Itpripl2	1.79	6.85E-04
Obfc2a	1.79	1.60E-07
Penk	1.78	5.27E-05
Ubash3b	1.78	4.86E-06
Rassf6	1.78	1.06E-04
Lamp2	1.78	8.71E-04
Pole	1.78	2.57E-04
Hist2h2ab	1.77	3.15E-04
Plk1	1.77	2.32E-04
Chst2	1.76	7.14E-05
Gbp6	1.76	1.49E-04
Mcm6	1.75	2.16E-04
Rad54b	1.74	2.57E-04
Eno1	1.74	1.31E-04
Hmgn5	1.74	1.75E-04
11-Sep	1.73	3.47E-04
Ppa1	1.72	9.04E-06
Grn	1.72	1.33E-04
Plin2	1.72	3.47E-04
Myo1f	1.71	8.25E-05
Cyp11a1	1.70	3.51E-05
Itih5	1.70	7.39E-05
Whsc1	1.70	4.86E-06
Klf12	1.70	9.46E-04
Incenp	1.70	5.98E-05
Ctsb	1.69	1.92E-07
Fignl1	1.69	9.93E-04
Il12rb2	1.69	6.14E-04
Atxn1	1.69	1.20E-05
Fam185a	1.69	8.11E-05
Il18rap	1.69	1.33E-05
Tmbim1	1.69	4.43E-05
Hpse	1.68	1.11E-03
Gmnn	1.68	6.29E-03
6720489N17Rik	1.68	3.32E-03
Ahcy	1.68	1.95E-05
Fam82a1	1.68	9.55E-05
Slc25a24	1.67	1.65E-04
Map2k3	1.67	3.93E-04
Sytl2	1.67	2.27E-05
Gm71	1.67	5.55E-04
Camk2n1	1.67	2.04E-05
Sap30	1.67	7.18E-03
D14Ertd449e	1.67	9.58E-05
Skap2	1.67	6.01E-05
Gm11277	1.66	4.26E-04
Gbp2	1.66	9.64E-04
Osbpl3	1.66	1.54E-06
Il1rn	1.66	5.82E-05
Mybl1	1.66	2.11E-04
Tec	1.66	1.83E-04
Atf6	1.66	1.63E-04
Mpa2l	1.65	7.50E-05
Rilpl2	1.65	8.23E-04
Cenpp	1.65	2.52E-03
Gm13959	1.65	6.21E-03
Gimap7	1.64	3.07E-04
Cd200r4	1.64	1.42E-03
Ifitm2	1.64	3.90E-03
4930547N16Rik	1.64	1.47E-03
Syt11	1.64	5.47E-05
Tgfbr1	1.64	6.00E-04
Pfkp	1.64	2.60E-07
Kif2c	1.64	5.23E-04
5730590G19Rik	1.64	3.58E-03
Cln3	1.63	6.10E-05

Fnip2	1.57	2.65E-05	1.59	1.10E-02
Gapdh	1.55	4.11E-06	1.68	3.56E-04
Ern1	1.54	2.64E-05	2.21	4.48E-04
Gapdh	1.53	1.53E-07	1.61	9.42E-04
Galc	1.52	1.60E-04	1.51	1.66E-03
Rgs1	1.52	8.27E-05	1.61	6.11E-04
Ldha	1.52	3.30E-05	1.69	2.49E-03
Pkm2	1.51	7.28E-05	1.71	1.19E-02
Gdap10	1.51	7.17E-04	2.12	2.33E-05
Cd38	1.51	8.77E-04	-2.00	6.19E-05
Gapdh	1.51	1.51E-05	1.58	6.90E-04
Slc25a19	1.51	1.07E-05	2.02	6.39E-03
Cd274	1.50	1.22E-03	1.71	1.37E-02
Adam9	1.50	4.65E-03	1.89	1.04E-02
Mcoln3	-1.50	8.85E-04	-1.57	2.29E-03
Car15	-1.51	6.04E-04	-1.89	5.07E-04
Usp24	-1.52	5.96E-06	-1.66	6.49E-03
Capn3	-1.53	5.34E-04	-1.67	1.35E-03
Zfp280b	-1.54	1.19E-05	-1.60	1.81E-03
Zfp873	-1.55	3.46E-03	-2.54	7.29E-05
Inpp5f	-1.57	5.38E-05	-1.67	4.02E-03
Sqrdl	-1.57	3.62E-03	-2.18	2.45E-04
Tnfrsf26	-1.58	9.74E-04	-1.73	1.10E-03
Lass6	-1.59	1.94E-03	-1.50	2.86E-04
Bcl2	-1.60	4.70E-04	-2.13	1.43E-03
Zfp442	-1.60	5.74E-03	-1.60	2.14E-03
Gm10825	-1.62	4.16E-03	-2.59	2.30E-03
Lhfpl1	-1.64	3.14E-05	-1.51	2.12E-03
Wrb	-1.64	1.03E-03	-1.79	1.57E-03
Bckdhb	-1.64	5.89E-04	-1.65	2.62E-03
Rassf2	-1.66	2.03E-05	-1.89	1.36E-02
Zc3h12b	-1.66	3.63E-04	-1.57	3.20E-03
Cd83	-1.67	5.41E-04	1.55	5.63E-03
Pdlim1	-1.68	4.36E-03	-1.70	5.74E-05
Rapgef4	-1.68	6.74E-06	-1.56	4.59E-04
Nedd4l	-1.68	3.26E-05	-1.66	4.41E-03
Nod1	-1.68	3.12E-04	-1.89	2.40E-05
Eml5	-1.69	6.36E-06	-1.57	5.02E-03
Ppap2a	-1.70	3.00E-06	-1.51	9.72E-04
Fcrl1	-1.70	7.37E-04	-2.22	2.34E-05
Myo9a	-1.70	7.52E-06	-1.93	3.75E-03
Plekha1	-1.71	1.26E-03	-1.93	1.05E-02
Cetn4	-1.72	1.81E-03	-2.03	1.85E-03
Zbtb20	-1.74	4.07E-04	-1.58	3.08E-04
Dennd2d	-1.77	2.61E-05	-1.75	6.35E-04
Klf3	-1.77	1.88E-03	-2.15	1.17E-03
Clec2i	-1.77	1.40E-04	-1.81	1.22E-02
H2-Ob	-1.82	4.40E-05	-2.15	8.15E-04
Ms4a4c	-1.85	4.34E-03	-1.93	1.49E-03
Asap1	-1.85	2.73E-06	-2.03	4.04E-03
Art3	-1.86	1.54E-03	-1.75	8.90E-03
Prrg1	-1.89	1.10E-04	-1.68	2.61E-03
Mctp1	-1.90	9.19E-06	-2.14	3.35E-04
Khdc1a	-1.91	5.53E-03	-2.06	3.00E-03
Cd226	-1.97	6.15E-05	-3.19	3.70E-05
Evl	-2.01	3.12E-06	-1.71	1.03E-02
1700025G04Ri	-2.02	2.59E-04	-1.50	1.35E-02
Tbc1d4	-2.05	2.72E-06	-1.62	6.17E-03
Dzip1	-2.11	4.21E-06	-1.85	6.31E-03
Dpp4	-2.12	5.35E-05	-3.17	1.54E-05
Cd96	-2.33	1.99E-04	-2.31	1.67E-02
Ift80	-2.38	3.21E-05	-2.36	1.03E-02
Gm14085	-2.40	1.09E-04	-2.03	1.56E-02
Btla	-2.43	2.23E-05	-1.97	5.85E-03
Tlr7	-2.55	1.02E-04	-2.12	2.09E-03
5830405N20Ri	-2.63	7.56E-08	-2.12	1.25E-03
Ces2d-ps	-2.73	1.67E-08	-1.79	7.06E-04
Cxcr5	-3.02	1.93E-04	-3.22	2.66E-05
Parp8	-3.43	3.22E-06	-2.31	5.84E-03

BC017612	1.68	1.54E-03
Gna13	1.68	3.82E-04
Isy1	1.67	7.47E-05
Pmaip1	1.67	1.29E-04
Mif4gd	1.66	1.05E-03
Fam117b	1.66	2.56E-04
Mpzl1	1.65	7.70E-03
Dgat2	1.64	4.30E-03
Stat5a	1.64	4.19E-06
Gm6981	1.63	6.52E-03
Phlda1	1.63	7.50E-03
Gm889	1.63	4.52E-04
Kdm6b	1.63	1.49E-03
Gm4609	1.62	8.36E-04
Ctsh	1.62	5.93E-03
Klrc2	1.62	1.43E-02
Gapdh	1.62	9.64E-04
Avpi1	1.62	6.32E-04
Nr1d1	1.62	7.65E-04
Ier3	1.62	8.13E-03
Stx11	1.60	4.01E-03
Cenpa	1.60	4.15E-05
Wnk1	1.60	4.33E-03
H3f3b	1.60	1.57E-03
Ndel1	1.60	7.21E-04
Dusp1	1.59	2.05E-04
4933407C03Rik	1.59	1.75E-06
Flnb	1.59	6.82E-03
Hlf	1.59	1.11E-02
Pja1	1.59	5.39E-04
4932438A13Rik	1.59	9.31E-03
Gm5523	1.58	1.49E-03
Bcl2l15	1.58	1.18E-03
Dnajb6	1.58	3.17E-03
Olfr32	1.58	9.25E-03
Mpp7	1.58	1.04E-03
Gpr132	1.57	1.21E-05
Csrnp1	1.57	6.70E-05
Elovl4	1.57	1.75E-02
Hemt1	1.57	1.48E-04
Gnai1	1.57	1.64E-02
Cdr2	1.57	5.65E-03
Mir22	1.57	7.16E-03
6330407A03Rik	1.57	9.98E-03
Ms4a4a	1.57	3.38E-04
Junb	1.57	1.01E-03
Ccdc117	1.56	7.25E-03
Gm12070	1.56	4.87E-04
Sprr1a	1.56	1.15E-02
Lzts1	1.56	5.16E-03
Tlcd1	1.55	1.34E-03
E130201H02Rik	1.55	4.73E-03
Med7	1.55	7.90E-05
6030498E09Rik	1.55	9.87E-04
Birc2	1.55	3.57E-03
Ap3s1	1.54	5.97E-03
Agr2	1.54	7.96E-03
Hmcn1	1.54	1.08E-02
Smox	1.54	7.75E-05
Aff1	1.54	1.44E-04
3110007F17Rik	1.54	1.67E-03
Rap2a	1.54	1.34E-02
Ngfrap1	1.54	3.12E-03
Ulk1	1.54	5.23E-04
Tcfap2a	1.53	7.19E-03
Pqlc1	1.53	1.79E-04
Atad2	1.53	8.73E-03
Tmem65	1.53	6.00E-04
Nudt4	1.53	5.23E-03
Spag9	1.53	4.92E-03
Ubc	1.53	5.07E-04
Kif3a	1.53	6.05E-03
Akap2	1.53	7.37E-03
Cyr61	1.53	2.76E-03
Fam110a	1.53	1.14E-03
Ubr2	1.53	4.89E-04
Il33	1.52	2.40E-03
Hopx	1.52	1.15E-04
Ubc	1.52	5.39E-04
Map1lc3b	1.52	1.75E-02
Isca1	1.52	4.44E-04
Cpe	1.52	7.40E-03
Gapdh	1.51	1.82E-03
Dnaja4	1.51	4.88E-04
Olfr419	1.51	4.15E-03
Olfr1507	1.51	7.20E-03
Mif	1.50	3.09E-03
Lrrc32	1.50	6.04E-03
Ddit4	1.50	3.50E-03
Cnot3	-1.50	5.82E-04
Arid2	-1.50	1.83E-03
D830030K20Rik	-1.50	4.10E-03
AI316807	-1.50	6.36E-04
Cdc16	-1.50	1.42E-02
Snx11	-1.50	7.71E-05
Smg6	-1.50	6.02E-03
B430306N03Rik	-1.50	1.29E-03
Ift20	-1.50	1.67E-02
Rps24	-1.50	9.29E-03
Tasp1	-1.50	8.56E-06
Samm50	-1.50	3.82E-03
Zfp438	-1.51	3.92E-03
Tnik	-1.51	3.46E-04
Chd1l	-1.51	2.27E-04
Immt	-1.51	5.71E-06
Tug1	-1.51	1.17E-02
Hp1bp3	-1.51	9.05E-03
Gm3579	-1.51	6.03E-03
Polr2h	-1.51	1.75E-03
Rnasel	-1.51	5.47E-03
Chchd7	-1.51	2.86E-03
Tlr4	-1.51	7.92E-03
Add3	-1.51	4.93E-03
Ylpm1	-1.51	6.23E-03
E330021D16Rik	-1.51	6.18E-03
Rnf139	-1.51	5.15E-03
Parp2	-1.51	4.96E-03
Dgkd	-1.51	1.03E-02
Abcb1a	-1.51	2.22E-03
1810035L17Rik	-1.51	1.73E-02
Pitpnc1	-1.51	5.54E-03
St3gal5	-1.51	4.98E-03
Skap1	-1.51	1.40E-03
B3gnt3	-1.51	3.84E-03
Rbm26	-1.51	2.29E-04
Ddx19b	-1.51	1.66E-02
Rtcd1	-1.51	1.36E-02
Dtymk	-1.51	2.15E-03

10375322	4933415A04Rik	ENSMUST000000	1.83E-05
10367532	5830405N20Rik	NM_183264	1.83E-05
10405185	Cks2	NM_025415	1.84E-05
10478594	Ctsa	NM_008906	1.89E-05
10538901	BC005685	BC005685	1.90E-05
10569707	Myadm	NM_001093765	1.91E-05
10595791		---	1.95E-05
10565193	Hdgfrp3	NM_013886	1.98E-05
10389395	Brip1	NM_178309	1.99E-05
10495279	Psma5	NM_011967	2.02E-05
10406434	Mef2c	NM_025282	2.04E-05
10518350	Hmgb2	NM_008252	2.04E-05
10375443	Havcr2	NM_134250	2.04E-05
10592535	Sorl1	NM_011436	2.04E-05
10504757	BC005685	BC005685	2.05E-05
10590631	Ccr2	NM_009915	2.07E-05
10588223	Anapc13	NM_181394	2.07E-05
10471154	Ass1	NM_007494	2.09E-05
10586252	Dennd4a	NM_001162917	2.11E-05
10477946	Rpn2	NM_019642	2.16E-05
10560685	Bcl3	NM_033601	2.20E-05
10523138	Cxcl3	NM_203320	2.20E-05
10469278	Il2ra	NM_008367	2.24E-05
10492971	Fcrl1	NM_153090	2.25E-05
10368970	Prdm1	NM_007548	2.32E-05
10426451	Irak4	NM_029926	2.34E-05
10601011	Kif4	NM_008446	2.35E-05
10457168	Cd226	NM_178687	2.36E-05
10439878	Psmc1	NM_008947	2.38E-05
10520521	Cenpa	NM_007681	2.38E-05
10343271		---	2.41E-05
10577508	Ckap2	NM_001004140	2.42E-05
10344012		---	2.44E-05
10369630	Ddx21	NM_019553	2.44E-05
10476989	Gins1	BC027537	2.45E-05
10361897	Ifngr1	NM_010511	2.49E-05
10372965	Usp15	NM_027604	2.51E-05
10393620	Cbx4	NM_007625	2.52E-05
10456005	Cd74	NM_001042605	2.52E-05
10482528	Neb	NM_010889	2.54E-05
10385770	Olfr1372-ps1	BC055827	2.56E-05
10513020	Ikbkap	NM_026079	2.58E-05
10552440	Zfp719	ENSMUST000000	2.59E-05
10357363	E030049G20Rik	NM_001081756	2.60E-05
10587627	Cyb5r4	NM_024195	2.61E-05
10364030	Adora2a	NM_009630	2.61E-05
10513152	Ptpn3	NM_011207	2.62E-05
10391518	Mpp3	NM_007863	2.63E-05
10528102	Crot	NM_023733	2.71E-05
10523647	Aff1	NM_001080798	2.71E-05
10406407	Arrdc3	NM_001042591	2.72E-05
10481344	Gapdh	NM_008084	2.77E-05
10385248	Hmmr	NM_013552	2.79E-05
10556381	Mical2	NM_177282	2.81E-05
10447065	Fam82a1	BC024059	2.84E-05
10503134	Sdcbp	NM_001098227	2.86E-05
10456357	Pmaip1	NM_021451	2.86E-05
10423180	Gapdh	NM_008084	2.91E-05
10476814	Insm1	NM_016889	2.93E-05
10430834	Naga	NM_008669	2.96E-05
10548892	Arhgdib	NM_007486	2.99E-05
10543709	Tmem209	NM_178625	3.01E-05
10559667	Il11	NM_008350	3.01E-05
10470206	Lcn9	NM_029959	3.04E-05
10459604	4933403F05Rik	NM_153794	3.06E-05
10542108	Tom1	NM_011622	3.07E-05
10420097	Tinf2	NM_145705	3.08E-05
10374228	Gm11992	NM_001037928	3.09E-05
10503098	Lyn	NM_001111096	3.14E-05
10586244	Dennd4a	NM_001162917	3.14E-05
10500445	Chd1l	NM_026539	3.16E-05
10589889	Glb1	NM_009752	3.17E-05
10509838	Padi2	NM_008812	3.19E-05
10586448	2810417H13Rik	NM_026515	3.20E-05
10445507	Gm88	BC147714	3.21E-05
10549899	Zfp418	NM_146179	3.21E-05
10587616	Prss35	NM_178738	3.23E-05
10518300	Tnfrsf1b	NM_011610	3.23E-05
10359890	Nuf2	NM_023284	3.24E-05
10401935	BC005685	BC005685	3.30E-05
10469138	4930412O13Rik	NR_024257	3.31E-05
10532744	Selplg	NM_009151	3.31E-05
10363735	Egr2	NM_010118	3.32E-05
10515399	Plk3	NM_013807	3.32E-05
10438666		---	3.40E-05
10585778	Sema7a	NM_011352	3.43E-05
10521950	Stim2	NM_001081103	3.45E-05
10520390	Gapdh	BC092252	3.46E-05
10497122	Depdc1a	NM_029523	3.47E-05
10563780	E2f8	NM_001013368	3.49E-05
10587854	Slc9a9	NM_177909	3.50E-05
10395142	Sh3yl1	NM_013709	3.51E-05
10415444		---	3.51E-05
10482687	Arl5a	NM_182994	3.52E-05
10437160	Ets2	NM_011809	3.52E-05
10527598	Pomp	NM_025624	3.52E-05
10427035	Nr4a1	NM_010444	3.62E-05
10423599	Matn2	NM_016762	3.66E-05
10354542	Pms1	NM_153556	3.72E-05
10568873	Adam8	NM_007403	3.73E-05
10358589	Hmcn1	NM_001024720	3.77E-05
10439762	Ahcy	NM_016661	3.77E-05
10497831	Ccna2	NM_009828	3.82E-05
10440393	Samsn1	NM_023380	3.85E-05
10426435	Gm12070	NR_002890	3.88E-05
10544243	Mrps33	NM_010270	3.89E-05
10538126	Gimap4	NM_174990	3.90E-05
10476301	Smox	NM_145533	3.93E-05
10593015	Cd3g	NM_009850	3.93E-05
10510552	Rere	NM_001085492	3.93E-05
10566358	Trim30	NM_009099	3.94E-05
10393272	Rnf157	BC053070	3.96E-05
10526832		---	4.00E-05
10547009	Vgll4	NM_177683	4.04E-05
10413853	Parg	NM_011960	4.04E-05
10513162	Ptpn3	NM_011207	4.06E-05
10474381	Kif18a	NM_139303	4.08E-05
10493009	Arhgef11	NM_001003912	4.09E-05
10553516	Gapdh	NM_008084	4.11E-05
10605554	Gapdh	NM_008084	4.11E-05
10477929	1110008F13Rik	NM_026124	4.12E-05
10411622	Birc1f	NM_010871	4.16E-05
10447264	Slc3a1	NM_009205	4.21E-05
10498405	Gm12070	NR_002890	4.21E-05
10411728	Cenph	NM_021886	4.21E-05
10497682	Kcnmb3	XM_001475546	4.28E-05
10554445	Prc1	NM_145150	4.28E-05
10518352	Hmgb2	NM_008252	4.32E-05

Hist1h2bb	1.63	4.58E-04
Chsy1	1.63	6.62E-06
Tm7sf3	1.63	7.86E-04
Eea1	1.63	3.51E-06
Batf	1.63	1.69E-06
Ywhah	1.62	1.21E-03
Cycs	1.61	2.38E-03
5330426P16Rik	1.61	2.35E-03
Txn1	1.61	6.33E-06
Glipr1	1.61	9.01E-04
Lrrc16a	1.61	8.08E-05
Mnd1	1.61	3.16E-03
Pla2g16	1.61	1.58E-04
Cdc6	1.60	3.01E-04
Rnf19b	1.60	1.66E-06
Mcm3	1.60	1.37E-05
Mapkapk3	1.60	1.10E-04
Pola1	1.60	5.21E-05
Sgms1	1.60	4.11E-06
D2Ertd750e	1.60	6.76E-04
Kif22	1.60	1.92E-03
Ptgr1	1.59	3.27E-04
Gbp3	1.59	1.12E-03
Ppp1r3f	1.59	4.37E-03
Mad2l1bp	1.59	1.60E-03
Ccr1	1.59	2.63E-03
Ide	1.59	2.28E-05
Cdca2	1.58	2.52E-03
Cysltr1	1.58	6.68E-04
Adap1	1.58	5.40E-07
Ryk	1.58	3.12E-04
Hist1h3g	1.58	8.72E-04
Isg20	1.58	3.64E-06
Spc24	1.58	1.43E-04
Gm10906	1.58	1.23E-03
2310061C15Rik	1.58	3.50E-03
Rrbp1	1.58	1.59E-07
Irgm1	1.57	6.88E-03
Got2	1.57	1.19E-04
Cyp20a1	1.57	6.29E-04
Hist1h4c	1.57	2.84E-04
Syce2	1.57	2.47E-03
Ube2e3	1.57	4.72E-03
Pbk	1.57	1.21E-03
Gbp4	1.57	3.38E-04
Tceb3	1.57	1.17E-03
Ptprj	1.57	1.78E-05
Trerf1	1.57	1.27E-06
Psma5	1.56	4.71E-06
Brca1	1.56	1.01E-04
Slc43a3	1.56	1.42E-04
Hspa13	1.56	6.02E-05
Ehd4	1.56	3.48E-05
Ddx28	1.55	2.34E-04
Bcl2a1c	1.55	8.32E-05
Tyms	1.55	1.98E-03
Nab1	1.55	1.40E-04
Hmgb3	1.55	1.58E-03
Ckap2l	1.55	8.99E-04
Cdk1	1.55	3.93E-03
Alms1	1.55	1.74E-04
Tmem154	1.55	1.99E-04
Irgm2	1.54	4.75E-03
AA467197	1.54	1.49E-04
Ska1	1.54	1.25E-05
Baz1a	1.54	2.91E-04
Ifitm1	1.54	7.24E-05
Bcl2a1a	1.54	2.88E-04
Cmtm7	1.54	6.40E-07
Mtmr10	1.54	6.92E-04
M6pr	1.53	6.04E-05
Prr13	1.53	2.91E-05
Bard1	1.53	7.68E-04
Oit3	1.53	1.03E-04
Crip1	1.53	4.90E-06
Pmch	1.53	3.24E-03
Vps54	1.53	6.60E-08
Optn	1.53	2.88E-07
Ncapd2	1.53	4.39E-04
F730047E07Rik	1.53	1.77E-04
Asb2	1.53	1.42E-07
Gna15	1.53	3.48E-06
C920008G01Rik	1.53	5.76E-04
Selplg	1.53	1.04E-06
Skil	1.52	1.46E-05
Dusp5	1.52	5.99E-05
Nid2	1.52	1.82E-05
Cst3	1.52	3.15E-04
Reep5	1.52	3.72E-04
Cit	1.52	6.52E-04
Rps27l	1.52	5.64E-04
Foxm1	1.52	4.25E-03
Aplp2	1.51	1.52E-05
Dctpp1	1.51	8.71E-05
Chaf1a	1.51	9.91E-04
Mcm4	1.51	5.90E-05
4930506M07Rik	1.51	8.69E-04
Zbp1	1.51	3.80E-04
Hist1h2bc	1.51	2.83E-03
Gbp5	1.51	1.48E-03
Gm5068	1.51	1.32E-03
Pnp2	1.51	1.61E-04
Ugp2	1.51	7.58E-06
Tmem159	1.51	3.12E-03
Ccdc18	1.50	9.18E-04
Adam19	1.50	3.71E-04
Sytl3	1.50	1.45E-03
Lman1	1.50	4.45E-03
Maf	1.50	7.13E-03
AB124611	-1.50	5.02E-03
Bbs2	-1.50	3.71E-04
Il6st	-1.51	6.13E-04
Tcte3	-1.51	3.20E-03
Noxa1	-1.51	4.43E-05
A630033H20Rik	-1.51	3.14E-03
Txk	-1.51	5.51E-04
Pik3ip1	-1.52	4.79E-06
Dtx1	-1.53	6.44E-06
Carns1	-1.53	3.34E-04
Slamf6	-1.53	2.21E-03
Rwdd3	-1.54	4.41E-03
Pak1	-1.54	2.14E-03
Ttc28	-1.54	1.90E-03
Dirc2	-1.55	3.11E-04
Il6ra	-1.55	6.50E-05
Si	-1.55	7.95E-05
4930524L23Rik	-1.56	2.96E-03
Slc7a10	-1.56	5.77E-06

Xrn1	-1.51	1.42E-04
Ipo8	-1.51	2.37E-03
Usp33	-1.51	3.29E-03
Rps12	-1.52	1.04E-02
Tmem181a	-1.52	2.90E-03
Kidins220	-1.52	6.33E-03
Ggps1	-1.52	3.74E-04
4933424B01Rik	-1.52	1.51E-02
Lrrcc1	-1.52	4.78E-03
Cul3	-1.52	8.46E-04
Adar	-1.52	5.17E-03
Arfgef1	-1.52	1.31E-04
Camta1	-1.52	6.59E-04
Ttc15	-1.52	1.25E-05
Crls1	-1.52	8.96E-03
Exoc3	-1.52	1.12E-02
Lsm7	-1.52	2.63E-04
Fam73a	-1.52	3.54E-03
Caprin2	-1.52	3.08E-03
Fam36a	-1.52	3.91E-03
Pprc1	-1.52	1.06E-02
Fbxl4	-1.52	1.10E-02
Fbxo25	-1.52	6.18E-03
Naip2	-1.52	1.44E-04
Wdr70	-1.52	1.59E-02
Eif2s3y	-1.52	1.65E-02
Cdk13	-1.52	4.15E-03
Uty	-1.52	1.52E-02
Fam116a	-1.52	5.75E-04
Rpp40	-1.52	7.58E-04
Zfp74	-1.52	1.20E-03
A430033K04Rik	-1.53	3.40E-03
Itgal	-1.53	1.40E-03
Erap1	-1.53	2.62E-03
Apbb1ip	-1.53	8.73E-05
Lrrc40	-1.53	1.46E-03
Usp28	-1.53	7.13E-03
Uso1	-1.53	2.14E-03
Pts	-1.53	4.64E-04
Papolg	-1.53	6.05E-04
Cenpo	-1.53	4.06E-04
Zfp280d	-1.53	6.33E-03
Tm6sf1	-1.53	1.54E-03
Cd47	-1.53	4.30E-03
D030074E01Rik	-1.53	3.11E-04
A130082M07Rik	-1.53	3.44E-06
Man2a1	-1.53	7.35E-04
Osgin2	-1.53	4.81E-03
Ppil3	-1.53	6.28E-04
Morc3	-1.53	1.02E-03
Gm4979	-1.53	8.61E-03
Ppp3ca	-1.53	5.45E-03
Zfp120	-1.53	5.72E-04
Fam120b	-1.53	2.58E-03
Macf1	-1.53	1.14E-02
Glce	-1.53	2.08E-03
C87436	-1.53	1.72E-04
Rasa2	-1.53	7.18E-03
6330409D20Rik	-1.53	4.55E-03
Tomm7	-1.53	4.51E-04
Cog3	-1.53	2.11E-03
Sec22a	-1.53	2.57E-04
6720457D02Rik	-1.53	2.63E-05
Mlf1ip	-1.53	1.29E-02
Ppp2r5a	-1.54	5.80E-04
Stim2	-1.54	2.07E-04
Dapp1	-1.54	1.19E-02
Sfmbt1	-1.54	8.49E-03
Ccdc38	-1.54	2.46E-03
Zcchc7	-1.54	1.09E-02
Utrn	-1.54	3.73E-03
Fancl	-1.54	1.93E-03
Papola	-1.54	1.62E-03
Ube2q2	-1.54	1.34E-02
Adat1	-1.54	5.57E-05
Fcf1	-1.54	1.74E-02
D17Wsu104e	-1.54	3.82E-03
Tfam	-1.54	8.62E-03
Gdpd3	-1.54	2.52E-03
Cd84	-1.54	1.15E-04
Zdhhc15	-1.54	7.90E-03
Fam35a	-1.54	1.10E-02
Pbx2	-1.54	1.20E-02
Rnasen	-1.54	9.28E-04
Prepl	-1.54	9.03E-03
BC013529	-1.54	1.06E-02
Rnmt	-1.54	3.67E-03
Tbc1d23	-1.55	7.07E-03
Ypel1	-1.55	6.03E-03
Cmc1	-1.55	5.29E-03
Zmym1	-1.55	8.13E-03
Snx27	-1.55	2.36E-03
D11Wsu47e	-1.55	1.60E-02
Itgb1	-1.55	7.59E-05
Akr1b3	-1.55	4.85E-04
BC016423	-1.55	1.45E-03
Rars	-1.55	1.68E-03
Dnajc24	-1.55	7.23E-04
Wdr1	-1.55	5.14E-05
Rfesd	-1.55	1.24E-02
Gm4924	-1.55	1.54E-02
Plrg1	-1.55	7.45E-03
2700050L05Rik	-1.55	3.69E-04
Nsd1	-1.55	3.74E-05
Chd8	-1.55	1.78E-03
Rab12	-1.55	1.09E-02
Pvt1	-1.55	1.53E-03
Atp13a3	-1.56	7.25E-03
Vps41	-1.56	1.61E-02
Pik3cg	-1.56	1.56E-02
LOC100134980	-1.56	5.78E-03
Ints8	-1.56	2.33E-03
Zfp760	-1.56	7.88E-03
Rprd2	-1.56	3.46E-05
Smek2	-1.56	4.68E-04
Serpini1	-1.56	2.38E-03
AI987944	-1.56	1.33E-02
Gpr52	-1.56	3.92E-03
Zfp148	-1.56	3.37E-03
Smc1a	-1.56	7.02E-04
Dcun1d2	-1.56	1.26E-04
Mettl3	-1.56	1.09E-02
Fam63b	-1.57	8.28E-03
Ptcd2	-1.57	4.35E-04
Senp1	-1.57	8.50E-04
Actg2	-1.57	6.02E-04
1110037F02Rik	-1.57	1.12E-03
Cfdp1	-1.57	9.75E-03

10462136	Cycs	NM_007808	4.36E-05
10564565	Gm7693	XR_034027	4.38E-05
10363082	Lilrb4	NM_013532	4.41E-05
10430811	Nhp2l1	NM_011482	4.41E-05
10481147	Surf4	NM_011512	4.54E-05
10607710		---	4.56E-05
10377286	Pik3r6	NM_001081566	4.58E-05
10393580	Cant1	NM_029502	4.62E-05
10353008	Gm7075	ENSMUST00000(	4.62E-05
10365199	Ankrd24	AK173316	4.69E-05
10470547	Mrps2	NM_080452	4.69E-05
10594774	Ccnb2	NM_007630	4.72E-05
10574934	Nrn1l	NM_175024	4.72E-05
10472097	Fmnl2	NM_172409	4.74E-05
10538617	Lancl2	NM_133737	4.75E-05
10390691	Nr1d1	NM_145434	4.75E-05
10360648	Psen2	NM_011183	4.78E-05
10408094	Hist1h2ao	NM_178185	4.81E-05
10578545	Gapdh	NM_008084	4.84E-05
10342988		---	4.94E-05
10495147	Dennd2d	NM_001093754	4.96E-05
10345791	Il1rl1	NM_001025602	4.97E-05
10573823	Chd9	NM_177224	5.00E-05
10480585	Noxa1	NM_172204	5.01E-05
10586700	Rora	NM_013646	5.07E-05
10375227		---	5.13E-05
10340661		---	5.18E-05
10544891	Nod1	NM_172729	5.20E-05
10502890	St6galnac3	NM_011372	5.21E-05
10438572	2510009E07Rik	NM_001001881	5.29E-05
10399691	Id2	NM_010496	5.29E-05
10409200	Gapdh	NM_008084	5.31E-05
10603551	Cybb	NM_007807	5.31E-05
10567964	Cln3	NM_001146311	5.31E-05
10374880	Mtif2	NM_133767	5.34E-05
10407946	Stard3nl	NM_024270	5.35E-05
10453334	Lrpprc	NM_028233	5.37E-05
10592888	Cxcr5	NM_007551	5.43E-05
10498935	Gucy1b3	NM_017469	5.49E-05
10411432	Utp15	NM_178918	5.52E-05
10457007	Pqlc1	NM_025861	5.54E-05
10414360	Lgals3	NM_001145953	5.55E-05
10607183	Lhfpl1	NM_178358	5.56E-05
10539727	C87436	NR_027370	5.56E-05
10587508	Ttk	NM_009445	5.58E-05
10463945	Dusp5	NM_001085390	5.68E-05
10440419	Btg3	NM_009770	5.69E-05
10542834	Gm5887	NM_001034903	5.69E-05
10402665	Cdc42bpb	NM_183016	5.70E-05
10563829	Mrps33	NM_010270	5.75E-05
10520419	Ube3c	NM_133907	5.79E-05
10544348	Trpv6	NM_022413	5.81E-05
10404389	Irf4	NM_013674	5.87E-05
10341142		---	5.90E-05
10521731	Ncapg	NM_019438	5.98E-05
10397715		---	5.98E-05
10500237	Rprd2	NM_001081293	6.00E-05
10590494	Kif15	NM_010620	6.01E-05
10439732	Plcxd2	NM_001134480	6.04E-05
10585794	Cyp11a1	NM_019779	6.08E-05
10533781	Mphosph9	NM_001081323	6.10E-05
10404848	Jarid2	NM_021878	6.12E-05
10572497	Il12rb1	NM_008353	6.18E-05
10477572	Chmp4b	NM_029362	6.20E-05
10384725	Rel	NM_009044	6.24E-05
10359339	Rabgap1l	NM_013862	6.27E-05
10338706		---	6.30E-05
10470936	Tbc1d13	NM_146252	6.32E-05
10492002	Gm10727	ENSMUST00000(	6.35E-05
10572989	Slc10a7	NM_029736	6.36E-05
10368644	Fam26f	NM_175449	6.38E-05
10502732	Prkacb	NM_011100	6.43E-05
10587558	Dopey1	NM_177208	6.48E-05
10476969	Pygb	NM_153781	6.54E-05
10352048	Exo1	NM_012012	6.58E-05
10447699	6530411M01Rik	ENSMUST00000(	6.58E-05
10474769	Bub1b	NM_009773	6.61E-05
10406334	Mctp1	NM_030174	6.63E-05
10535025	Got2	NM_010325	6.66E-05
10507677	Hivep3	NM_010657	6.72E-05
10517287	Man1c1	NM_207237	6.74E-05
10465772	Slc3a2	NM_008577	6.75E-05
10426835	Dip2b	NM_001159361	6.77E-05
10437945	Mcm4	NM_008565	6.77E-05
10466521	Gcnt1	NM_173442	6.77E-05
10514128	Ttc39b	NM_027238	6.80E-05
10516051	Cap1	NM_007598	6.84E-05
10362758	Zbtb24	NM_153398	6.84E-05
10554013	Chsy1	NM_001081163	6.87E-05
10496569	Gbp6	NM_145545	6.87E-05
10440929	Gart	NM_010256	6.89E-05
10586616	Vps13c	NM_177184	7.00E-05
10340104		---	7.02E-05
10438198	Med15	NM_033609	7.06E-05
10364559	Arid3a	NM_007880	7.08E-05
10338938		---	7.11E-05
10499431	Syt11	NM_018804	7.14E-05
10439710	Phldb2	NM_153412	7.27E-05
10400589	C79407	BC052175	7.31E-05
10426999	Acvrl1	NM_009612	7.33E-05
10560304	Calm3	NM_007590	7.34E-05
10388734	Eral1	NM_022313	7.35E-05
10390258	Snx11	NM_028965	7.36E-05
10581645	Marveld3	NM_028584	7.37E-05
10378508	Tsr1	NM_177325	7.38E-05
10493484	Krtcap2	NM_025327	7.42E-05
10479981	Gata3	NM_008091	7.48E-05
10562639	Gapdh	NM_008084	7.49E-05
10527982	A330021E22Rik	BC062906	7.50E-05
10490221	Atp5e	NM_025983	7.53E-05
10428827	Tmem65	NM_175212	7.55E-05
10469575	Gm13363	NR_002688	7.58E-05
10400095	Ifrd1	NM_013562	7.59E-05
10507529	Med8	NM_020000	7.62E-05
10389207	Ccl5	NM_013653	7.64E-05
10446986	Crim1	NM_015800	7.70E-05
10544106	Zc3hav1	ENSMUST00000	7.76E-05
10481540	Fnbp1	NM_001038700	7.78E-05
10491091	Tnfsf10	NM_009425	7.84E-05
10438791	Uts2d	NM_198166	7.88E-05
10484261	Cerkl	NM_001048176	7.92E-05
10404895	5033430I15Rik	ENSMUST00000(	7.99E-05
10457205	Crem	NM_001110856	8.02E-05
10464045	Acsl5	NM_027976	8.07E-05
10392388	Prkca	NM_011101	8.08E-05
10379630	Slfn2	NM_011408	8.10E-05
10573319	Podnl1	NM_001013384	8.18E-05
10453867	Rbbp8	NM_001081223	8.18E-05

Amigo2	-1.56	8.73E-04
Sesn1	-1.57	8.37E-04
Slc9a7	-1.58	1.52E-03
Acta2	-1.58	2.39E-03
Klhl6	-1.59	1.34E-04
Gpr34	-1.59	5.41E-03
Ehd3	-1.59	1.64E-04
Kif1b	-1.59	2.01E-04
Cebpa	-1.61	4.34E-03
Arhgef3	-1.61	5.08E-05
A630001O12Rik	-1.61	7.74E-04
D18Ertd653e	-1.62	1.13E-05
Hdgfrp3	-1.64	8.41E-06
Plagl1	-1.65	3.21E-05
Orm3	-1.65	3.20E-04
Pecam1	-1.65	4.18E-04
Wwp1	-1.65	1.68E-04
Cmah	-1.66	1.09E-05
Zbtb20	-1.67	1.29E-05
Slc35d1	-1.67	1.22E-04
Bach2	-1.68	2.22E-05
Nbn	-1.68	2.77E-05
Scmh1	-1.68	6.14E-05
Sh3bgrl2	-1.68	8.86E-04
A130049A11Rik	-1.69	1.62E-03
Rab3ip	-1.69	1.34E-04
Gucy1a3	-1.70	4.86E-05
Dscam	-1.70	2.01E-06
Pgap1	-1.71	1.49E-03
Bzw2	-1.75	1.51E-06
Cd200	-1.76	7.53E-05
Ccdc50	-1.76	5.21E-03
5830472F04Rik	-1.77	6.11E-03
Gm885	-1.77	1.12E-03
Vmn2r86	-1.78	8.41E-05
Ccr7	-1.79	2.04E-06
Sell	-1.80	1.52E-03
St6gal1	-1.80	3.50E-05
Orm2	-1.81	3.51E-04
St8sia1	-1.81	1.14E-05
Tnfsf8	-1.82	1.80E-04
Pik3r3	-1.83	1.49E-03
Snord57	-1.83	7.37E-03
Cd2ap	-1.84	3.84E-04
Cd40lg	-1.85	4.25E-04
Lrig1	-1.85	1.11E-07
4930420K17Rik	-1.88	2.20E-04
Vcam1	-1.92	8.14E-04
Nsg2	-1.93	2.41E-05
Qser1	-1.94	1.60E-05
Foxp1	-1.95	8.57E-06
Arhgap20	-1.95	4.01E-05
Ugcg	-1.99	3.14E-04
Hdac9	-2.00	7.73E-06
Ssbp2	-2.02	6.13E-05
St8sia6	-2.05	3.43E-03
Snord32a	-2.06	1.04E-03
Sgk3	-2.07	2.66E-05
D130062J21Rik	-2.13	5.67E-06
Atp1b1	-2.14	1.08E-04
Ces2c	-2.15	8.47E-05
Nrn1	-2.17	2.15E-06
Gsta4	-2.29	2.09E-03
Ccr6	-2.30	2.14E-06
Aff3	-2.34	4.24E-05
Arhgap5	-2.40	2.45E-04
Tcf7	-2.42	1.14E-06
Cd86	-2.55	4.12E-05
Actn1	-2.57	5.39E-06
Mir15b	-4.48	1.23E-05

Fig4	-1.57	1.56E-02
Traf3ip3	-1.57	4.34E-03
Uvrag	-1.57	3.82E-04
Robld3	-1.57	4.93E-03
Zcchc3	-1.57	1.76E-02
Hadhb	-1.57	2.59E-03
Lyrm5	-1.57	5.68E-03
Usp31	-1.57	1.76E-02
Gpatch1	-1.57	1.56E-03
Prkd2	-1.57	5.18E-04
Dcaf13	-1.57	9.59E-04
Ddx58	-1.57	9.08E-04
Rbm6	-1.57	1.09E-02
Surf1	-1.58	9.21E-03
Nol8	-1.58	1.54E-02
Gm12185	-1.58	1.74E-02
Rpa1	-1.58	1.54E-03
Spred2	-1.58	6.17E-03
Rnf141	-1.58	4.70E-03
Mrpl51	-1.58	1.44E-02
4930473A06Rik	-1.58	1.09E-03
Jkamp	-1.58	3.89E-03
Mrpl3	-1.58	7.34E-03
Heatr1	-1.58	4.94E-03
Polr2b	-1.58	1.49E-03
Rbks	-1.58	1.32E-04
Stambp	-1.58	3.67E-03
Tm9sf2	-1.58	4.56E-03
Ndrg3	-1.58	8.92E-03
1110059E24Rik	-1.58	9.81E-03
5830416P10Rik	-1.58	6.63E-03
Stoml2	-1.58	1.42E-02
Ube4a	-1.59	1.23E-02
Golph3l	-1.59	2.61E-03
Prkcb	-1.59	2.75E-03
Scarna8	-1.59	9.48E-03
Bivm	-1.59	1.43E-02
4930578N16Rik	-1.59	1.18E-04
Lrig2	-1.59	7.74E-04
1500001M20Rik	-1.59	3.21E-04
Ola1	-1.59	1.49E-02
Cept1	-1.59	8.07E-05
Dpy19l3	-1.59	5.03E-03
Rrm2b	-1.59	5.41E-03
2700062C07Rik	-1.59	3.84E-03
Cdc14a	-1.59	6.70E-04
Pyroxd1	-1.59	2.87E-04
Ccdc45	-1.59	1.12E-02
Ino80	-1.59	1.88E-03
Orai3	-1.59	2.88E-03
Ndufs4	-1.59	3.13E-03
Erlin2	-1.59	9.32E-03
Fam120c	-1.59	3.32E-04
Mrps36	-1.59	1.25E-02
Gm12258	-1.59	5.95E-03
Vmn2r29	-1.60	3.90E-06
Herc6	-1.60	4.85E-03
Rb1	-1.60	1.38E-05
Nnt	-1.60	1.20E-03
Pja2	-1.60	1.85E-04
Apobec3	-1.60	1.08E-03
Xrcc5	-1.60	3.80E-03
Zfp607	-1.60	5.14E-03
Lancl1	-1.60	8.47E-04
Wdr82	-1.60	7.53E-03
Mtif3	-1.60	1.62E-03
Psen1	-1.60	4.62E-03
9930111J21Rik2	-1.60	9.36E-03
Ube2q1	-1.60	1.58E-03
5830433M19Rik	-1.60	1.76E-02
Rad1	-1.60	9.70E-03
Ccdc53	-1.60	1.69E-02
Nup88	-1.60	1.28E-02
Sec23ip	-1.60	2.08E-03
Dicer1	-1.60	2.68E-03
Orc5	-1.60	2.55E-04
Nup160	-1.61	3.12E-03
Plxnc1	-1.61	3.78E-03
Phf11	-1.61	5.24E-03
Angptl1	-1.61	8.57E-03
Cwf19l1	-1.61	5.34E-03
5730419I09Rik	-1.61	2.86E-04
Tmem69	-1.61	1.47E-03
Ubr1	-1.61	2.24E-03
Ddx1	-1.61	6.84E-03
Sppl3	-1.61	3.03E-04
Dcaf6	-1.61	1.77E-02
Dhdds	-1.61	1.04E-03
Uqcrb	-1.61	5.39E-03
Zfp300	-1.61	1.45E-02
Acp2	-1.61	5.20E-03
Arl6ip6	-1.61	1.19E-03
Sp110	-1.61	1.49E-02
Ubr3	-1.61	4.46E-04
Prkacb	-1.61	4.55E-05
Brd8	-1.61	2.20E-03
Dock8	-1.61	1.48E-03
Tmem147	-1.61	3.99E-03
Trim30a	-1.61	2.27E-04
Fam178a	-1.61	1.31E-02
Gzf1	-1.62	1.39E-02
Rg9mtd3	-1.62	3.34E-04
Slc12a2	-1.62	9.66E-03
Tbc1d14	-1.62	6.87E-03
Akt3	-1.62	4.55E-03
Mkln1	-1.62	2.51E-04
Fam126b	-1.62	2.47E-03
Gphn	-1.62	6.97E-04
Ndufb4	-1.62	3.32E-03
Zmym6	-1.62	1.04E-04
Gimap3	-1.62	2.73E-03
Mtcp1	-1.62	3.24E-04
Ephx4	-1.62	1.66E-02
Cpne3	-1.62	4.09E-03
Slc10a7	-1.62	1.97E-04
Tmem71	-1.62	7.72E-03
Sdha	-1.63	4.92E-04
Fam69a	-1.63	2.46E-03
Elp4	-1.63	6.90E-03
Thoc1	-1.63	2.04E-03
Elmod2	-1.63	1.55E-02
Rnf170	-1.63	2.23E-05
Ankmy2	-1.63	1.78E-04
Gtf2a2	-1.63	3.53E-03
Ireb2	-1.63	3.79E-03
Smurf2	-1.63	3.61E-03
Lamp1	-1.63	5.69E-03
Zfp110	-1.63	2.17E-04

10441633	Ccr6	NM_009835	8.20E-05
10455961	Iigp1	NM_001146275	8.22E-05
10462973	Hells	NM_008234	8.23E-05
10457118	Rttn	NM_175542	8.30E-05
10461587	Ms4a4a	XM_889011	8.44E-05
10358754	Gm7278	XR_034437	8.49E-05
10364373	Lsm7	NM_025349	8.51E-05
10537657	Ephb6	NM_001146351	8.52E-05
10492815	Tmem154	NM_177260	8.53E-05
10454286	Mapre2	NM_153058	8.54E-05
10381668	Nmt1	NM_008707	8.60E-05
10567343	2310008H09Rik	NM_023197	8.75E-05
10364856	Dot1l	NM_199322	8.76E-05
10338321		---	8.77E-05
10475517	AA467197	ENSMUST000000	8.77E-05
10394978	Rrm2	NM_009104	8.78E-05
10532085	Tgfbr3	NM_011578	8.84E-05
10591658	LOC100049077	AK014010	8.84E-05
10495594		---	8.84E-05
10555894	Dub1	NM_007887	8.86E-05
10474243	Cstf3	NM_145529	8.87E-05
10375103	Fbxw11	NM_134015	8.93E-05
10384493	Gapdh	NM_008084	8.95E-05
10396367	Six6	NM_011384	9.07E-05
10395807	1110008L16Rik	NM_025373	9.08E-05
10451061	Runx2	NM_001146038	9.11E-05
10555256	Gm10605	ENSMUST000000	9.14E-05
10538138	Gimap1	NM_008376	9.16E-05
10595718	Chst2	NM_018763	9.27E-05
10385175	Wwc1	NM_170779	9.33E-05
10503334	Gem	NM_010276	9.37E-05
10341250		---	9.37E-05
10389134	Slfn9	NM_172796	9.43E-05
10404323	Prl2a1	NM_019991	9.45E-05
10355628	Rnf25	NM_021313	9.55E-05
10555695	Rrm1	NM_009103	9.60E-05
10357332	Actr3	NM_023735	9.61E-05
10592201	Chek1	NM_007691	9.64E-05
10493850	Sprr2a	NM_011468	9.66E-05
10345509	Zap70	NM_009539	9.69E-05
10450365		---	9.71E-05
10388430	Serpinf1	NM_011340	9.73E-05
10574511	BC015286	ENSMUST000000	9.77E-05
10588083	Faim	NM_001122851	9.82E-05
10457385	Ccny	NM_026484	9.83E-05
10503186	Chd7	NM_001081417	9.97E-05
10607870	Tlr7	NM_133211	9.97E-05
10422321	Dzip1	NM_025943	0.00010004
10442495	Pkd1	NM_013630	0.00010007
10462697		---	0.0001004
10571958	Sh3rf1	NM_021506	0.00010045
10530819	Hopx	NM_175606	0.00010075
10548143	Gapdh	NM_008084	0.00010082
10344498		---	0.00010171
10426751	Tmbim6	NM_026669	0.00010186
10535866	Ubl3	NM_011908	0.00010226
10359405	Klhl20	NM_001039482	0.00010234
10548735	Dusp16	NM_130447	0.0001026
10371002	Lsm7	NM_025349	0.00010262
10467560	Tm9sf3	NM_133352	0.00010298
10363498	Ppa1	NM_026438	0.00010363
10469695	Apbb1ip	NM_019456	0.00010562
10341783		---	0.00010577
10550029	Zfp110	NM_022981	0.00010599
10404059	Hist1h1c	NM_015786	0.00010713
10353064	Arfgef1	NM_001102430	0.00010755
10596072	Ppp2r3a	NM_001161362	0.00010815
10344719	Gm7075	ENSMUST000000	0.0001099
10538704		---	0.00011
10399768	Ttc15	NM_178811	0.00011093
10608650		---	0.00011167
10365208	Zfp873	NM_001024626	0.00011244
10341739		---	0.0001127
10487748	4930402H24Rik	BC052447	0.00011307
10346668	Fam117b	NM_001037725	0.00011307
10363786	Ank3	NM_146005	0.00011361
10573008	Zfp827	NM_178267	0.00011373
10600485	Dkc1	NM_001030307	0.00011376
10580391	Itfg1	NM_028007	0.0001145
10571876	Gapdh	NM_008084	0.0001147
10401454	Entpd5	NM_001026214	0.00011479
10356936	Hisppd1	NM_173760	0.0001153
10456519		---	0.00011542
10415960	Ints9	NM_153414	0.00011566
10591556	Spc24	NM_026282	0.0001176
10543080	Rnps1	NM_009070	0.00011767
10549171	5730419I09Rik	NM_029081	0.00011769
10338467		---	0.00011786
10412830	Ngly1	NM_021504	0.00011802
10439845	Gm5486	ENSMUST000000	0.0001181
10358816	Lamc1	NM_010683	0.00011821
10392183	Ern1	NM_023913	0.00011824
10503431	Nbn	NM_013752	0.00011825
10455780	Gapdh	NM_008084	0.00011849
10454655	Apc	NM_007462	0.00011915
10399005	Crip1	NM_007763	0.00012027
10444016	Pram1	NM_001002842	0.00012041
10550167		---	0.00012064
10501051	Cept1	NM_133869	0.00012168
10473384	Slc43a3	NM_021398	0.00012202
10341290		---	0.0001223
10454786	Ctnna1	NM_009818	0.00012295
10365933	Eea1	NM_001001932	0.00012362
10574524	Ces2	NM_145603	0.00012396
10411508	Ptcd2	NM_026873	0.00012431
10342839		---	0.00012438
10558248	Bub3	NM_009774	0.00012518
10497135	Rpe65	NM_029987	0.00012563
10339607		---	0.00012594
10519324	Cdk6	NM_009873	0.00012654
10505445	Orm3	NM_013623	0.00012729
10382243	Gna13	NM_010303	0.0001281
10338530		---	0.00012819
10421526	Rb1	NM_009029	0.00012829
10500122	Bnipl	NM_134253	0.00012859
10374500	Vps54	NM_139061	0.00012868
10600034	Pasd1	NR_027801	0.00012873
10497503	Kpna2	NM_010655	0.00012876
10453459	Pnkd	NM_025580	0.00012933
10361712	Shprh	NM_172937	0.00012942
10564667	Ntrk3	NM_008746	0.00012964
10586242	Dennd4a	NM_001162917	0.00013
10493120	Gpatch4	NM_025663	0.00013074
10542040	Parp11	NM_181402	0.00013096
10531261	Rassf6	NM_028478	0.00013118
10472621	Ubr3	NM_001081548	0.00013159
10402585	Wars	NM_011710	0.00013233
10365056	Apba3	NM_018758	0.00013253

Ube3c	-1.63	1.09E-04
Capn7	-1.63	3.50E-03
St6galnac3	-1.63	9.67E-05
Ddx50	-1.63	1.48E-02
St7	-1.64	1.77E-02
2210012G02Rik	-1.64	3.89E-03
Dnajc15	-1.64	3.56E-03
Fbxl17	-1.64	5.97E-05
Polr2g	-1.64	7.42E-03
Atpbd4	-1.64	1.70E-05
2310037I24Rik	-1.64	6.02E-04
Mcm9	-1.64	1.02E-02
Scai	-1.64	6.02E-04
Usp45	-1.64	3.61E-04
Lrmp	-1.64	5.21E-03
Vps13c	-1.64	2.82E-04
Rbbp6	-1.65	1.12E-02
Fam48a	-1.65	1.10E-03
Ppih	-1.65	1.01E-03
Tmtc4	-1.65	7.20E-03
Inadl	-1.65	1.01E-02
Snx4	-1.65	5.48E-03
2810021B07Rik	-1.65	4.34E-03
Mrpl37	-1.65	9.18E-03
Tmem185b	-1.65	6.74E-03
Naa35	-1.65	4.20E-03
Shc1	-1.65	5.28E-03
Pikfyve	-1.65	1.36E-06
Ints9	-1.65	3.87E-04
Atf2	-1.65	1.23E-02
Pibf1	-1.66	1.78E-04
Enox2	-1.66	5.41E-04
Vwa5a	-1.66	4.78E-04
Disp1	-1.66	2.84E-04
Ap4e1	-1.66	1.46E-02
Adam10	-1.66	6.35E-05
Zfp563	-1.66	7.36E-03
4632404H12Rik	-1.66	2.01E-03
0610037L13Rik	-1.66	2.75E-03
Cryzl1	-1.66	1.50E-02
Zfp125	-1.66	2.52E-03
Atp5e	-1.66	3.26E-04
Rnf213	-1.66	4.62E-03
Taf2	-1.66	5.21E-04
Acat1	-1.66	3.74E-05
Nup107	-1.66	3.76E-03
D19Bwg1357e	-1.66	7.97E-04
Themis	-1.67	1.77E-03
Extl3	-1.67	4.50E-04
Ngly1	-1.67	3.19E-05
Zfp273	-1.67	3.57E-03
Gnaq	-1.67	1.43E-02
Med8	-1.67	1.63E-04
Ube2v2	-1.67	9.88E-05
2810474O19Rik	-1.67	3.26E-04
Pcf11	-1.67	3.45E-03
Hgf	-1.67	2.43E-04
Prdx2	-1.67	1.09E-02
Eif2s1	-1.67	7.15E-03
Tgs1	-1.67	1.42E-02
2010002M12Rik	-1.67	6.37E-04
Trim24	-1.67	9.14E-03
Ppil4	-1.67	1.33E-02
Cwc22	-1.67	3.51E-05
Gm6644	-1.68	1.72E-03
Mettl2	-1.68	3.72E-03
Ppip5k2	-1.68	1.21E-03
Mrpl48	-1.68	1.17E-02
Alg6	-1.68	1.62E-04
Zdhhc17	-1.68	1.05E-02
Ipcef1	-1.68	7.35E-03
Mitd1	-1.68	1.55E-02
5033414D02Rik	-1.68	5.95E-04
1110002N22Rik	-1.68	5.99E-04
2810408P10Rik	-1.68	1.58E-02
Zfp810	-1.68	5.84E-04
Mett5d1	-1.68	9.37E-06
Pdxdc1	-1.68	3.56E-03
Zfp87	-1.68	5.98E-03
Ankhd1	-1.68	1.19E-02
Pram1	-1.68	1.76E-04
Farsb	-1.68	5.48E-03
Ap1s2	-1.69	1.51E-02
Ube2r2	-1.69	2.77E-03
Esf1	-1.69	2.49E-03
4930522L14Rik	-1.69	1.52E-03
Ilk	-1.69	1.15E-04
Dnajc10	-1.69	3.72E-03
Oma1	-1.69	1.06E-03
Pyhin1	-1.69	1.30E-03
Ly9	-1.69	1.03E-04
Alkbh8	-1.69	3.99E-05
Ttc3	-1.69	2.85E-04
6330409N04Rik	-1.69	1.21E-02
Adam1a	-1.69	2.92E-03
Parp14	-1.69	2.69E-04
Nsmce2	-1.69	8.64E-03
Msh3	-1.70	1.62E-07
AW146020	-1.70	1.53E-02
Atr	-1.70	2.85E-04
Lyrm2	-1.70	7.18E-03
Sag	-1.70	2.00E-03
Rtn4ip1	-1.70	2.42E-03
Vav1	-1.70	1.63E-03
Uba3	-1.70	1.32E-02
Cd3d	-1.70	2.52E-03
Rab3gap1	-1.70	3.06E-04
Nae1	-1.71	1.92E-03
Snap29	-1.71	1.21E-02
Cwf19l2	-1.71	2.14E-03
Anapc4	-1.71	1.73E-02
Plekha8	-1.71	3.79E-03
Pdcd4	-1.71	1.98E-03
Usp18	-1.71	6.71E-04
D930015E06Rik	-1.71	7.32E-03
Slc44a2	-1.71	1.30E-02
Lpar6	-1.71	1.89E-03
Rpl31	-1.71	2.58E-03
Gimap1	-1.71	1.47E-04
Gm10726	-1.71	1.04E-02
Lysmd1	-1.71	1.18E-03
Magohb	-1.71	5.63E-04
Zfml	-1.72	2.32E-04
Csnk2a1	-1.72	1.39E-03
Stk39	-1.72	5.25E-05
Nedd9	-1.72	1.57E-04
Ppp1r11	-1.72	7.41E-03
Zfp599	-1.72	1.75E-02

10401931		---	0.00013313
10401937	BC005685	BC005685	0.00013313
10442331	Pkmyt1	NM_023058	0.00013369
10540191	Nr2c2	NM_011630	0.00013394
10520304	Actr3b	NM_001004365	0.00013431
10376269	Galnt10	NM_134189	0.00013437
10492658	Gm5523	NR_004447	0.00013491
10417095	Farp1	NM_134082	0.00013589
10435470	Kpna1	NM_008465	0.00013593
10504751		---	0.00013597
10545130	Gadd45a	NM_007836	0.000136
10357436	Mcm6	NM_008567	0.00013628
10571054	Star	NM_011485	0.00013636
10429385		---	0.00013754
10576661	Itgb1	NM_010578	0.00013761
10355567	Tmbim1	NM_027154	0.00013876
10557434	Apob48r	NM_138310	0.00013932
10405969		---	0.00013933
10418729	Colq	NM_009937	0.00014029
10436369	Filip1l	NM_030163	0.00014077
10344414		---	0.00014139
10435907	Cd200r1	NM_021325	0.0001414
10522303	Guf1	NM_172711	0.00014191
10468980	Fam107b	BC021353	0.00014236
10600825	Zc3h12b	NM_001034907	0.00014277
10406417	Actg1	NM_009609	0.00014304
10450369	Hspa1a	NM_010479	0.00014448
10549569	Cnot3	NM_146176	0.0001445
10466843	Gapdh	BC092294	0.00014464
10608646		---	0.00014473
10420877	Esco2	NM_028039	0.00014481
10436106	C330027C09Rik	NM_172616	0.00014605
10599717		---	0.0001469
10422161	Gapdh	NM_008084	0.00014737
10414427	Gm6498	NR_003630	0.00014746
10562546		---	0.00014779
10427742	Gm8174	XR_001896	0.00014828
10571870	Hmgb2	NM_008252	0.00014847
10354832	Ppil3	NM_027351	0.00014859
10372069	Socs2	NM_007706	0.00014883
10563883	Depdc1a	NM_029523	0.00014894
10394770	Odc1	NM_013614	0.00014912
10439249	Parp14	NM_001039530	0.00014915
10406968	Cenpk	NM_021790	0.00014916
10343720		---	0.00015033
10444658	Clic1	NM_033444	0.00015042
10514133	Ttc39b	NM_027238	0.00015103
10462866	Cep55	NM_028760	0.00015154
10440488	Gm311	XM_001473851	0.00015202
10341730		---	0.00015286
10525665	Ccdc62	NM_001134767	0.00015306
10452415	Gapdh	M32599	0.0001535
10467489	Ptp4a1	NM_011200	0.00015351
10360158	Ly9	NM_008534	0.00015582
10565921	Gapdh	NM_008084	0.00015634
10487340	Ncaph	NM_144818	0.0001574
10530163	Rfc1	NM_011258	0.00015811
10450367	Hspa1a	NM_010479	0.00015814
10415662	Rcbtb1	NM_027764	0.00015828
10352066	Sdccag8	NM_029756	0.00015844
10345445	Arid5a	NM_145996	0.00015961
10342782		---	0.00016154
10372001		---	0.00016201
10602599	Smc1a	NM_019710	0.00016294
10438220	Car15	NM_030558	0.00016344
10588049	Copb2	NM_015827	0.0001636
10505438	Orm1	NM_008768	0.00016461
10404538	Prpf4b	NM_013830	0.0001652
10422028	Tbc1d4	NM_001081278	0.00016529
10596769	Rbm5	NM_148930	0.00016541
10433264	Glis2	NM_031184	0.0001656
10405733	6720457D02Rik	NM_175252	0.0001659
10361710	Gm5177	AK135747	0.00016624
10528167	Gapdh	NM_008084	0.00016716
10565018	Iqgap1	NM_016721	0.00016716
10541484	M6pr	NM_010749	0.00016765
10504551	Rg9mtd3	NM_027266	0.00016783
10544415		---	0.00016849
10463704	As3mt	NM_020577	0.00016861
10346790	Ctla4	NM_009843	0.00016898
10538850		---	0.00017133
10515939	Foxo6	NM_194060	0.00017168
10565532	Ankrd42	NM_028665	0.00017175
10392284	Kpna2	NM_010655	0.00017204
10488944	Uqcc	NR_024487	0.00017219
10550574	Dmpk	NM_032418	0.00017248
10441436	Snx9	NM_025664	0.00017273
10393823	P4hb	NM_011032	0.00017449
10374197	Ramp3	NM_019511	0.00017456
10608667		---	0.0001747
10348713	E030010N08Rik	AK086911	0.00017525
10467230	Ide	NM_031156	0.00017535
10598091		---	0.00017571
10366337	Nap1l1	NM_001146707	0.00017586
10491300	Skil	NM_011386	0.00017621
10422655	Gapdh	BC092294	0.0001763
10465831	5730408K05Rik	NR_027866	0.00017788
10412345	Parp8	NM_001081009	0.00017861
10393573	Lgals3bp	NM_011150	0.00017894
10604333	Decaf12l1	NM_178739	0.00017913
10547410	Erc1	NM_053204	0.00017947
10408280	Lrrc16a	NM_026825	0.00017983
10518585	Kif1b	NM_207682	0.00018037
10439527	Tigit	NM_001146325	0.0001804
10439798	Dzip3	NM_001110017	0.00018061
10339082		---	0.00018098
10383206		---	0.0001816
10557156	Plk1	NM_011121	0.00018189
10454369	Fhod3	NM_175276	0.00018197
10433721	Nde1	NM_023317	0.00018229
10463064	Gapdh	NM_008084	0.0001826
10375439	Med7	NM_025426	0.00018283
10517715	Pax7	NM_011039	0.00018336
10342623		---	0.00018412
10533304	Trafd1	NM_172275	0.00018458
10439895	Alcam	NM_009655	0.00018675
10478890	Cebpb	NM_009883	0.00018743
10578149	Leprotl1	NM_026609	0.00018774
10477073	Csnk2a1	NM_007788	0.00018864
10535647	Atp5j2	NM_020582	0.00018891
10515558	Artn	NM_009711	0.00018974
10396237	2700049A03Rik	BC150744	0.0001902
10473965	Arhgap1	NR_027373	0.00019032
10594762	Fam81a	BC025646	0.00019044
10601567	Gapdh	BC092294	0.00019093
10605493	Prrg1	BC032926	0.00019099
10454198	Rnf125	NM_026301	0.00019246
10340904		---	0.00019376

Xk	-1.72	9.25E-03
Pign	-1.72	1.63E-03
Zfp709	-1.72	2.00E-03
Cdipt	-1.72	2.17E-05
Nit2	-1.72	1.67E-02
Hsd17b4	-1.72	5.08E-03
C920016K16Rik	-1.72	1.39E-02
Eif2ak4	-1.72	1.23E-03
Senp7	-1.73	2.99E-03
Nlk	-1.73	3.29E-03
Znrf2	-1.73	1.03E-02
Ap1g1	-1.73	2.76E-05
Gm5887	-1.73	1.28E-04
Stard7	-1.73	1.00E-02
Carf	-1.73	4.54E-04
Rad17	-1.73	1.56E-02
3830406C13Rik	-1.74	8.85E-04
Chn2	-1.74	1.64E-04
Zfp329	-1.74	1.02E-03
Brms1l	-1.74	5.95E-03
Rnf25	-1.74	6.07E-05
Poldip3	-1.74	8.84E-04
Hint3	-1.74	7.37E-03
Ltn1	-1.74	1.02E-03
Ppa2	-1.74	5.98E-04
Vav3	-1.74	9.33E-03
Vps13a	-1.75	4.04E-03
Nol11	-1.75	7.32E-04
Nudcd1	-1.75	9.95E-03
Tspan32	-1.75	1.18E-02
Nme7	-1.75	1.30E-03
Mut	-1.75	7.19E-03
Ccdc62	-1.75	6.42E-04
6330416L07Rik	-1.75	2.39E-05
Slc28a2	-1.75	7.34E-04
Thada	-1.75	9.16E-04
Bbs12	-1.75	1.12E-04
Zfp715	-1.75	6.57E-03
Zfp397	-1.75	1.35E-04
Ddx47	-1.75	1.08E-02
Mtif2	-1.75	1.58E-04
Mettl14	-1.76	6.19E-03
Orc2	-1.76	6.64E-03
Fastkd2	-1.76	5.38E-04
C330018D20Rik	-1.76	1.51E-03
Ccnyl1	-1.76	3.57E-03
Ccdc43	-1.76	3.47E-03
Acsl5	-1.76	1.14E-03
Hivep2	-1.76	1.60E-03
Atrn	-1.76	4.78E-07
D3Ertd751e	-1.76	7.57E-03
3110052M02Rik	-1.76	8.38E-03
Atp11c	-1.76	4.05E-04
Bod1l	-1.76	4.02E-03
Zfp825	-1.76	3.07E-03
Mpp1	-1.76	2.89E-03
Ric8b	-1.77	1.18E-02
Zfp143	-1.77	5.53E-04
Cdc14b	-1.77	1.45E-03
Ctsc	-1.77	6.92E-03
Psmd6	-1.77	1.54E-02
Rpl30	-1.77	2.79E-03
Abce1	-1.77	8.77E-03
8030462N17Rik	-1.77	2.43E-03
Wdr36	-1.77	3.90E-03
Prkag2	-1.78	3.09E-04
Gng5	-1.78	6.86E-03
Slfn8	-1.78	5.88E-04
Tgtp1	-1.78	6.47E-03
Guf1	-1.78	2.45E-04
Immp1l	-1.78	8.88E-03
Slfn5	-1.78	8.03E-04
Zfp39	-1.78	1.55E-04
Lsm3	-1.78	6.15E-03
Cab39l	-1.78	1.52E-04
Exd2	-1.79	3.65E-03
Ttc39b	-1.79	8.43E-04
Usp32	-1.79	9.52E-05
Slc25a40	-1.79	1.32E-02
Mlh3	-1.79	5.73E-04
Scoc	-1.79	9.80E-04
Taf9	-1.79	8.36E-03
Zfp788	-1.79	4.95E-03
Lipo1	-1.80	1.56E-02
Dgcr8	-1.80	9.37E-03
Agps	-1.80	9.40E-03
Chic1	-1.80	4.17E-03
0610010B08Rik	-1.80	4.79E-03
Gm14430	-1.80	4.79E-03
Cetn3	-1.80	1.07E-03
Gbp1	-1.80	2.91E-03
Gm5665	-1.80	1.02E-02
6720456H20Rik	-1.80	4.00E-04
Opa1	-1.81	1.71E-02
Slamf7	-1.81	1.22E-03
Ankrd12	-1.81	1.62E-02
Dkc1	-1.81	2.09E-04
Ap4s1	-1.81	1.61E-03
Nsmaf	-1.81	5.37E-03
Tubgcp3	-1.81	5.31E-04
Rp2h	-1.81	2.68E-03
Pion	-1.81	5.68E-05
Slc12a6	-1.82	1.19E-03
Zfp827	-1.82	3.18E-04
Ptger2	-1.82	6.40E-04
Pot1b	-1.82	9.69E-04
Supt16h	-1.82	2.63E-03
Zfp738	-1.82	4.31E-03
Snora3	-1.82	1.45E-03
Rcn1	-1.83	5.86E-03
Ankrd44	-1.83	1.09E-03
Prkdc	-1.83	4.75E-04
Eftud1	-1.83	3.54E-05
Prmt3	-1.83	2.71E-03
Apc	-1.83	2.48E-04
Atf7ip	-1.83	1.98E-03
Pdk1	-1.83	1.82E-03
4933411K20Rik	-1.84	4.64E-03
Brd7	-1.84	1.70E-03
Zfp386	-1.84	4.04E-04
0610007P08Rik	-1.84	7.08E-03
Mina	-1.84	4.50E-03
Fv1	-1.84	1.08E-02
Heatr5b	-1.84	3.74E-03
Gm14326	-1.84	3.04E-03
Mepce	-1.84	7.87E-03
D14Abb1e	-1.84	2.13E-03
Adal	-1.85	8.57E-03

10446074	Uhrf1	NM_010931	0.000194
10428302	Klf10	NM_013692	0.00019529
10525086	Slc24a6	NM_133221	0.00019566
10559207	Lsp1	NM_019391	0.00019695
10467739	Avpi1	NM_027106	0.00019869
10414001	Gm626	XM_985917	0.00020169
10414433	6720456H20Rik	NM_172600	0.000202
10602454	Fam120c	NM_198105	0.00020205
10441195	Dscam	NM_031174	0.00020216
10399038	Zfp386	NM_001004066	0.00020237
10564960	Furin	NM_011046	0.0002026
10476319		---	0.00020266
10570434	Ifitm1	NM_026820	0.00020295
10574421	Gm10632	ENSMUST00000(	0.00020412
10605919	Pja1	NM_001083110	0.00020501
10418506	Stab1	NM_138672	0.00020504
10586254	Dennd4a	NM_001162917	0.00020507
10405013	Ippk	NM_199056	0.00020509
10405263	4732471D19Rik	NM_176987	0.00020539
10358670	Hmcn1	NM_001024720	0.00020621
10507231	Kncn	NM_001039124	0.00020646
10546163	Mcm2	NM_008564	0.0002068
10339306		---	0.00020702
10353707	Ptp4a1	NM_011200	0.00020806
10576417	Galnt2	NM_139272	0.00020826
10572095	Nat3	NM_008674	0.00020852
10399254	Cenpo	NM_134046	0.00020872
10589438	Mtap4	NM_008633	0.00020881
10582925	Alkbh8	NM_026303	0.00020987
10528523	Tomm7	NM_025394	0.00021118
10545707	Actg2	NM_009610	0.00021179
10524169	Pole	NM_011132	0.00021223
10542885	2810474O19Rik	NM_026054	0.00021239
10604451	Enox2	NM_145951	0.00021246
10503283	1110037F02Rik	BC033309	0.00021417
10399680	Cys1	NM_138686	0.00021515
10358408	Rgs1	NM_015811	0.00021614
10362861	Scml4	NM_172938	0.00021707
10538356	Chn2	NM_023543	0.0002177
10378833	Ssh2	NM_177710	0.00021818
10411452	Gapdh	M32599	0.00021895
10377018	Myh3	NM_001099635	0.00021916
10497520	Ect2	NM_007900	0.00021927
10482695	Cacnb4	NM_001037099	0.00021993
10346914	Fastkd2	NM_172422	0.00022004
10496130	A630047E20Rik	BC060502	0.00022151
10469425	Arl5b	NM_029466	0.00022225
10587051	Wdr72	NM_001033500	0.00022231
10407833	Ggps1	NM_010282	0.00022245
10508392	Rnf19b	NM_029219	0.00022292
10474902	Rad51	NM_011234	0.00022384
10363455	Pcbd1	NM_025273	0.00022417
10427235	Prr13	NM_025385	0.0002249
10503926	Rars2	NM_181406	0.00022506
10558297	2700050L05Rik	BC072596	0.00022517
10471571	Mapkap1	NM_177345	0.00022551
10587699	Rasgrf1	NM_011245	0.00022559
10422059	Kctd12	NM_177715	0.00022566
10373454	Pa2g4	NM_011119	0.00022599
10570000	Gpi1	NM_008155	0.00022635
10382998	Birc5	NM_001012273	0.00022718
10600819	Zxdb	NM_001081473	0.00022884
10409866	Ctla2b	NM_007797	0.00022888
10344821	Cspp1	NM_026493	0.00022927
10517147	Dhdds	NM_026144	0.00022986
10507594	Slc2a1	NM_011400	0.00022994
10408689	Nrn1	NM_153529	0.00023055
10482772	Nr4a2	NM_001139509	0.00023063
10464128	Casp7	NM_007611	0.00023191
10399973	Hdac9	NM_024124	0.00023239
10585699	Fabp5	NM_010634	0.0002327
10368947	Aim1	NM_172393	0.00023322
10401616	Mlh3	NM_175337	0.00023344
10339916		---	0.00023407
10443463	Cdkn1a	NM_007669	0.00023456
10439483	Cdgap	NM_020260	0.00023567
10566578	Gvin1	NM_029000	0.00023589
10338064		---	0.00023623
10483698	Wipf1	NM_153138	0.00023645
10528664	Smarcd3	NM_025891	0.00023794
10465861	Incenp	NM_016692	0.00023802
10353250	Gapdh	M32599	0.00023802
10340809		---	0.00023804
10525542	Bcl7a	NM_029850	0.00023814
10594645	Rab8b	NM_173413	0.00023873
10433797	Prkdc	NM_011159	0.00023998
10507418	Eif2b3	NM_001111277	0.00024
10588577	Cish	NM_009895	0.00024076
10390763	Ccr7	NM_007719	0.00024078
10349239	Mki67ip	NM_026472	0.00024113
10342177		---	0.00024168
10411229	F2r	NM_010169	0.00024175
10547100	Plxnd1	NM_026376	0.00024201
10547936	Gapdh	NM_008084	0.00024395
10569757	1700019B03Rik	BC050809	0.00024428
10414315	Cdkn3	BC049694	0.00024564
10350733	Rgs16	NM_011267	0.00024706
10348244	Inpp5d	NM_010566	0.00024838
10342948		---	0.00024858
10577070	Tubgcp3	NM_198031	0.00024956
10511679	Decr1	NM_026172	0.0002497
10493820	S100a6	NM_011313	0.00024993
10594251	Kif23	NM_024245	0.00025069
10518147	Pdpn	NM_010329	0.00025125
10339098		---	0.00025164
10340639		---	0.00025208
10379820	Acaca	NM_133360	0.00025449
10390319	Lrrc46	NM_027026	0.00025458
10450646	Rbx1	NM_019712	0.0002551
10451886	BC031441	BC031441	0.00025525
10468898	Lax1	NM_001159649	0.00025768
10444284	H2-Ob	NM_010389	0.00025797
10415909		---	0.00025835
10608681		---	0.00025849
10510172	Hmgb2	NM_008252	0.00025873
10512655	Rnf38	NM_001038993	0.00025878
10430945	Poldip3	NM_178627	0.0002588
10551423	4933426I21Rik	BC024577	0.00025916
10347058		---	0.00026007
10527508	Cdk8	NM_153599	0.00026026
10409876	Ctla2a	NM_007796	0.00026099
10522335	Atp10d	NR_003966	0.00026154
10462587	Stambpl1	NM_029682	0.00026326
10530974	Tmprss11a	NM_001033233	0.00026343
10519857	Hgf	NM_010427	0.00026376
10587350		---	0.00026386
10357579	Mapkapk2	NM_008551	0.00026413
10438738	Bcl6	NM_009744	0.00026507

Rspry1	-1.85	4.42E-03
A530032D15Rik	-1.85	8.14E-03
Prdm2	-1.85	5.14E-04
Apool	-1.85	3.01E-03
100043387	-1.85	6.61E-03
Znhit6	-1.85	6.45E-03
Inpp4b	-1.85	7.32E-03
Zfp456	-1.86	2.63E-03
Zfp605	-1.86	2.49E-03
Cerkl	-1.86	3.30E-04
Nanp	-1.86	5.19E-03
Yars2	-1.86	5.60E-03
Ipp	-1.86	1.37E-02
Crot	-1.86	1.85E-04
Chd9	-1.86	6.22E-05
Stard3nl	-1.87	2.59E-04
Fam26f	-1.87	3.15E-06
LOC641050	-1.87	1.50E-03
Kif1c	-1.87	6.90E-03
Glb1	-1.87	6.51E-05
Ms4a6c	-1.87	4.34E-03
Mrps33	-1.88	9.79E-05
BC094916	-1.88	1.23E-02
Tbck	-1.88	1.47E-04
Tsr1	-1.88	2.64E-04
Orc3	-1.88	3.14E-05
Zfp420	-1.89	3.98E-04
Taf1a	-1.89	1.73E-04
Copg2	-1.89	4.92E-03
Naip6	-1.89	9.53E-06
Ptpn12	-1.89	1.54E-02
Rb1cc1	-1.89	4.35E-03
Pcca	-1.89	5.58E-04
Nop10	-1.90	2.09E-03
1110001A16Rik	-1.90	9.89E-05
Txndc12	-1.90	6.73E-03
Dzip3	-1.90	8.10E-04
Tmem209	-1.90	7.17E-07
Tbc1d13	-1.90	2.32E-04
Narg2	-1.90	2.47E-03
Lrpprc	-1.91	2.12E-04
Gtpbp8	-1.91	5.32E-03
E130120F12Rik	-1.91	6.11E-05
Irak4	-1.91	1.47E-04
Ubxn2b	-1.91	7.40E-04
BC002059	-1.91	2.16E-04
Ube2w	-1.91	2.42E-03
Ptcd3	-1.92	2.68E-03
Prkca	-1.92	1.90E-04
Tomm5	-1.92	3.08E-03
1700034H14Rik	-1.92	1.20E-05
Polr2l	-1.93	5.61E-03
Card6	-1.93	9.87E-04
2310001H12Rik	-1.93	2.61E-03
Agk	-1.93	7.78E-04
Imp3	-1.93	4.94E-03
Tmem161b	-1.94	7.16E-03
Aqr	-1.94	1.33E-03
Zfp763	-1.94	8.69E-04
Gm9938	-1.94	5.21E-03
Slc9a9	-1.94	1.05E-04
Thap2	-1.94	6.19E-03
Arap2	-1.94	1.43E-02
Zfp826	-1.95	3.21E-03
Snhg1	-1.95	4.14E-03
ND3	-1.95	1.53E-03
Ptpn4	-1.96	7.08E-04
A530054K11Rik	-1.96	1.53E-02
Gpatch8	-1.96	8.69E-03
Nt5dc1	-1.96	4.99E-03
Slfn2	-1.97	1.75E-04
BC005685	-1.98	1.08E-04
Casd1	-1.98	7.99E-03
Zufsp	-1.98	1.04E-02
Prkaa1	-1.98	2.71E-03
Zfp119b	-1.98	3.66E-04
Znrd1as	-1.99	1.04E-03
Nlrc5	-2.00	4.81E-03
Abcg3	-2.00	2.31E-03
Lypla1	-2.00	1.62E-03
Dram2	-2.01	2.07E-03
2610044O15Rik	-2.01	9.13E-03
Klhl20	-2.01	1.61E-04
Zfp558	-2.01	6.61E-03
Art2a-ps	-2.01	2.48E-03
1110008F13Rik	-2.02	1.44E-04
Haus1	-2.02	3.09E-04
Ccdc125	-2.03	2.20E-03
Decr1	-2.03	4.90E-04
Ddx60	-2.03	2.60E-03
Rps6ka3	-2.03	6.03E-03
Mtx2	-2.03	9.34E-06
0610007P08Rik	-2.03	3.61E-07
Zfp781	-2.05	5.24E-05
Glmn	-2.05	4.78E-03
Nrp1	-2.05	1.85E-03
Smarcad1	-2.05	6.73E-03
Phkb	-2.05	8.14E-05
Tmem229b	-2.05	3.31E-03
Actr6	-2.06	8.21E-03
Eif2ak2	-2.06	1.82E-03
Rpp30	-2.06	9.03E-04
Exoc6	-2.06	1.97E-03
Gm10336	-2.06	1.92E-03
Ccdc90b	-2.07	5.57E-03
D6Wsu163e	-2.07	2.22E-06
Atm	-2.08	4.13E-04
Gm10688	-2.09	5.02E-04
Zfp182	-2.10	9.39E-03
Armc8	-2.10	1.25E-02
Poglut1	-2.10	4.61E-03
Ttc13	-2.11	1.33E-05
Utp15	-2.11	1.81E-04
Arhgap15	-2.11	1.87E-03
Zfp870	-2.11	3.63E-03
Fam82b	-2.11	1.81E-04
Trim30d	-2.12	2.87E-03
Gm701	-2.12	3.94E-03
Tlr1	-2.12	3.78E-03
Zfp595	-2.13	1.66E-04
Sidt1	-2.13	9.25E-03
Rbbp6	-2.13	8.09E-03
Cd46	-2.14	9.18E-03
Zfp955a	-2.15	3.03E-04
Cstf3	-2.15	1.99E-04
Zmat1	-2.15	6.87E-03
Gm13892	-2.15	1.45E-03
Tbl1xr1	-2.15	6.72E-03

10471535	Fam129b	NM_146119	0.00026598
10379034	Tlcd1	NM_026708	0.00026703
10545534	Rnf26	NM_153762	0.00026737
10393754	Actg1	NM_009609	0.00026824
10501048	2010016I18Rik	AK140363	0.00026871
10598034	ND2	ENSMUST00000(	0.0002689
10456492	D18Ertd653e	BC096371	0.00026987
10566398	Olfr666	NM_147096	0.00027
10474825	D2Ertd750e	NM_026412	0.00027054
10578145	Erh	NM_007951	0.00027172
10603289	Clcn5	NM_016691	0.0002723
10505911	Dmrta1	NM_175647	0.00027288
10478633	Mmp9	NM_013599	0.00027308
10585428	Dnaja4	NM_021422	0.00027736
10487480	Bub1	NM_001113179	0.00027804
10369615	Srgn	NM_011157	0.00027816
10582562		---	0.00027824
10521205	Sh3bp2	NM_001145859	0.0002784
10400892	Gm7985	XR_034366	0.00028048
10482863	Gm13549	XR_031802	0.00028063
10396740	Gphn	NM_172952	0.00028218
10407173	Il6st	NM_010560	0.00028278
10410625	Sdha	NM_023281	0.00028303
10343272		---	0.00028381
10386219	Zfp39	NM_011758	0.00028395
10421717	Gm1587	NM_001033440	0.0002844
10462398	Pdcd1lg2	NM_021396	0.00028532
10452430	Fbxl17	BC138330	0.00028666
10565990	Art2a	NM_007490	0.00028697
10451943	Plin5	NM_001077348	0.00028759
10361110	Dtl	NM_029766	0.00028923
10513158	Ptpn3	NM_011207	0.00028956
10424543	Wisp1	NM_018865	0.00029068
10422194	Rbm26	NM_134077	0.00029069
10512574	Gba2	NM_172692	0.00029141
10396862	Strm	AF031663	0.00029187
10516246	Cdca8	NM_026560	0.00029188
10558740	Gm15542	XM_001480612	0.00029189
10417617	Gapdh	NM_008084	0.00029265
10362450	Trdn	NM_029726	0.00029359
10374323	Zpbp	NM_015785	0.00029373
10490150	Zbp1	NM_021394	0.0002942
10565994	Art2b	NM_019915	0.00029437
10395376	Ankmy2	NM_146033	0.00029477
10364130	Zfp280b	NM_177475	0.00029495
10496447	Adh6a	NM_026945	0.00029519
10474239	Gapdh	BC092294	0.00029549
10444589	Hspa1a	NM_010479	0.0002961
10501063	Cd53	NM_007651	0.00029672
10385484	Ddx5	NM_007840	0.00029767
10435565	Hcls1	NM_008225	0.0002977
10385159	Rars	NM_025936	0.0002993
10439208	Sec22a	NM_133704	0.00029937
10505674	Cntln	NM_175275	0.00030079
10512701	Tomm5	NM_001099675	0.00030131
10532164	Atp5k	NM_007507	0.00030195
10600482		---	0.00030244
10466127	AW112010	EF660528	0.00030691
10486396	Ehd4	NM_133838	0.00030762
10572235	Lpar2	NM_020028	0.00030813
10396068	Ppil5	NM_001081406	0.00030837
10338392		---	0.00030904
10357788	Ppp1r15b	NM_133819	0.00030972
10417004	Dzip1	NM_025943	0.0003098
10480432	Mastl	NM_025979	0.00031029
10543785	AB041803	AB041803	0.00031063
10354588	Stk17b	NM_133810	0.00031167
10367379	Si	NM_021882	0.00031242
10567903	Sh2b1	NM_011363	0.00031325
10459755	2810433K01Rik	NM_025581	0.00031379
10381588	Grn	NM_008175	0.0003159
10550365	Prkd2	NM_178900	0.00031643
10474875	Casc5	NM_029617	0.0003169
10425161	Lgals1	NM_008495	0.0003182
10448202	Tpm4	NM_001001491	0.00031821
10420837	Extl3	NM_018788	0.00031825
10412260	Fst	NM_008046	0.0003183
10453512	Kpna2	NM_010655	0.00031863
10580590	Gapdh	NM_008084	0.00031878
10592303	Robo3	AF060570	0.00032033
10432986	Aaas	NM_153416	0.0003209
10436830	Ifnar2	NM_010509	0.00032106
10469255	Prkcq	NM_008859	0.00032237
10603346	Plp2	NM_019755	0.0003224
10465770		---	0.00032268
10508228	Zmym6	NM_177462	0.00032328
10545651	Ino80b	NM_023547	0.00032359
10367499	Olfr788	NM_146551	0.00032391
10353010	Mybl1	NM_008651	0.00032475
10574415	1700047G07Rik	ENSMUST00000(	0.000325
10437852	4921513D23Rik	NM_001081154	0.00032569
10446777	Ehd3	NM_020578	0.00032687
10372844	Rassf3	NM_138956	0.00032798
10339709		---	0.00032829
10356001	Cul3	NM_016716	0.00032938
10543118	Glcci1	NM_133236	0.00033
10483264	Ttc21b	NM_001047604	0.00033012
10568217	Dctpp1	NM_023203	0.00033073
10584821	Cd3d	NM_013487	0.00033156
10442468	Caskin1	NM_027937	0.00033193
10497622	Lrrc34	NM_027941	0.00033336
10340521		---	0.00033379
10338116		---	0.00033396
10483856	Prkra	NM_011871	0.00033412
10562663	Rps4x	NM_009094	0.00033466
10582295	Odc1	NM_013614	0.00033532
10511692	Nbn	NM_013752	0.00033532
10414793	C920008G01Rik	ENSMUST000001	0.00033597
10414903	C920008G01Rik	ENSMUST000001	0.00033597
10427290	Hoxc8	NM_010466	0.00033669
10443749	Ubash3a	NM_177823	0.00033825
10340654		---	0.0003383
10524353	Tfip11	NM_018783	0.00034056
10359525	Bat2d	NM_001081290	0.00034391
10496756	Mcoln3	NM_134160	0.00034392
10561474	Il28a	NM_001024673	0.00034481
10570982	Fgfr1	NM_010206	0.00034505
10354307	Txn1	NM_011660	0.00034518
10455954	Gm4951	NM_001033767	0.00034609
10429560	Ly6i	NM_020498	0.00034633
10338104		---	0.00034641
10434925	Hes1	NM_008235	0.00034911
10360695	Nvl	NM_026171	0.00034914
10395365	Agr2	NM_011783	0.00034952
10406663	Arsb	NM_009712	0.00035035
10480027	Zbtb2	NM_001033466	0.00035086
10343422		---	0.00035112
10445119	H2-M3	NM_013819	0.00035173

Pnpt1	-2.16	3.60E-03
Zfp597	-2.16	5.40E-04
Hibch	-2.16	2.08E-03
Kdm5d	-2.16	7.65E-04
Pmm2	-2.17	6.49E-04
P2rx7	-2.17	8.07E-05
Dnajc3	-2.18	4.23E-03
4930432O21Rik	-2.18	2.36E-04
Ppih	-2.18	3.60E-05
Rsl1	-2.18	1.81E-03
Ephx1	-2.19	1.74E-02
Pms1	-2.21	6.60E-05
Mudeng	-2.21	1.07E-03
Ddx43	-2.21	1.73E-04
Cenpq	-2.21	3.30E-03
Vta1	-2.21	6.22E-03
Msh2	-2.21	2.51E-03
Cbwd1	-2.22	1.69E-02
Zfp759	-2.23	8.25E-03
BC018101	-2.23	1.69E-03
Fnta	-2.24	1.62E-04
Zfp81	-2.24	4.63E-03
2410002O22Rik	-2.25	7.41E-06
Ifih1	-2.26	6.28E-05
Pfdn4	-2.26	1.07E-02
Uqcc	-2.27	6.27E-05
Gpr89	-2.27	1.18E-03
Dcaf17	-2.27	1.73E-03
Snord16a	-2.27	3.50E-03
Zfp42	-2.29	1.43E-03
Rnf213	-2.29	1.69E-03
Tgds	-2.29	6.15E-03
Thumpd3	-2.29	5.99E-03
Prkcq	-2.30	5.45E-04
Dnahc8	-2.30	8.04E-04
E030037K03Rik	-2.32	9.68E-05
Zfp809	-2.33	5.33E-03
Mettl4	-2.35	3.45E-03
Taf9b	-2.35	8.56E-03
Zfp719	-2.35	9.48E-05
Gm10673	-2.35	1.44E-02
Mir421	-2.36	1.25E-03
Rrs1	-2.37	1.18E-02
Manea	-2.39	8.60E-04
5730455O13Rik	-2.40	4.22E-03
Gimap5	-2.40	1.33E-05
Sp140	-2.42	8.31E-03
Zfp160	-2.42	5.13E-03
1110057K04Rik	-2.42	6.52E-03
Mipol1	-2.44	8.64E-04
Ly6i	-2.44	5.58E-04
Parp11	-2.45	4.35E-04
Tmem19	-2.45	1.07E-02
Fam105a	-2.48	3.13E-03
9230105E10Rik	-2.48	2.58E-04
AA987161	-2.49	3.48E-03
Slc38a1	-2.50	8.97E-04
Ccdc111	-2.50	4.11E-05
Dnajc13	-2.51	4.57E-03
Tmem167	-2.52	6.09E-03
Cul5	-2.53	3.84E-03
Cspp1	-2.56	3.55E-03
Zfp455	-2.61	4.35E-03
Mir103-2	-2.65	9.34E-03
Gm10847	-2.71	2.83E-03
Ppp2r5c	-2.73	1.40E-03
Rars2	-2.76	3.43E-04
Atp6v0d2	-2.80	1.73E-03
Fam49a	-2.89	2.58E-03
Gm6907	-2.90	2.53E-03
Ms4a4b	-2.97	5.85E-04
Itga4	-2.99	1.48E-05
Zfp518a	-3.04	3.66E-04
Mki67ip	-3.05	1.14E-03
Rnu73b	-3.10	6.85E-03
Zfp418	-3.14	2.14E-05
Gm10778	-3.25	1.02E-04
Cybb	-3.42	6.01E-06
Zfp617	-3.57	1.11E-02
4933426I21Rik	-3.58	6.18E-04
Gm4956	-3.75	3.14E-03
B230307C23Rik	-4.10	9.51E-03
Arhgef12	-4.16	1.09E-02
Art2b	-7.90	1.18E-03

10556005	Ilk	NM_010562	0.00035214
10435789	Zbtb20	ENSMUST00000(	0.00035247
10493259	Ssr2	NM_025448	0.0003533
10593130	Sidt2	NM_172257	0.00035429
10504753	LOC641050	M11024	0.00035503
10446763	Lbh	NM_029999	0.00035635
10577528	AI316807	NM_001012667	0.00035642
10415513	Parp4	NM_001145978	0.00035708
10406551	Ssbp2	NM_024272	0.00036193
10508707	Tmem200b	ENSMUST00000(	0.00036315
10377253	9130017K11Rik	ENSMUST00000(	0.00036504
10537955		---	0.00036522
10411359	Plp2	NM_019755	0.00036538
10368612	Gapdh	NM_008084	0.00036548
10596637	Mapkapk3	NM_178907	0.00036639
10548884	Erp27	NM_026983	0.00036709
10409220	Hist2h2aa1	NM_013549	0.00036758
10470788	Odf2	NM_001113213	0.00036863
10579331	Gdf15	NM_011819	0.00036951
10557806		---	0.00037061
10477233	Xkr7	NM_001011732	0.00037079
10512465	Fam166b	NM_177377	0.00037152
10453399	Srbd1	NM_030133	0.00037237
10525516	Wdr66	BC138176	0.00037817
10438358	05-Sep	NM_213614	0.00037887
10383311	Gm9734	ENSMUST00000(	0.00037953
10433977	Tmem191c	NM_177473	0.00037973
10554718	Vmn2r71	NM_001105184	0.00038109
10346298	Coq10b	NM_001039710	0.00038215
10374621	Ahsa2	NM_172391	0.0003832
10385533	Tgtp	NM_011579	0.00038332
10359097	Tor1aip1	NM_001160018	0.00038334
10410892	Rasa1	NM_145452	0.00038406
10436892	Itsn1	NM_010587	0.00038432
10455826	Megf10	NM_001001979	0.00038451
10429555	2010109I03Rik	NM_025929	0.00038588
10401149	Plek2	NM_013738	0.00038643
10390072	Gm11541	NM_001007584	0.0003876
10577337	Defb40	NM_183039	0.00038794
10340417		---	0.00038796
10440621	Grik1	NM_146072	0.00038851
10517364	A330049M08Rik	BC005730	0.0003893
10574944	Dus2l	NM_025518	0.0003905
10341117		---	0.00039126
10351277	Nme7	NM_138314	0.00039205
10483401	Spc25	NM_025565	0.00039238
10382980	Syngr2	NM_009304	0.00039291
10493789	S100a13	NM_009113	0.0003934
10536390	Glcci1	NM_133236	0.00039374
10340797		---	0.00039385
10381096	Igfbp4	NM_010517	0.00039597
10581181	Tradd	NM_001033161	0.00039669
10510580	Tnfrsf9	NM_011612	0.00039731
10597973	Lztfl1	NM_033322	0.00039981
10550870	Lypd5	NM_029806	0.00040015
10443980	Myo1f	NM_053214	0.00040029
10339745		---	0.00040205
10345406		---	0.00040235
10600604	Dmd	NM_007868	0.00040286
10429802	Plec1	NM_011117	0.00040301
10408077	Hist1h2ak	NM_178183	0.00040343
10542335	Gprc5a	NM_181444	0.00040389
10385842	Rad50	NM_009012	0.00040459
10400326	Eapp	NM_025456	0.00040544
10421325	Piwil2	NM_021308	0.00040629
10395692	Arhgap5	NM_009706	0.0004074
10348376	Heatr7b1	ENSMUST000001	0.00040793
10339920		---	0.00040852
10574163	Nlrc5	FJ889356	0.00040894
10543859	Akr1b3	NM_009658	0.0004091
10526743	Cops6	NM_012002	0.00040911
10542237		---	0.00041106
10510399	Masp2	NM_001003893	0.00041125
10453276	Thada	NM_183021	0.0004113
10574812	Ctcf	NM_181322	0.00041145
10503315	Rad54b	NM_001039556	0.00041172
10595614	2810026P18Rik	BC032970	0.00041212
10514818	BC055111	NM_183182	0.00041275
10482323	Ppp6c	NM_024209	0.00041316
10486029	Atpbd4	NM_025675	0.00041326
10464688	Ankrd13d	NM_026720	0.00041345
10358191	Camsap1l1	NM_001081360	0.0004136
10419198	Ero1l	NM_015774	0.00041394
10607346	Tspyl2	NM_029836	0.00041409
10504838	Nr4a3	NM_015743	0.00041412
10370021	Mif	NM_010798	0.00041523
10479510	9230112E08Rik	ENSMUST00000(	0.00041529
10549804	Nlrp4c	NM_031389	0.00041589
10391286	Stat5b	NM_001113563	0.00041787
10358038	Rnpep	NM_145417	0.00041848
10403943	Hist1h2bm	NM_178200	0.00041891
10598222	4930408F14Rik	BC117727	0.00041988
10597656	Scn5a	NM_021544	0.00042036
10538890	LOC641050	M11024	0.00042102
10352354	A430110L20Rik	ENSMUST00000(	0.0004226
10552715	2310016G11Rik	ENSMUST00000(	0.00042288
10543031	Slc25a13	NM_015829	0.00042389
10468909	Disp1	NM_026866	0.00042486
10517664		---	0.00042571
10501857	Gapdh	NM_008084	0.00042585
10491967	Gapdh	NM_008084	0.00042585
10496626	Gapdh	NM_008084	0.00042585
10480032	Gapdh	NM_008084	0.00042585
10545765	Gapdh	NM_008084	0.00042585
10606058	Cxcr3	NM_009910	0.0004269
10374777	Efemp1	NM_146015	0.00042726
10340094		---	0.00042963
10570321	Cul4a	NM_146207	0.00042965
10537463	Agk	NM_023538	0.00042998
10414929	Gm13949	X63928	0.00043007
10539617	Alms1	NM_145223	0.00043053
10503222	Chd7	NM_001081417	0.00043109
10535435	Zfp316	NM_017467	0.00043119
10370721	Sbno2	NM_183426	0.00043166
10371271	Zfp781	NM_199062	0.00043314
10532472	Ulk1	NM_009469	0.00043481
10491106	Pld1	NM_008875	0.00043497
10557591	Itgal	NM_008400	0.000435
10389231	Ccl3	NM_011337	0.00043504
10408600	Serpinb6a	NM_009254	0.00043529
10511298	9430015G10Rik	NM_177205	0.0004355
10606436	Nsbp1	NM_016710	0.00043659
10591763	Zfp810	NM_145612	0.00043738
10539295	Dqx1	NM_033606	0.00043772
10473919	Ckap5	NM_029437	0.00043815
10383152		---	0.00043854
10482791	Gm13531	XM_001474318	0.00043991
10351825	Tagln2	NM_178598	0.0004413

10452721	Trmt61b	NR_015549	0.00044211
10339540		---	0.00044224
10587665	4921528I07Rik	ENSMUST000000	0.00044307
10494335	Otud7b	NM_001025613	0.00044364
10441073	Kcnj6	NM_010606	0.00044398
10576603	A630001O12Rik	ENSMUST000000	0.00044501
10343386		---	0.00044503
10437080	Ttc3	NM_009441	0.00044645
10356677	Myeov2	BC147334	0.00044736
10531675	Sec31a	NM_026969	0.00044793
10403978	RP23-38E20.1	NM_001097979	0.00044857
10450145	Psmb9	NM_013585	0.00044953
10433929	Ppm1f	NM_176833	0.00045084
10556280	Swap70	NM_009302	0.00045095
10517081	Gm13213	XM_620107	0.00045216
10417940	Synpo2l	NM_175132	0.00045361
10576953		---	0.00045451
10350392	Aspm	NM_009791	0.00045568
10542050	Efcab4b	NM_001033464	0.00045629
10608701		---	0.00045728
10388409	Rpa1	NM_026653	0.00045734
10456383	Impa2	NM_053261	0.00045799
10529995	Dhx15	NM_007839	0.00046378
10533725	Gpr81	NM_175520	0.00046543
10376864	Ubb	NM_011664	0.00046574
10430179	Apol7b	NM_001024848	0.00046737
10344200		---	0.00046992
10384210		---	0.00047145
10538957	Eif2ak3	BC054809	0.00047145
10488409	9230104L09Rik	NM_029960	0.00047152
10358605	Hmcn1	NM_001024720	0.00047234
10402020	Eml5	NM_001081191	0.00047271
10399725	Sox11	NM_009234	0.00047429
10601390	Pgk1	NM_008828	0.00047482
10490159	Pmepa1	NM_022995	0.00047653
10434754	BC106179	BC106179	0.0004794
10339336		---	0.00048021
10401968	Galc	NM_008079	0.00048138
10494386	Hist1h2an	NM_178184	0.00048284
10532865	Git2	NM_019834	0.00048311
10356859	Dtymk	NM_001105667	0.00048412
10515822	Olfr1342	NM_146713	0.00048534
10494023	Rorc	NM_011281	0.00048709
10366519	5330438D12Rik	AK077360	0.00048722
10448383	Prss33	NM_001081399	0.00048737
10592023	Aplp2	NM_001102455	0.00048792
10385776	Tcf7	NM_009331	0.0004885
10429341	Ptk2	NM_007982	0.00048856
10370780	Pcsk4	NM_008793	0.0004899
10365601	Gnptab	NM_001004164	0.00049088
10593878	Snx33	NM_175483	0.00049164
10558919	Snora52	AF357388	0.00049237
10348929	Hnrnpf	NM_133834	0.0004924
10353189	Gm5523	NR_004447	0.00049509
10448214	6330416L07Rik	NM_176962	0.0004957
10373660	Olfr818	NM_146777	0.00049612
10338200		---	0.00049617
10553829	Gm7367	NR_003376	0.00049635
10553831	Gm7367	NR_003376	0.00049635
10584399	Olfr898	NM_146871	0.00049648
10492049	Fam48a	NM_019995	0.00049693
10429972	Cpsf1	NM_053193	0.00049701
10414313	Ubb	NM_011664	0.00049769
10339552		---	0.00049788
10578401	Zfp42	NM_009556	0.00049842
10352556	Gm3809	XR_032441	0.00050007
10607395	Mageh1	NM_023788	0.00050054
10503010	Fpgt	NM_029330	0.000502
10503484	Fam82b	NM_025476	0.0005036
10402598	Begain	ENSMUST000000	0.00050391
10410650	Zfp825	NM_146231	0.00050467
10346747	Cyp20a1	NM_030013	0.00050556
10379127	Spag5	NM_017407	0.00050598
10422007		---	0.0005062
10533145	Tpcn1	NM_145853	0.00050767
10437257	Zfp597	NM_001033159	0.00050786
10496592	Gbp2	NM_010260	0.00050818
10604799	Atp11c	NM_001037863	0.00050902
10454298	Zfp397	NM_027007	0.0005095
10593918	Ppcdc	NM_176831	0.00050991
10403980	RP23-38E20.1	NM_001097979	0.00051025
10576191	Spg7	NM_153176	0.00051053
10348889	D2hgdh	NM_178882	0.00051108
10349014	Capza1	NM_009797	0.00051242
10416753	Pibf1	NM_029320	0.00051384
10578690	Neil3	NM_146208	0.00051555
10556214		---	0.00051829
10447490	Pja2	NM_001025309	0.00051965
10422493	Gpr18	NM_182806	0.00051991
10514368	Gm12070	NR_002890	0.00052159
10476680	Zfp133	AF332089	0.00052284
10449419	Tead3	NM_001098226	0.00052314
10443598	Dnahc8	NM_013811	0.00052328
10604610		---	0.00052435
10461856	Gna14	NM_008137	0.00052505
10383202		---	0.00052622
10357191	Ptpn4	NM_019933	0.00052671
10349872		---	0.0005277
10495562	Lrrc39	NM_175413	0.000528
10411782	Pik3r1	NM_001077495	0.00052996
10563323	Nucb1	NM_008749	0.00053228
10512640	Gne	NM_015828	0.00053261
10502165	Sec24b	NM_207209	0.00053366
10380689	Cbx1	NM_007622	0.0005353
10354677	Ankrd44	NM_001081433	0.00053768
10416938	Gm4822	XM_139243	0.00053803
10368370	Gm8681	XR_032130	0.00053877
10510668	Tnfrsf25	NM_033042	0.00053949
10394926		---	0.00053954
10592531		---	0.00053977
10474102		---	0.00054117
10580733	Bbs2	NM_026116	0.00054295
10584578	Hspa8	M13967	0.00054321
10565156	Homer2	NM_011983	0.00054366
10535213	Snx8	NM_172277	0.0005443
10392970	Mif4gd	NM_027162	0.00054445
10585588	Cspg4	NM_139001	0.00054494
10457729	Dsc1	ENSMUST000000	0.00054508
10521136	Whsc1	NM_001081102	0.00054563
10447702	Ppih	NM_028677	0.00054586
10521498	Crmp1	NM_007765	0.00054796
10505187	Ugcg	NM_011673	0.00054904
10406426	Cetn3	NM_007684	0.00054938
10514158	Psip1	NM_133948	0.00055005
10357472	Cxcr4	NM_009911	0.00055046
10602038	E230019M04Rik	BC119078	0.00055141
10554142	2310037I24Rik	BC083325	0.00055253
10579114	Yjefn3	ENSMUST000000	0.00055316

10421143	Adam28	NM_010082	0.00055345
10584576	Hspa8	M13967	0.00055423
10566138	Olfr544	NM_020289	0.00055433
10556246	Zfp143	NM_009281	0.00055483
10400170	Prkd1	NM_008858	0.00055719
10553301	Ldha	NM_010699	0.00055764
10389865	Nme1	NM_008704	0.00055797
10578387	Zfp353	NM_153096	0.00055812
10410508	Ndufs6	NM_010888	0.00056063
10491958	Gm10729	ENSMUST00000(	0.00056102
10417183	Pcca	NM_144844	0.00056126
10537406	Clec2l	NM_001101507	0.00056129
10542275	Etv6	NM_007961	0.0005635
10344545		---	0.00056367
10608695		---	0.00056471
10338353		---	0.00056501
10588203	Ky	NM_024291	0.00056532
10504957	Smc2	NM_008017	0.00056758
10371770	Gas2l3	NM_001033331	0.00056831
10487433	Zfp661	NM_028141	0.00056888
10366746	Lrig3	NM_177152	0.00057024
10483536	4930578N16Rik	BC125570	0.00057247
10469322	Vim	NM_011701	0.00057362
10347254	Smarcal1	NM_018817	0.00057468
10419390	Gm4818	XR_033116	0.00057512
10365845	Fgd6	NM_053072	0.00057674
10569319	Ctsd	NM_009983	0.00057804
10512949	Abca1	NM_013454	0.00058048
10339391		---	0.00058127
10433462	Pmm2	NM_016881	0.00058182
10363773	Rhobtb1	NM_001081347	0.00058258
10435112	Muc4	NM_080457	0.00058361
10452980	Eif2ak2	NM_011163	0.00058399
10520782	Zfp512	NM_172993	0.00058414
10607972	Kdm5d	NM_011419	0.00058484
10394936		---	0.00058604
10505922		---	0.00058703
10608718		---	0.00058779
10462195	Kank1	NM_181404	0.00058894
10342595		---	0.0005916
10455761	Prdm6	NM_001033281	0.00059293
10504761	LOC641050	M11024	0.00059318
10400470	Cox6c	NM_053071	0.0005932
10346678	Carf	NM_139150	0.0005953
10548905	Eps8	NM_007945	0.00059695
10408937	Atxn1	NM_009124	0.0005971
10430572		---	0.00060098
10506335	Pde4b	NM_019840	0.00060393
10400304	Egln3	NM_028133	0.00060462
10488449		---	0.00060662
10578810	Clcn3	NM_173874	0.00060933
10367843	Utrn	NM_011682	0.00060985
10467068	Sgms1	NM_144792	0.00061153
10512067	Ddx58	NM_172689	0.00061181
10421293	Ppp3cc	NM_008915	0.00061237
10361091	Atf3	NM_007498	0.00061287
10475532	Sqrdl	NM_021507	0.00061423
10371220	Gna15	NM_010304	0.00061559
10435982	Btla	NM_001037719	0.00061806
10424670	Hemt1	NM_010416	0.00061813
10566366	AI451617	NM_199146	0.00061877
10399228	Gm6742	XR_030600	0.00062062
10500412	Gpr89	NM_026229	0.00062072
10412701	3830406C13Rik	NM_146051	0.00062282
10350819	Csnk2a1	NM_007788	0.00062291
10532921	Sppl3	NM_029012	0.00062508
10595638	4930579C12Rik	ENSMUST00000(	0.00062575
10376245	Gria1	NM_001113325	0.00062582
10500677	Cd2	NM_013486	0.00062584
10454782	Egr1	NM_007913	0.00062628
10340331		---	0.00062709
10572253	Sf4	NM_027481	0.00062813
10420637	Kpna3	NM_008466	0.00062938
10527573		---	0.00063214
10457223	Cdh11	NM_009866	0.00063401
10608680		---	0.00063417
10518364	Gm13143	XM_204069	0.00063581
10345807	Il18r1	NM_008365	0.00063595
10493585	Ube2q1	NM_027315	0.00063738
10496182	Cxxc4	NM_001004367	0.00063802
10400210	Hectd1	NM_144788	0.00063928
10398635	Amn	NM_033603	0.00063974
10406598	Serinc5	NM_172588	0.00064
10414431	Gapdh	NM_008084	0.00064348
10341517		---	0.00064359
10433163	Ppp1r1a	NM_021391	0.0006465
10401278	Erh	NM_007951	0.00064924
10407481	Pfkp	NM_019703	0.00065083
10338056		---	0.00065132
10338037		---	0.00065169
10537296		---	0.00065189
10593526	Atm	NM_007499	0.00065193
10498076	Maml3	NM_001004176	0.00065276
10570356	Tmco3	NM_172282	0.00065299
10578904	Cpe	NM_013494	0.00065416
10400706	Arf6	ENSMUST00000(	0.00065614
10480901	Nacc2	NM_001037098	0.00065814
10519607	4930420K17Rik	BC147127	0.00065911
10496379	H2afz	NM_016750	0.00066005
10422075	Mycbp2	NM_207215	0.0006602
10407300	4930544M13Rik	ENSMUST00000(	0.00066033
10482301	Scai	NM_178778	0.00066161
10475144	Capn3	NM_007601	0.00066173
10374821	Smek2	NM_134034	0.00066539
10424781	Grina	NM_023168	0.00066627
10413977	Gm626	XM_985917	0.00066743
10559389	Mrgprd	NM_203490	0.0006677
10592493	4931429I11Rik	NM_001081121	0.00066783
10430871	Tdg	NM_011561	0.00066898
10495873	Ptma	NM_008972	0.00067196
10366705	Gm9081	XR_030920	0.00067465
10473022	Plp2	NM_019755	0.00067467
10540472	Bhlhe40	NM_011498	0.00067764
10466837		---	0.00067959
10492558	Smc4	NM_133786	0.00068027
10507475		---	0.00068033
10560202	Gltscr1	NM_001081418	0.00068046
10471677	Dab2ip	NM_001114124	0.00068077
10422026	1700110M21Rik	NM_029680	0.00068184
10412394	Nnt	NR_003544	0.00068325
10451993	D17Wsu104e	NM_080837	0.00068343
10451004	Cd2ap	NM_009847	0.00068344
10419323	Dlgap5	NM_144553	0.00068745
10456754	Gm10532	ENSMUST00000(	0.00068778
10458195	Cdc25c	NM_009860	0.00069024
10505451	Orm2	NM_011016	0.00069113
10475487	Slc28a2	NM_172980	0.00069514
10436203	Gm4416	XM_001478876	0.00069517

10515884	Ppih	NM_028677	0.0006956
10448186	Zfp820	NM_029281	0.00069586
10488584	4930556L07Rik	AK016149	0.00069611
10362454	Trdn	NM_029726	0.00069677
10408111	Hist1h2ao	NM_178185	0.00069767
10582719	Sipa1l2	NM_001081337	0.00069772
10573261	Asf1b	NM_024184	0.00069775
10376060	Irf1	NM_008390	0.0006989
10605315	Lage3	NM_025410	0.00069984
10500896	H3f3b	NM_008211	0.00069987
10403834	Sfrp4	NM_016687	0.00070169
10523231	Art3	NM_181728	0.0007017
10389143	Slfn8	NM_181545	0.00070217
10484894	Ptprj	NM_008982	0.00070438
10459844	Haus1	NM_146089	0.0007051
10598912	Pctk1	NM_011049	0.00070512
10472235	Dapl1	NM_029723	0.0007052
10348180	Eif4e2	NM_001039169	0.00070666
10398459	Ppp2r5c	NM_001135001	0.00070725
10408072	Hist1h2ao	NM_178185	0.00070854
10455299	Sh3rf2	NM_001146299	0.00070865
10403955	Hist1h2ao	NM_178185	0.00070869
10439514	Gap43	NM_008083	0.00070909
10408070	RP23-38E20.1	NM_001097979	0.00071111
10568328	Vkorc1	NM_178600	0.00071284
10351873	Pyhin1	NM_175026	0.00071291
10437174	Wrb	NM_207301	0.00071303
10587892	Atr	NM_019864	0.00071391
10486858	Mfap1a	NM_026220	0.00071632
10508151	Clspn	NM_175554	0.00071719
10558450	Gm10578	ENSMUST00000(	0.00071732
10479950	Cugbp2	NM_001110231	0.00071815
10566233	Olfr623	NM_147122	0.00071835
10456254	Nedd4l	NM_001114386	0.00072286
10398267	Evl	NM_007965	0.00072403
10460833	Ehd1	NM_010119	0.00072405
10341112		---	0.00072438
10559261	Cd81	NM_133655	0.0007246
10515590	Kdm4a	NM_001161823	0.00072694
10408450	Sox4	NM_009238	0.00072727
10449303	Bak1	NM_007523	0.00072729
10547015	1500001M20Rik	NM_026894	0.00072884
10511139	Ssu72	NM_026899	0.00073004
10524790	Cit	NM_007708	0.00073032
10450101		---	0.00073175
10472042	Gm13498	AF390105	0.00073176
10497931	Cflar	NM_009805	0.00073188
10414417	Peli2	NM_033602	0.00073255
10387180	Ndel1	NM_023668	0.00073277
10414271	Ptger2	NM_008964	0.00073358
10504470	Melk	NM_010790	0.00073376
10508936	Ubxn11	NM_026257	0.00073499
10483246		---	0.00073576
10348277	Atg16l1	NM_029846	0.00073787
10405234	Snrpd2	NM_026943	0.00073858
10474541	Nop10	NM_025403	0.00074098
10595189	Slc17a5	NM_172773	0.00074118
10560117	Gm6955	ENSMUST00000(	0.00074647
10408666	Rpp40	NM_145938	0.00074717
10498595	Snrpd2	NM_026943	0.00075233
10358928	Cacna1e	NM_009782	0.00075332
10505643	4930473A06Rik	BC039810	0.00075376
10384522	Actr2	NM_146243	0.00075464
10583320	BC017612	NM_133214	0.00075574
10362456	Trdn	NM_029726	0.00075578
10429444	Gm628	ENSMUST000001	0.00075746
10339457		---	0.00075937
10375402	Adam19	NM_009616	0.00076055
10397450	Vash1	NM_177354	0.00076123
10473491	Olfr1033	NM_146578	0.00076493
10408156	V1rh14	NM_134239	0.00076589
10439312	Cd86	NM_019388	0.00076618
10495668	4930432M17Rik	ENSMUST00000(	0.00076638
10372716	Rap1b	NM_024457	0.0007667
10425903	Gapdh	BC092294	0.0007682
10511290	Tnfrsf18	NM_009400	0.0007684
10428648	Taf2	NM_001081288	0.00076919
10465895	Fads2	NM_019699	0.00076962
10433088	Cbx5	NM_007626	0.00077092
10408210	Hist1h2bc	NM_023422	0.00077107
10480064	Fbxo18	NM_015792	0.00077137
10343809		---	0.00077195
10480601	A830007P12Rik	BC013092	0.00077256
10557342	Il21r	NM_021887	0.0007728
10390640	Ikzf3	NM_011771	0.00077342
10580486	Brd7	NM_012047	0.00077369
10361869	Nhsl1	NM_173390	0.00077402
10537078	Mkln1	NM_013791	0.00077687
10446253	Vav1	NM_011691	0.00077692
10392953	Gga3	NM_173048	0.00077725
10412650	Kctd6	NM_027782	0.00077966
10485989	Aqr	NM_009702	0.00078106
10587486	Set	NM_023871	0.00078132
10424804	Exosc4	NM_175399	0.0007823
10539813	Ccdc48	NM_001159697	0.00078251
10383198		---	0.00078267
10391963	Nsf	NM_008740	0.00078348
10344170		---	0.00078621
10542636	Pyroxd1	NM_183165	0.00078644
10566574	Gvin1	NM_029000	0.00078764
10408225	Hist1h4c	NM_178208	0.00078868
10343204		---	0.00078946
10587942	Xrn1	NM_011916	0.00079
10590623	Cxcr6	NM_030712	0.00079238
10554817	Gapdh	NM_008084	0.00079399
10409767	Golm1	NM_027307	0.00079578
10510061	Pramef8	BC056931	0.00079707
10532150	Fam69a	NM_026062	0.00079928
10607002	Gm7091	XR_031774	0.00080007
10504375	Npr2	NM_173788	0.00080055
10362310	Ube2l3	NM_009456	0.00080095
10524150	Ankle2	NM_027922	0.00080111
10565241	1700026D08Rik	BC061016	0.0008032
10366703	Gm4801	XM_137324	0.00080381
10532624	Myo18b	NM_028901	0.00080481
10387372	Kdm6b	NM_001017426	0.00080561
10444911	Mdc1	NM_001010833	0.00080642
10568050	Aldoa	NM_007438	0.00080819
10562059	Cox6b1	NM_025628	0.0008082
10364511	9130017N09Rik	BC013508	0.00080845
10360920	Tgfb2	NM_009367	0.00080865
10364950	Gadd45b	NM_008655	0.00080887
10564573	Chd2	NM_001081345	0.00081083
10395702	Akap6	NM_198111	0.00081133
10338287		---	0.0008118
10554549	Whamm	NM_001004185	0.00081603
10563037	Scaf1	NM_001008422	0.0008165
10388591	Cpd	NM_007754	0.0008165

10592705	Pou2f3	NM_011139	0.00081788
10536220	Col1a2	NM_007743	0.00081978
10444780	H2-D1	NM_010380	0.00081992
10482432	Gapdh	NM_008084	0.00082018
10375019	Nsg2	NM_008741	0.00082124
10415645	Mrp63	NM_026401	0.00082174
10345074	Cetn4	NM_145825	0.00082215
10357008	Pign	NM_013784	0.00082399
10344398		---	0.00082482
10432411	Mcrs1	NM_016766	0.00082596
10350159	Lad1	NM_133664	0.00082634
10561806	Mif	NM_010798	0.00082662
10412251	Ndufs4	NM_010887	0.00082841
10599348	Gria3	NM_016886	0.00082882
10499716	Ubap2l	NM_028475	0.0008304
10500928	Capza1	NM_009797	0.00083081
10506154	Alg6	NM_001081264	0.00083116
10555885		---	0.00083433
10561302	Pgam1	NM_023418	0.00083449
10382502	Cdr2l	NM_001080929	0.00083526
10559766	Zfp583	NM_001033249	0.00083835
10549282	Itpr2	NM_019923	0.0008397
10547943	Ncapd2	NM_146171	0.00084027
10369264	Oit3	NM_010959	0.00084114
10562685		---	0.00084134
10604597	Rps17	NM_009092	0.00084159
10404026	Hist1h2af	NM_175661	0.00084207
10573172	Clgn	NM_009904	0.00084606
10528702	Prkag2	NM_145401	0.00084606
10592816	Hmbs	NM_013551	0.00084667
10511382	Nsmaf	NM_010945	0.00084774
10352554		---	0.00084793
10443690	Glp1r	NM_021332	0.00084811
10338460		---	0.00085059
10566241	Olfr630	NM_147098	0.00085195
10606355	Cysltr1	NM_021476	0.00085251
10365428	Btbd11	NM_028709	0.00085315
10468517	Mxi1	NM_010847	0.00085336
10488145		---	0.00085344
10550521	Mypop	NM_145579	0.00085559
10498345	Gpr171	NM_173398	0.00085782
10419611	Supt16h	NM_033618	0.00086206
10378697	Myo1c	NM_008659	0.00086208
10373740	Pik3ip1	NM_178149	0.00086466
10343617		---	0.00086477
10384102	Npc1l1	NM_207242	0.00086524
10462140	Dock8	NM_028785	0.00086635
10496015	Pla2g12a	NM_183423	0.00086671
10373651	Olfr814	ENSMUST00000(	0.00086781
10350343	Atp6v1g3	NM_177397	0.00086962
10514054	Nfib	NM_001113209	0.00086973
10518145	Prdm2	ENSMUST00000(	0.00087039
10412064		---	0.00087228
10580219	Calr	NM_007591	0.0008723
10413987	Gm626	XM_985917	0.0008739
10586604	Rps27l	NM_026467	0.00087566
10486203	Ino80	NM_026574	0.00087851
10370573	Theg	NM_011583	0.00087946
10544768	Hoxa5	NM_010453	0.00087951
10355329	Bard1	NM_007525	0.0008798
10363161	6330442E10Rik	BC079613	0.00087998
10568464	Ate1	NM_013799	0.0008807
10485667	Dnajc24	NM_026992	0.00088092
10351658	Cd48	NM_007649	0.00088333
10607206	Il13ra2	NM_008356	0.00088555
10565775	Dgat2	NM_026384	0.00088623
10414984	Gm13892	ENSMUST000001	0.00088778
10582599	Nup133	NM_172288	0.00088883
10541307	Usp18	NM_011909	0.00088919
10429957	Fbxl6	NM_013909	0.00088952
10367579	LOC100045113	XM_001473697	0.00088988
10420860	Elp3	NM_028811	0.00089128
10349376		---	0.00089211
10591369	Dnmt1	NM_010066	0.00089236
10431872	Slc38a1	ENSMUST000001	0.00089316
10366848	B4galnt1	NM_008080	0.00089327
10599317	Rhox13	ENSMUST00000(	0.00089399
10467650	Frat2	NM_177603	0.00089742
10575976	Crispld2	NM_030209	0.00089795
10602590		---	0.00090147
10384183	Nacad	NM_001081652	0.00090277
10586130	Aagab	BC027311	0.00090461
10496295	Ube2d3	NM_025356	0.00090564
10489660	Elmo2	NM_207706	0.00090571
10569485	Tnfrsf26	NM_175649	0.0009064
10481678	6330409D20Rik	ENSMUST00000(	0.00090665
10598207			0.00090854
10491136	Tnik	NM_026910	0.00091011
10591781	Anln	NM_028390	0.00091214
10484371	Calcrl	NM_018782	0.00091577
10344807	Cspp1	NM_026493	0.00091629
10373684	Vmn2r86	NM_001103365	0.00091631
10343552		---	0.00091815
10400504	Foxa1	NM_008259	0.00091848
10488322	A230067G21Rik	NM_001033348	0.00092062
10562132	Cd22	NM_001043317	0.00092119
10499655	Il6ra	NM_010559	0.00092177
10409508	Ddx41	NM_134059	0.0009225
10492231	Med12l	NM_177855	0.00092274
10361807	Hivep2	NM_010437	0.00092416
10355152	Fzd5	NM_022721	0.00092499
10552210	Ankrd27	NM_145633	0.00092569
10501143	Ahcyl1	NM_145542	0.00092594
10557213	Rbbp6	NM_011247	0.00092661
10608721		---	0.00092755
10492172		---	0.0009279
10429140	Ndrg1	NM_008681	0.00092811
10408085	Hist1h2an	NM_178184	0.00092936
10561799	Zfp74	NM_178384	0.00093231
10481835	Lmx1b	NM_010725	0.00093387
10360018	Fcrla	NM_001160215	0.00093399
10562847	Nr1h2	NM_009473	0.00093497
10603151	Gpm6b	NM_023122	0.00093537
10470186	Lcn14	NM_001099301	0.00093539
10382912	09-Sep	NM_001113486	0.00093556
10398173	Vrk1	NM_011705	0.00093559
10409799	Isca1	NM_026921	0.00093813
10432101	Senp1	NM_144851	0.00093841
10457359	Mpp7	NM_001081287	0.00093841
10504817	Tgfbr1	NM_009370	0.00093842
10597518	Tgfbr2	NM_009371	0.00093883
10566097	Nup98	NM_022979	0.00093899
10383365	Hgs	NM_001159328	0.00094072
10428728	9330154K18Rik	ENSMUST00000(	0.00094267
10530787	Aasdh	NM_173765	0.00094329
10559297	Slc22a18	NM_001042760	0.00094492
10515431	Kif2c	NM_134471	0.00094578
10538588	Gm5569	NM_001101533	0.00094622

10447395	Msh2	NM_008628	0.00094631
10423185	Rnasen	NM_001130149	0.00094667
10569569	Cttn	NM_007803	0.0009503
10463121	Zfp518	NM_028319	0.00095036
10480329	Dnajc1	NM_007869	0.00095048
10468691	Ablim1	NM_178688	0.00095515
10427454	Card6	ENSMUST00000	0.00095706
10472501	Lass6	NM_172856	0.00095777
10521626	Cc2d2a	NM_172274	0.00095852
10389526	Cltc	NM_001003908	0.00095879
10521090	Tacc3	NM_001040435	0.00095915
10580033	Cd97	NM_011925	0.00096
10561247	Shkbp1	NM_138676	0.00096064
10361031	Rps6kc1	NM_178775	0.00096091
10398147	Papola	NM_011112	0.00096352
10572949	Nr3c2	NM_001083906	0.00096428
10458138	Brd8	NM_030147	0.00096517
10378802	Blmh	NM_178645	0.00096581
10428998	Asap1	NM_010026	0.00096616
10380732	Mrpl10	NM_026154	0.00096644
10441032	Cldn14	NM_019500	0.00096698
10503551	Usp45	NM_152825	0.00096707
10592725	Gm10688	ENSMUST00000	0.00096741
10372457	Tbc1d15	NM_025706	0.00097062
10522658	C530008M17Rik	ENSMUST00000	0.0009712
10395672	Ap4s1	NM_021710	0.00097154
10545958	Anxa4	NM_013471	0.00097256
10584941	Bace1	NM_011792	0.00097286
10524284	Ttc28	BC046779	0.00097288
10545154	Il23r	NM_144548	0.00097377
10494972	Bcl2l15	NM_001142959	0.00097486
10474064	Trp53i11	NM_001025246	0.00097638
10551252	Nlrp9a	NM_001048219	0.0009765
10428857	Mtss1	NM_001146180	0.00097701
10440543	Rnf160	NM_001081068	0.00097826
10340932		---	0.00097978
10359201	Ralgps2	NM_001159965	0.00098046
10531193	Adamts3	NM_001081401	0.00098194
10563709	Mrgpra1	NM_153095	0.00098202
10342392		---	0.00098287
10340326		---	0.000984
10559498	9430041J12Rik	AK051461	0.00098446
10564813	Mesp1	NM_008588	0.00098503
10343572		---	0.00098582
10405994	Med10	NM_138596	0.00098725
10360373	E030037K03Rik	ENSMUST00000	0.00098762
10390869	Krt39	NM_213730	0.00099154
10386947	Gapdh	NM_008084	0.0009945
10371321	Slc41a2	NM_177388	0.00099536
10535053	Prkar1b	NM_008923	0.00099556
10343937		---	0.00099795
10603180	Chchd5	BC027303	0.00099823
10569278	5530400B01Rik	ENSMUST00000	0.00100176
10454212	Mep1b	NM_008586	0.00100502
10554693	Stard5	NM_023377	0.00100553
10504132	Ccl19	NM_011888	0.00100646
10410259	Uqcrb	NM_026219	0.00100817
10404063	Hist1h2ab	NM_175660	0.00100914
10401238	Zfp36l1	NM_007564	0.00101155
10476252	Cdc25b	NM_023117	0.00101265
10390635	1810046J19Rik	NM_025559	0.00101295
10590628	Ccr3	NM_009914	0.00101564
10348301	Sag	NM_009118	0.00101639
10464504	Lrp5	NM_008513	0.00101972
10343477		---	0.00102044
10385236	Akr1b3	NM_009658	0.00102142
10453114	Dhx57	NM_198942	0.00102254
10571696	Casp3	NM_009810	0.00102335
10443561	1300018I05Rik	BC024691	0.0010238
10500685	Atp1a1	NM_144900	0.00102586
10500157	Setdb1	NM_018877	0.00102665
10414754	Tcra	BC147846	0.001027
10414860	Tcra	BC147846	0.001027
10338145		---	0.0010297
10447128	Gm6594	XM_890094	0.00103161
10354768	Akr1b3	NM_009658	0.00103332
10592217	Stt3a	NM_008408	0.00103437
10410264	Mterfd1	NM_025547	0.00103472
10598238		---	0.0010348
10447383	Epcam	NM_008532	0.00103532
10501971	Ank2	NM_178655	0.00103766
10484612	Olfr1079	NM_146407	0.00104207
10442141	V1re5	NM_134194	0.00104297
10535883	Katnal1	NM_153572	0.00104622
10589938	Gpd1l	NM_175380	0.00104696
10601659	Srpx2	NM_026838	0.00104774
10424493	Tg	NM_009375	0.00104799
10344446		---	0.00104826
10513156	Ptpn3	NM_011207	0.00105025
10420198	Ripk3	NM_019955	0.00105254
10544660	Osbpl3	NM_027881	0.00105263
10381072	Cdc6	NM_011799	0.00105365
10466314	Gm6545	XM_889589	0.00105508
10582008	2310061C15Rik	NM_026844	0.0010551
10587262	Klhl31	NM_172925	0.00105755
10341477		---	0.00105864
10385572	Sqstm1	NM_011018	0.00105878
10530692	Kdr	NM_010612	0.00105887
10597427	Trim71	NM_001042503	0.00105981
10363894	Ipmk	NM_027184	0.00106
10438071	Ppil2	NM_144954	0.00106038
10549875	Olfr1350	NM_146389	0.00106071
10397428	1700020O03Rik	BC048169	0.00106107
10520483	D5Wsu178e	NM_027652	0.00106163
10474335	Immp1l	NM_028260	0.00106602
10449839	Akap8	NM_019774	0.00106865
10415293	Rnf31	NM_194346	0.0010688
10441973	Tbp	NM_013684	0.00107127
10605113	L1cam	NM_008478	0.00107601
10392328	Nol11	NM_133702	0.00107828
10393408	Tmc6	NM_145439	0.00107948
10498313	Pgk1	NM_008828	0.00107971
10399379	Pgk1	NM_008828	0.00107971
10555460	Stard10	NM_019990	0.001081
10523206	Uso1	NM_019490	0.00108133
10428908		---	0.00108155
10371811	Scyl2	NM_198021	0.00108175
10434523	Polr2h	NM_145632	0.00108228
10384223	Igfbp3	NM_008343	0.00108231
10446441	Ddx11	NM_001003919	0.00108439
10459944	Nfatc1	NM_198429	0.00108534
10530536	Tec	NM_001113460	0.0010857
10543522	Gm5303	XR_033050	0.00108594
10428936		---	0.00108847
10533003	1110006O24Rik	NR_027810	0.00109198
10346365	Sgol2	NM_199007	0.00109245
10455970	BC023105	BC023105	0.00109492
10524098	Zfp605	BC007165	0.00109514

10526113	Crcp	NM_007761	0.00109609
10370376	Pfkl	NM_008826	0.00109693
10489985	Atp9a	NM_015731	0.00110127
10443940	Zfp422-rs1	NM_029952	0.00110184
10522716	Polr2b	NM_153798	0.001104
10568461	Rfwd3	NM_146218	0.00110511
10525983	Ran	NM_009391	0.0011052
10423842	Dcaf13	NM_198606	0.00110578
10524515	Myo1h	BC144867	0.00110667
10367076	Prim1	NM_008921	0.00110754
10489440	Wfdc15a	NM_183271	0.00110802
10340098		---	0.00110937
10463930	Mxi1	NM_010847	0.00110965
10474545	Slc12a6	NM_133649	0.00111083
10569017	Ifitm3	NM_025378	0.00111112
10425522	Rbx1	NM_019712	0.00111756
10353878	Ankrd23	NM_153502	0.00111842
10514221	Plin2	NM_007408	0.00111848
10503182	Chd7	NM_001081417	0.00111879
10494662	Ywhah	NM_011738	0.00112338
10514340	Cdkn2a	NM_001040654	0.00112416
10449741	Sik1	NM_010831	0.00112616
10567739	4933440M02Rik	ENSMUST00000(	0.00112639
10476759	Rin2	NM_028724	0.00112648
10341395		---	0.00112829
10348829	Farp2	NM_145519	0.0011292
10557201	Cacng3	NM_019430	0.00113256
10506500	Usp24	ENSMUST00000(	0.00113359
10434281	LOC635992	ENSMUST00000(	0.00113723
10538135	Gimap7	NM_146167	0.00113726
10436600		---	0.00113738
10410295	Zfp595	NM_177622	0.00113758
10552341	Vmn2r60	NM_001105057	0.0011376
10544114	Hipk2	NM_010433	0.00113888
10472050	Tnfaip6	NM_009398	0.00114114
10425834	Samm50	NM_178614	0.00114349
10373372	Coq10a	NM_001081040	0.00114514
10593024	Cd3e	NM_007648	0.00114572
10546152	Podxl2	NM_176973	0.00114683
10419977	Ap1g2	NM_007455	0.00115141
10351679	Cd84	NM_013489	0.0011516
10556067	Syt9	NM_021889	0.00115187
10434029	Lztr1	NM_025808	0.00115327
10360173	Slamf7	NM_144539	0.00115329
10476728	Dtd1	NM_025314	0.00115572
10457872	Slc39a6	NM_139143	0.00115625
10542477	Pik3c2g	NM_207683	0.0011568
10608664		---	0.00115682
10552566	Klk1b9	NM_010116	0.0011577
10524312	Ttc28	ENSMUST000001	0.00115781
10566229	Olfr624	NM_001011865	0.00115817
10353679	Gm5697	XR_004787	0.00115825
10503123	Ubxn2b	NM_026534	0.00115945
10379953	4632419I22Rik	BC067002	0.00115993
10434384	Ap2m1	NM_009679	0.00116006
10579769	Gapdh	NM_008084	0.00116068
10404132	Cmah	NM_001111110	0.00116091
10457546	Osbpl1a	NM_207530	0.00116098
10427369	Pde1b	NM_008800	0.00116166
10346235	Hibch	NM_146108	0.00116286
10359762	Rcsd1	NM_178593	0.00116541
10359828	Gm4847	ENSMUST00000(	0.00116778
10539486	Zfml	NM_008717	0.00117087
10414703		---	0.00117193
10482509	Rbm43	NM_001141981	0.00117242
10344624	Lypla1	NM_008866	0.00117245
10598996	Gm5124	NM_198633	0.00117246
10427402	Ghr	NM_010284	0.00117284
10341146		---	0.00117308
10577910	Fnta	NM_008033	0.00117445
10575775	4933407C03Rik	BC158118	0.00117491
10502638	Clca5	NM_178697	0.00117498
10471929	Arhgap15	NM_153820	0.00117526
10352503	Bpnt1	NM_011794	0.00117658
10367154	Gls2	NM_001033264	0.00117766
10472128	Arl6ip6	NM_022989	0.00117859
10343835		---	0.00117874
10532103	Glmn	NM_133248	0.00117904
10383351	Gm9981	ENSMUST00000(	0.00118194
10529953	Gm10048	AK036806	0.00118197
10566690	Olfr493	NM_146310	0.00118732
10571325	Mfhas1	NM_001081279	0.00118798
10600169	Bgn	NM_007542	0.00118888
10344817	Cspp1	NM_026493	0.00118902
10351293	Dpt	NM_019759	0.00119635
10558001	Inpp5f	NM_178641	0.00119685
10484249	Cwc22	NM_030560	0.00119853
10373407	Esyt1	NM_011843	0.00119931
10376021	08-Sep	NM_033144	0.00120111
10341450		---	0.00120171
10471655	Gsn	NM_146120	0.00120234
10369479	Lrrc20	ENSMUST00000(	0.00120479
10373054	Slc26a10	BC089587	0.00120542
10527538	Rasl11a	NM_026864	0.0012057
10497487		---	0.00120815
10481349	Ntng2	NM_133501	0.00121007
10499552	Efna4	NM_007910	0.00121196
10358717	1700025G04Rik	NM_197990	0.00121203
10471608	Cep110	NM_012018	0.00121278
10600082	Nsdhl	NM_010941	0.00121393
10338726		---	0.00121526
10548563	Ptp4a1	NM_011200	0.00121831
10412981	Sec24c	NM_172596	0.00121881
10403229	Itgb8	NM_177290	0.00121931
10419638	Chd8	NM_201637	0.00122263
10594613	Usp3	NM_144937	0.00122319
10530142	Klf3	NM_008453	0.00122358
10392207	Tex2	NM_198292	0.00122481
10547830	Tpi1	NM_009415	0.00122595
10411107	Cmya5	ENSMUST00000(	0.00123031
10399965	F730043M19Rik	ENSMUST00000(	0.00123261
10490946	Hsp90aa1	NM_010480	0.00123344
10338915		---	0.00123568
10395201	Slc26a3	NM_021353	0.00123598
10441167	Lca5l	NM_001001492	0.00123632
10395976	Dnajb6	NM_011847	0.00123663
10552681	Josd2	NM_025368	0.0012437
10549813	Zfp444	NM_001146024	0.0012466
10343655		---	0.00124831
10593842	Tspan3	NM_019793	0.00124951
10417144	Timm8a2	NM_001037744	0.00124989
10382010	Dcaf7	NM_027946	0.00125212
10550723	Nkpd1	BC118925	0.00125246
10605792	Spin4	NM_178753	0.00125361
10605790	Gm371	XM_905158	0.0012547
10548455	Klra1	NM_016659	0.00125525
10360026	Uqcrb	NM_026219	0.00125605
10414156	4930474N05Rik	BC096580	0.00125728

10518837	Camta1	NM_001081557	0.0012573
10527024	Gm10091	ENSMUST00000(	0.00125831
10568605	Ctbp2	NM_009980	0.00126044
10440258	Epha3	NM_010140	0.00126113
10384123	Ddx56	NM_026538	0.00126131
10546294	Nup210	NM_018815	0.00126167
10531794	Wdfy3	AB093277	0.00126267
10394560	Smc6	NM_025695	0.00126307
10523923	Ccdc18	NM_028481	0.00126317
10543360	Gm5301	XM_621704	0.00126423
10443550	Rnf8	NM_021419	0.0012643
10376986	AU040829	NM_001099288	0.00126555
10517165	Cd52	NM_013706	0.00126556
10382542	Nup85	NM_001002929	0.00127292
10391461	Brca1	NM_009764	0.00127335
10563722	Mrgpra3	NM_153067	0.001274
10461093	Pla2g16	NM_139269	0.00127412
10497689	Gnb4	NM_013531	0.00127433
10358299	EG214403	NM_001029977	0.00127584
10538148		---	0.00127758
10526693	Zcwpw1	NM_001005426	0.00128132
10462670	Rpp30	NM_019428	0.00128255
10603306		---	0.00128326
10569719	A430078G23Rik	NM_001033378	0.00128543
10595169		---	0.00128719
10452867	Dpy30	NM_001146222	0.00129016
10445067	1700022C21Rik	BC027007	0.00129018
10463189	Ankrd2	NM_020033	0.00129274
10436873	Son	NM_178880	0.00129299
10507833	Nt5c1a	NM_001085502	0.00129318
10444312	Btnl2	NM_079835	0.00129813
10549447	Ipo8	NM_001081113	0.00129819
10361979	Bclaf1	NM_001025392	0.00129961
10566050	Il18bp	NM_010531	0.00130265
10473281	Itgav	NM_008402	0.00130499
10463997	Pdcd4	NM_011050	0.00131121
10412559	Slbp	NM_009193	0.00131361
10509965	Epha2	NM_010139	0.00131445
10380381	Tob1	NM_009427	0.00131493
10449977	4921501E09Rik	NM_001009544	0.00131544
10546510	Lrig1	NM_008377	0.00131754
10349081	Phlpp1	BC059254	0.00131927
10511510	Ints8	NM_001159595	0.00131934
10498485	Slc33a1	NM_015728	0.00131996
10342513		---	0.00132165
10338256		---	0.00132268
10597461	Cmtm7	NM_133978	0.00132503
10477644	Trp53inp2	NM_178111	0.00132578
10344405		---	0.00132683
10525916	Tmem132b	ENSMUST00000(	0.00132686
10531994	Mpa2l	NM_194336	0.0013285
10454441	Syt4	NM_009308	0.00132959
10339990		---	0.00133091
10606552	Gm14920	NM_001102665	0.00133328
10601553	Gm14920	NM_001102665	0.00133328
10402360	Serpina10	NM_144834	0.00133418
10529741	Rab28	NM_027295	0.00134304
10403899	Olfr42	NM_010984	0.00134614
10349453	Rab3gap1	NM_178690	0.00134636
10416037	Pbk	NM_023209	0.00134725
10340168		---	0.00134999
10339590		---	0.00135146
10343184		---	0.00135252
10576944	Gm10067	ENSMUST00000(	0.00135398
10484777	Olfr1216	NM_146893	0.00135409
10542875	3010003L21Rik	BC106181	0.00135606
10581378	Psmb10	NM_013640	0.00135616
10345200		---	0.00135733
10457745	D030074E01Rik	NM_029491	0.00135828
10520698	Dnajc5g	NM_177677	0.0013585
10528480	Gm6560	XR_033533	0.00135891
10566452	Gm4972	XR_035686	0.00135904
10500329	Hist2h2aa1	NM_013549	0.00135933
10486616	Ubr1	NM_009461	0.00135979
10551614	Ech1	NM_016772	0.00136055
10440471	Mrpl39	NM_017404	0.00136389
10584582	Gm10694	ENSMUST00000(	0.00136503
10437330	Crebbp	ENSMUST00000(	0.00136692
10425321	Apobec3	NM_001160415	0.00136879
10466848	D19Bwg1357e	NM_177474	0.00136939
10368534	Ncoa7	NM_172495	0.00136971
10562905	Atf5	NM_030693	0.00137554
10460018	Zfp236	BC138187	0.00137715
10377681	Dullard	NM_026017	0.00137716
10409190	Cenpp	NM_025495	0.001378
10357155	Inhbb	NM_008381	0.00138028
10554839	Picalm	NM_146194	0.00138293
10537882	Cul1	NM_012042	0.00138408
10604879	Gm6783	XR_031567	0.0013842
10405662	Mak10	NM_030153	0.00138544
10424250	Snrpd2	NM_026943	0.00138684
10593471		---	0.00138839
10521085	Gm9903	ENSMUST00000(	0.00139099
10338054		---	0.00139177
10404996	Ninj1	NM_013610	0.00139297
10560862	2310033E01Rik	BC139039	0.00139781
10401244	Actn1	NM_134156	0.00139954
10598062		---	0.00139983
10416419	Gm10847	ENSMUST00000(	0.00140315
10400967	Six1	NM_009189	0.00140347
10463068	Ptp4a1	NM_011200	0.00140347
10565349	Vmn2r66	NM_001033878	0.00140434
10447564	Zdhhc14	NM_146073	0.00140441
10389339	Usp32	NM_001029934	0.00140925
10387743	Slc2a4	NM_009204	0.00141069
10490731	Sox18	NM_009236	0.0014165
10365037	Matk	NM_010768	0.00141791
10377547	Kdm6b	NM_001017426	0.00141964
10341041		---	0.00142035
10453216	Thumpd2	BC065413	0.00142094
10360631	Cabc1	NM_023341	0.00142332
10569341	H19	NR_001592	0.00142562
10544499	A930035D04Rik	AK080300	0.00142732
10423941	Ttc35	NM_025736	0.00142824
10452202	Acer1	NM_175731	0.00142903
10346943	Creb1	NM_133828	0.00143083
10599997	Mtmr1	NM_016985	0.00143116
10475437	Sord	NM_146126	0.0014334
10601888	Plp1	NM_011123	0.00143441
10486867	Mfap1a	NM_026220	0.00143684
10367073	Snrpd2	NM_026943	0.0014382
10545458	Tcf3	NM_001079822	0.00143856
10453717	Fabp5l2	ENSMUST00000(	0.00143978
10338340		---	0.00144385
10484710	Olfr73	NM_054090	0.00144563
10550003	Zfp606	NM_026112	0.0014466
10511779	Atp6v0d2	NM_175406	0.0014474
10519659	Gm3360	XR_032742	0.00144793

10542257	Tas2r125	NM_207027	0.00144868
10445774	B430306N03Rik	AY522648	0.00145023
10460359	Coro1b	NM_011778	0.00145059
10555681	Stim1	NM_009287	0.00145109
10458992	C330018D20Rik	ENSMUST000000	0.00145402
10510991	Morn1	NM_001081100	0.00145646
10588538	Iqcf1	NM_028843	0.00145819
10455249	9630014M24Rik	ENSMUST000000	0.00145822
10459481	Lman1	NM_027400	0.00146231
10428018	Ube2v2	NM_023585	0.00146281
10574098	Nlrc5	FJ889356	0.00146507
10491197	Gm1527	NM_001033479	0.00146627
10340613		---	0.00146693
10462442	Il33	NM_133775	0.0014674
10572870	Hmgxb4	NM_178017	0.00146787
10476058	Tgm6	NM_177726	0.00146813
10358459	BC003331	NM_145511	0.00146966
10408144	V1rh9	NM_134218	0.0014706
10521964	Gm10440	ENSMUST000001	0.00147165
10544608		---	0.00147206
10554789	Ctsc	NM_009982	0.00147299
10545409	Vamp8	NM_016794	0.00147353
10450957	Cenpq	NM_031863	0.001474
10454326	2700062C07Rik	BC084681	0.00147442
10584481	Gm8543	XR_033953	0.00147474
10474984	Nusap1	NM_133851	0.00147623
10412667	Ptprg	NM_008981	0.00148009
10563116	Flt3l	NM_013520	0.0014819
10598287	LOC100270707	NR_026741	0.00148749
10517559	Cdc42	NM_009861	0.00148848
10582466	Sult5a1	NM_020564	0.00149034
10552418	Etfb	NM_026695	0.00149148
10526459	Rasa4	NM_133914	0.00149306
10497399	Pde7a	NM_001122759	0.00149395
10510914	B230396O12Rik	NM_172878	0.00149418
10514333	4930553M12Rik	ENSMUST000000	0.0014972
10417124	B930095G15Rik	BC096543	0.00149848
10339750		---	0.00150107
10538115	Gimap8	NM_001077410	0.00150153
10461369	Ahnak	NM_009643	0.00150689
10575750	Bcmo1	NM_021486	0.00150797
10375903	Cdkn2aipnl	NM_029976	0.00151295
10575702	Clec3a	NM_001007223	0.00151557
10349102	Bcl2	NM_009741	0.00151646
10472846	Pdk1	NM_172665	0.00151842
10601598	3110007F17Rik	BC027572	0.00152131
10604019	1810037I17Rik	NM_024461	0.00152196
10345141	Lmbrd1	NM_026719	0.00152228
10499121	Rps3a	NM_016959	0.00152401
10469250		---	0.00152443
10436239	2310061J03Rik	NR_027965	0.00152501
10581049	Cmtm2a	NM_027022	0.00152635
10395684	Nubpl	NM_029760	0.00153367
10351138	Gm7549	XR_031476	0.00153377
10438907	Gp5	NM_008148	0.00153544
10436708	Usp16	NM_024258	0.00153557
10608706		---	0.00153635
10414449	Mudeng	NM_144535	0.00153761
10419296	Wdhd1	NM_172598	0.00153769
10364262	Itgb2	NM_008404	0.00153771
10440572	Rnf160	NM_001081068	0.00153904
10607300	Gm10437	ENSMUST000001	0.00154409
10503617	F730047E07Rik	NM_199467	0.00154422
10450161	H2-Ea	NM_010381	0.00154431
10398360		---	0.001545
10495193	Kcna2	NM_008417	0.00154602
10476653	Banf2	NM_207275	0.00154604
10385391	Cyfip2	NM_133769	0.00154632
10488816	Ahcy	NM_016661	0.00154813
10342777		---	0.00154836
10426507	Arid2	NM_175251	0.00154997
10549361	Tm7sf3	NM_026281	0.00155125
10520696	Gm9924	ENSMUST000000	0.0015518
10433445	Abat	NM_172961	0.00155246
10472738	Dcaf17	BC031546	0.00155564
10339358		---	0.00155652
10517328	Tmem50a	NM_027935	0.00155925
10517443	Tceb3	NM_013736	0.00156206
10543358	Gm5301	XM_621704	0.00156583
10601559		---	0.00156677
10591161	Zfp558	ENSMUST000000	0.00156825
10403394	Gm5191	XR_031052	0.00156958
10531887	Slc10a6	NM_029415	0.00157124
10562096	Tmem147	NM_027215	0.00157207
10574595		---	0.00157229
10540273	Ube2v2	NM_023585	0.00157692
10437594	Usp7	NM_001003918	0.00158027
10400630		---	0.00158113
10369748	Gm7075	ENSMUST000000	0.00158194
10379615	Slfn5	NM_183201	0.00158229
10551736	Ppp1r14a	NM_026731	0.00158368
10504398	Serf2	NM_011354	0.00158683
10442493		---	0.0015873
10526838	Got2	NM_010325	0.00159114
10500876	Lrig2	NM_001025067	0.00159207
10474725	Eif2ak4	NM_013719	0.00159674
10572747	Tpm4	NM_001001491	0.0015994
10420823	Hmbox1	NM_177338	0.00160381
10597182	Nbeal2	BC157956	0.0016047
10379689	Taf15	NM_027427	0.00160718
10565381		---	0.00160759
10508382	Ak2	NM_016895	0.00161309
10583090	Mmp10	NM_019471	0.00161337
10574023	Mt2	NM_008630	0.00161994
10466423	Cep78	NM_198019	0.00162045
10372226	Myf5	NM_008656	0.00162071
10506301	Lepr	NM_001122899	0.00162193
10604620	Fam122b	NM_030167	0.00162243
10492078	Alg5	NM_025442	0.00162378
10441511			0.00162399
10436428	Mina	NM_025910	0.0016252
10339491		---	0.00162527
10590972	Gm16379	NM_001111330	0.00162538
10567216	Rps15a	NM_170669	0.00162756
10591739	Acp5	NM_001102404	0.00162781
10576816	Cd209b	NM_026972	0.00162909
10446693	Wdr43	NM_175639	0.00163016
10587733	Ctsh	NM_007801	0.0016314
10585444	Ireb2	NM_022655	0.00163165
10342709		---	0.00163267
10577641	1810011O10Rik	NM_026931	0.00163274
10587534	Bckdhb	NM_199195	0.00163415
10408616	Slc22a23	NM_001033167	0.00163459
10505299	Bspry	NM_138653	0.00163855
10513195	Txn1	NM_011660	0.00163909
10476880	A530006G24Rik	XR_034866	0.00164168
10527441	Arpc1b	NM_023142	0.00164626
10485314	Ttc17	NM_183106	0.00164805

10396141		---	0.0016481
10487267	Polr2l	NM_025593	0.00164827
10362904	Rtn4ip1	NM_130892	0.00164871
10558029	Sec23ip	NM_001029982	0.0016525
10357833	Atp2b4	NM_213616	0.00165292
10528972	Otof	NM_031875	0.00165294
10596583	Dock3	NM_153413	0.00165591
10595046	Gm3671	XR_034893	0.00165643
10374983	Asb3	NM_023906	0.00165741
10372807	Msrb3	NM_177092	0.00165746
10447038	1110001A16Rik	ENSMUST00000(	0.00166203
10397094	Psen1	NM_008943	0.00166312
10479335	Osbpl2	NM_144500	0.00166316
10563659	Spty2d1	NM_175318	0.00166362
10422280	Abcc4	NM_001033336	0.00166778
10380571	Gngt2	NM_023121	0.00166804
10501608	Vcam1	NM_011693	0.00166903
10458983	03-Mar	NM_177115	0.00166925
10389190	Gm11435	NM_001045543	0.00167076
10516371	Eif2c1	NM_153403	0.00167317
10446376	Man2a1	NM_008549	0.00167332
10342526		---	0.00167508
10499996	Snx27	NM_029721	0.00167547
10571170	Chrnb3	NM_173212	0.00167745
10352192	Ahctf1	NM_026375	0.00167798
10531987	Gbp4	NM_008620	0.00167856
10565083	Rps17	NM_009092	0.00167896
10338047		---	0.00168052
10446027	Chaf1a	NM_013733	0.00168156
10565437	Tmem135	NM_028343	0.00168352
10529226	Rbks	NM_153196	0.001684
10567355	Gprc5b	NM_022420	0.00168476
10406898	Taf9	NM_027592	0.00168839
10355176	4921521F21Rik	BC051128	0.00169226
10376324	Gm12250	NM_001135115	0.00169278
10399360	Rhob	NM_007483	0.0016929
10390748	Tns4	NM_172564	0.00169314
10368883	Tdg	NM_011561	0.00169353
10378367	Trpv3	NM_145099	0.00169415
10385832	Il4	NM_021283	0.00169466
10428619	Enpp2	NM_015744	0.00169896
10484739	Olfr1179	NM_146917	0.0016999
10478077	B230339M05Rik	NM_177658	0.00170037
10395855	Gm5081	NM_001037746	0.00170233
10483679	Gpr155	NM_001080707	0.00170411
10437399	Coro7	NM_030205	0.00170419
10492480	Gfm1	NM_138591	0.0017059
10596880	Bsn	NM_007567	0.00170639
10569200	Polr2l	NM_025593	0.0017067
10405783		---	0.00171238
10440300		---	0.00171316
10520506	Kcnk3	NM_010608	0.00171642
10411519	Mtap1b	NM_008634	0.00171717
10351966	Olfr414	NM_146761	0.00171811
10444298	H2-Eb1	NM_010382	0.00172398
10576027	Gm10614	ENSMUST00000(	0.00172889
10513824	Cdk5rap2	NM_145990	0.00172969
10405822	Ccrk	NM_053180	0.00173039
10410970	Hnrnpk	NM_025279	0.00173057
10406205	Erap1	NM_030711	0.00173432
10340053		---	0.00174161
10548030	Cd9	NM_007657	0.00174381
10590648	Top2a	NM_011623	0.00174518
10358389	Rgs2	NM_009061	0.00174531
10558118	Btbd16	NM_001081038	0.00174626
10501222	Gstm2	NM_008183	0.00174692
10568417	1110007A13Rik	BC038342	0.00174881
10418129		---	0.00175154
10593591	Acat1	NM_144784	0.00175291
10470893	Set	NM_023871	0.00175473
10370754	Dos	NM_015761	0.00175532
10372687	Nup107	NM_134010	0.00175941
10601539		---	0.00176012
10380087	Mtmr4	NM_133215	0.00176067
10378579	Prpf8	NM_138659	0.00176134
10476401	Plcb1	NM_019677	0.00176165
10571889		---	0.0017626
10412376	Emb	NM_010330	0.00176391
10458130	4933408B17Rik	NM_177773	0.00176598
10554704	Mesdc2	NM_023403	0.00176683
10437023	Morc3	NM_001045529	0.00177144
10497214	Tpd52	NM_001025261	0.00177261
10515737	BC059842	FJ998170	0.00177283
10340658		---	0.00177592
10475845	Acoxl	NM_028765	0.00177647
10503966	Aco1	NM_007386	0.00177777
10409990	6720489N17Rik	BC053725	0.00177865
10454015	Ttc39c	NM_028341	0.00177906
10571653	Actg1	NM_009609	0.0017791
10383208		---	0.00178027
10555716	Olfr547	NM_147079	0.00178165
10341666		---	0.0017818
10603323	Ppp1r3f	NM_138605	0.00178274
10523563	Cds1	NM_173370	0.001783
10392440	Slc16a6	NM_001029842	0.00178363
10606835	Bex2	NM_009749	0.00178553
10583905	07-Sep	NM_009859	0.00178948
10450904	Scoc	NM_001039137	0.00179142
10541599	Clec4b2	NM_001004159	0.00179192
10388749	Traf4	NM_009423	0.00179257
10481577	Uck1	NM_011675	0.00179274
10543676	1700080G18Rik	ENSMUST00000(	0.0017937
10571093	Rnf170	NM_029965	0.00179384
10392241	Polg2	NM_015810	0.0017951
10599416	Gm10483	ENSMUST000001	0.00179632
10439463	4930455C21Rik	NM_024273	0.00179633
10371907	Nedd1	NM_008682	0.00179701
10365792	Ccdc38	NM_175488	0.00179792
10591127	Fat3	NM_001080814	0.00180102
10397912	Unc79	NM_001081017	0.00180338
10512766	Trim14	NM_029077	0.00180594
10450363	Snord52	AF357375	0.00180677
10406108	5430425J12Rik	ENSMUST00000(	0.00180696
10506870	Txndc12	NM_025334	0.00180841
10580457	N4bp1	NM_030563	0.00181088
10601648	Tnmd	NM_022322	0.00181303
10584580		---	0.0018147
10513166	Ptpn3	NM_011207	0.00181709
10493516	Gm15417	ENSMUST00000(	0.00181965
10467124	Acta2	NM_007392	0.00182342
10565547	Pcf11	NM_029078	0.0018236
10509992	Hspb7	NM_013868	0.00182527
10488048	Mkks	NM_021527	0.00182943
10455461	Myot	NM_001033621	0.0018299
10447483	Nanp	NM_026086	0.00183188
10563447	Abcc6	NM_018795	0.00183581
10434758	St6gal1	NM_145933	0.00183708
10352864		---	0.0018378

10374476	Rps17	NM_009092	0.00183938
10520467	Hadhb	NM_145558	0.00184029
10339856		---	0.00184084
10421046	Dock5	NM_177780	0.00184563
10458808	Fem1c	NM_173423	0.00184602
10594988	Mapk6	NM_015806	0.00184611
10453759	Gm10554	ENSMUST00000(	0.00184637
10429460	7120482A17Rik	AK149087	0.00184644
10447742	Map3k4	NM_011948	0.0018482
10341502		---	0.00184954
10377534	A030009H04Rik	NR_027827	0.00185122
10488108	Esf1	NM_001081090	0.00185129
10349378	Dpp10	NM_199021	0.00185341
10591618	Dock6	NM_177030	0.00185392
10567863	Cd19	NM_009844	0.00185407
10579776	Arhgap10	NM_030113	0.00185509
10366956	Stat6	NM_009284	0.00185576
10573194	Ndufb7	NM_025843	0.00185757
10344278		---	0.00186064
10482866	Tanc1	NM_198294	0.00186172
10403453	Dip2c	NM_001081426	0.00186488
10412960	Fam149b	BC069846	0.00186647
10338442		---	0.00186774
10503184	Chd7	NM_001081417	0.0018736
10598839	Rp2h	NM_133669	0.00187478
10507699	Scmh1	NM_013883	0.00187517
10555401		---	0.00187975
10545623	Dok1	NM_010070	0.00188069
10606730	Armcx6	NM_001007578	0.00188101
10567442	Dnahc3	XM_355934	0.00188499
10566846	Dennd5a	NM_021494	0.00188536
10408810		---	0.00188568
10555055	Ndufc2	NM_024220	0.00188692
10596575	Manf	NM_029103	0.00189162
10510882	Megf6	NM_001162977	0.00189269
10387699	Centb1	NM_153788	0.00189361
10600892	Pgr15l	NM_001033361	0.00189505
10402490	4831426I19Rik	NM_001042699	0.0018978
10528120	Dmtf1	NM_011806	0.00190241
10526261	Stx1a	NM_016801	0.00190668
10407598	Ryr2	NM_023868	0.00190813
10345077	Khdc1a	NM_183322	0.00191103
10411126	Jmy	NM_021310	0.00191296
10411711	Cdk7	NM_009874	0.00191378
10553413	Prmt3	NM_133740	0.00191525
10608645		---	0.00192032
10480477	Pax8	NM_011040	0.00192341
10499062	Fhdc1	NM_001033301	0.00192703
10412773	Slc4a7	NM_001033270	0.0019298
10506883	Rab3b	NM_023537	0.00193
10584374	Olfr888	NM_146424	0.00193027
10549162	St8sia1	NM_011374	0.00193208
10400609	Mdga2	NM_207010	0.00193431
10563149	Ccdc155	NM_201374	0.00193584
10556491	Far1	NM_026143	0.00193768
10380558		---	0.00193812
10342010		---	0.00193841
10502774	Lphn2	NM_001081298	0.00193968
10342160		---	0.00194173
10442231		---	0.00194235
10468413	D19Ertd652e	BC107403	0.00194432
10587778		---	0.00195411
10341351		---	0.00195532
10566201	Olfr589	NM_147052	0.00195812
10402444	Dicer1	NM_148948	0.00196153
10355024	Ica1l	NM_027407	0.00196253
10557754	Orai3	NM_198424	0.0019627
10473650	Nup160	NM_021512	0.00196288
10394749	Nol10	NM_001008421	0.00196416
10407782	Edaradd	NM_133643	0.00196987
10459837	8030462N17Rik	BC120889	0.00197043
10544732	Skap2	NM_018773	0.00197252
10480459	Hnmt	NM_080462	0.00197322
10344023		---	0.00197477
10536334	Dync1i1	NM_010063	0.00197797
10421100	Nefm	NM_008691	0.00197811
10580807	Kifc3	NM_010631	0.00197914
10338002		---	0.00197926
10421877	Diap3	NM_019670	0.00198125
10405147		---	0.00198204
10526514	Cldn15	NM_021719	0.00198369
10565589	Hmgb1	NM_010439	0.00198555
10400052	4930579E17Rik	NM_178629	0.00198757
10386582	Top3a	NM_009410	0.00198851
10598004	Ccr1	NM_009912	0.00198978
10416215	Loxl2	NM_033325	0.0019919
10408741	Txndc5	NM_145367	0.00199439
10518228	Vps13d	NM_001128198	0.00199475
10574682	E2f4	NM_148952	0.00200371
10390895	Krtap3-1	ENSMUST00000(	0.00200797
10600453	Ikbkg	NM_001161421	0.00200858
10415413	BC030046	BC151043	0.00201059
10413280		---	0.00201157
10539080	St3gal5	NM_011375	0.0020174
10604248	Thoc2	NM_001033422	0.00201871
10389421	Ints2	NM_027421	0.00201987
10588479	Tlr9	NM_031178	0.00202002
10562927	Ptov1	NM_133949	0.00202014
10404049	Hist1h3g	NM_145073	0.0020236
10560043	Zfp329	NM_026046	0.00202575
10440570	Rnf160	NM_001081068	0.00203056
10340568		---	0.00203134
10395594		---	0.0020338
10522051	Klf3	NM_008453	0.00203592
10460177	Mtl5	NM_001039657	0.00203629
10571680	Mlf1ip	NM_027973	0.00203948
10503709	D130062J21Rik	AK051661	0.00204156
10406817	Enc1	NM_007930	0.00204276
10404521	Bphl	NM_026512	0.00204588
10489784	Prex1	NM_177782	0.00204975
10356510	Iqca	ENSMUST000001	0.00205023
10442542	Nthl1	NM_008743	0.00205283
10359648	Scyl3	NM_028776	0.00205291
10579825	Pou4f2	NM_138944	0.00206225
10534202	Ncf1	NM_010876	0.00206332
10394682	LOC633944	XR_032386	0.00206477
10377982	Kif1c	NM_153103	0.00206597
10459075	Myoz3	NM_133363	0.00206917
10496169	Ppa2	NM_146141	0.00206965
10455942	A730017C20Rik	BC075669	0.00207213
10355266	Lancl1	NM_021295	0.0020744
10606439		---	0.00207535
10553450	Nell1	NM_001037906	0.00207603
10549420	Tmtc1	NM_198967	0.00207636
10372099		---	0.00208123
10577144	Dcun1d2	NM_001024504	0.00208153
10409978	Gm7036	NM_001039239	0.00208656
10540298	Chl1	NM_007697	0.00208751

10498871	Tmem144	NM_027495	0.00208847
10339713		---	0.00209029
10543065		---	0.00209609
10466935	Rln1	NM_011272	0.00209741
10547088	Mbd4	NM_010774	0.0021009
10502823	Dnajb4	NM_025926	0.00210116
10421723	Dnajc15	NM_025384	0.00210127
10428074	Rpl30	NM_009083	0.00210131
10568150	Kif22	NM_145588	0.00210213
10399461	Gm4929	XR_031578	0.00210309
10545502	Dnahc6	ENSMUST00000	0.00210555
10457040	Zfp516	NM_183033	0.0021064
10591884	Glb1l2	NM_153803	0.00210691
10427918	Fam105a	BC052328	0.00211519
10343560		---	0.00211985
10466920	Slc1a1	NM_009199	0.00212129
10412711	Uqcrb	NM_026219	0.00212473
10421624	Cog3	NM_177381	0.00212601
10458090	Reep5	NM_007874	0.00212601
10339874		---	0.00212614
10375245	Gabrb2	NM_008070	0.00213035
10374746	Fancl	NM_025923	0.00213061
10500103	Gabpb2	NM_029885	0.00213135
10512291	Dctn3	NM_016890	0.00213174
10605884	Ophn1	NM_052976	0.00213271
10342064		---	0.00213793
10603247		---	0.00214298
10439932		---	0.00214547
10397818	Cpsf2	NM_016856	0.00214658
10519211	Tas1r3	NM_031872	0.0021468
10340343		---	0.00214727
10338774		---	0.00214863
10465804	Polr2g	NM_026329	0.00214967
10362811	Sesn1	NM_001013370	0.00215032
10385599	Canx	NM_007597	0.00215222
10341934		---	0.00215593
10339360		---	0.00215801
10578902		---	0.00215931
10352292	G370120E05Rik	ENSMUST00000	0.00216397
10354788		---	0.0021667
10408629	1300014I06Rik	NM_025831	0.00216767
10507347	Tesk2	NM_146151	0.00216872
10497663	Slc7a14	NM_172861	0.0021716
10437817	Pdxdc1	NM_053181	0.00217285
10434643	Psmb3	NM_011971	0.00217326
10582337	Fam38a	ENSMUST00000	0.00217529
10419999	Jph4	NM_177049	0.00217529
10406795	Gfm2	NM_177266	0.00217531
10593605	Cul5	NM_027807	0.00217549
10358551	Hmcn1	NM_001024720	0.00217752
10514296	Ifna9	NM_010507	0.00217782
10468016	Ndufb8	NM_026061	0.00217899
10433433	BC024814	BC024814	0.00218051
10490826	Zbtb10	NM_177660	0.00218295
10468533	Gpam	NM_008149	0.00218518
10388869	Tnfaip1	NM_009395	0.00218634
10431935	Amigo2	NM_178114	0.00218652
10338645		---	0.00218657
10484207	2610301F02Rik	NM_001025576	0.00218957
10601235	Ogt	NM_139144	0.00219118
10574765	Lrrc36	NM_001033371	0.00219372
10576829	Cd209c	NM_130903	0.00219481
10544150	Jhdm1d	NM_001033430	0.00219552
10503989	2010003O02Rik	ENSMUST00000	0.00219649
10430745	Chadl	ENSMUST00000	0.00220096
10576971	Irs2	NM_001081212	0.00220199
10603627	Bcor	NM_175045	0.00220327
10345091	B3gat2	NM_172124	0.00220383
10494407	Hist2h2bb	NM_175666	0.00220533
10423017		---	0.00221017
10376383	Gm12253	NM_001045542	0.00221935
10496580	Gbp3	NM_018734	0.00222069
10603440	Was	NM_009515	0.00222098
10600733	Gm5072	NM_001114678	0.00222112
10494390	Hist2h2aa1	NM_013549	0.00222374
10598055	ND3	ENSMUST00000	0.00222453
10407709	Mtr	NM_001081128	0.00222963
10468489	Xpnpep1	NM_133216	0.00223354
10576439	Cog2	NM_029746	0.00223447
10548931	Slc15a5	NM_177787	0.002237
10391013	Krt13	NM_010662	0.00223829
10577395	6820431F20Rik	BC058969	0.00223853
10432820	Krt72	NM_213728	0.00223913
10537026	Cpa4	NM_027926	0.00224108
10528723	Mll3	NM_001081383	0.0022428
10426180	Saps2	NM_026813	0.00224299
10338048		---	0.00224738
10353167	Tram1	NM_028173	0.00225155
10596982	Ccdc72	NM_183250	0.00225285
10548565	Magohb	NM_025564	0.00225313
10545346	Ptcd3	NM_027275	0.00225392
10339247		---	0.00225754
10540059	Slc41a3	NM_027868	0.00225808
10517924	Clcnkb	NM_019701	0.00225944
10461844	Gnaq	NM_008139	0.00226186
10467859	Cox15	NM_144874	0.00226513
10521222	Add1	NM_001102444	0.00226567
10408130	V1rh15	ENSMUST00000	0.00226988
10465411	Slc22a12	NM_009203	0.00227048
10399677	Cox7a2l	NM_001159529	0.00227544
10478145	Ppp1r16b	NM_153089	0.00227733
10595466	Pgm3	NM_028352	0.00228063
10376283		---	0.00229074
10462822	Exoc6	NM_175353	0.00229162
10499514	Trim46	NM_183037	0.00229201
10389507	Tmem49	NM_029478	0.00229359
10354868	Fam126b	NM_172513	0.00229805
10344270		---	0.00229871
10413419	Arhgef3	NM_027871	0.00230294
10434418	Vwa5b2	NM_182636	0.00230513
10449163	Pigq	NM_011822	0.00230561
10535750	Mtif3	NM_029581	0.00230584
10368647	Dse	NM_172508	0.00230689
10570483	Arhgef10	NM_172751	0.00230775
10494395	Hist2h2aa1	NM_013549	0.00231047
10343488		---	0.00231369
10483131	Kcnh7	NM_133207	0.00231533
10603328	Ccdc22	NM_138603	0.00232041
10516566	C77080	NM_001033189	0.00232193
10489127	Rbl1	NM_011249	0.0023224
10490872	Lrrcc1	NM_028915	0.00232747
10382228	Axin2	NM_015732	0.00232818
10500582	Wdr3	NM_175552	0.00233065
10503305	1110037F02Rik	BC028830	0.00233126
10545588	Hk2	NM_013820	0.00233219
10360506	Akt3	NM_011785	0.00233634
10558903	Taldo1	NM_011528	0.0023378
10496302	Manba	NM_027288	0.00234303

10511881	Manea	NM_172865	0.00234617
10561178	Rab4b	NM_029391	0.00234806
10593953	Lman1l	NM_199222	0.00234827
10338347		---	0.00234898
10349562	AA986860	NM_177604	0.00235082
10598049	ND3	ENSMUST00000(	0.00235591
10436304	Abi3bp	NM_001014423	0.00235816
10373826	Gal3st1	NM_016922	0.00235922
10441231	Bace2	NM_019517	0.00236066
10493565	Adar	NM_001038587	0.00236099
10445894	Erh	NM_007951	0.00236123
10449979	Morc2b	NM_177719	0.00236291
10465303	Slc22a20	NM_198650	0.00236818
10393058	H3f3b	NM_008211	0.00237032
10569311	Gm5054	XR_034827	0.0023721
10492890	Lrba	NM_030695	0.00237242
10540650	Arpc4	NM_026552	0.00237368
10530096		---	0.00237394
10532905	4930519G04Rik	NM_026263	0.00237635
10341749		---	0.00237669
10597978	Fyco1	NM_148925	0.0023797
10343735		---	0.00238053
10445688	Ccnd3	NM_001081636	0.00238157
10506743	0610037L13Rik	BC019215	0.00238266
10463695	2010012O05Rik	BC027506	0.00238347
10408202	Hist1h3f	NM_013548	0.00238393
10598057		---	0.00239743
10557308	Hs3st4	ENSMUST000001	0.00239943
10597758	Csrnp1	NM_153287	0.00239984
10366517	Kcnmb4	NM_021452	0.00240078
10537817	Olfr441	NM_146655	0.00240212
10587688		---	0.00240357
10595620		---	0.00240357
10487787	Gfra4	NM_020014	0.00240393
10363195	Hsf2	NM_008297	0.00240635
10512915	2810432L12Rik	NM_025944	0.00241037
10451953	Lrg1	NM_029796	0.00241255
10579731	Med26	NM_027485	0.0024132
10503464	Cngb3	NM_013927	0.00241483
10533246	Oas1g	NM_011852	0.00241778
10517646	Pla2g2f	NM_012045	0.00241836
10567446	Dnahc3	BC051401	0.00242001
10514561	E130114P18Rik	ENSMUST00000(	0.00242101
10482123	Olfr351	NM_146942	0.00242178
10340767		---	0.00243004
10420426	F630043A04Rik	NM_198605	0.00243041
10385447	Dppa1	NM_178247	0.00243307
10604564	Gpc4	NM_008150	0.00243649
10346564	Casp8	NM_009812	0.00243862
10569291	Krtap5-2	NM_027844	0.00244224
10553833	Ndn	NM_010882	0.0024427
10593671	Dmxl2	NM_172771	0.0024432
10430993	1700001L05Rik	ENSMUST000001	0.00244363
10487380	Gm10766	ENSMUST00000(	0.00245148
10445458	Xpo5	NM_028198	0.00245233
10449672	Tmprss3	NM_080727	0.00245544
10534990	Taf6	NM_009315	0.00245705
10342664		---	0.00245813
10583163	Trpc6	NM_013838	0.00246449
10418455	Itih1	NM_008406	0.00246502
10438445	Klhl6	NM_183390	0.00246614
10338403		---	0.00246878
10355532	Tns1	NM_027884	0.00246965
10357242	Dbi	NM_001037999	0.00246974
10408083	Hist1h3g	NM_145073	0.00247058
10554569	Fam103a1	BC096399	0.00247467
10344044		---	0.0024752
10378126	Ankfy1	NM_009671	0.00247793
10451077		---	0.00248157
10449207	Rab11fip3	NM_001162869	0.00248541
10489355	Jph2	ENSMUST00000(	0.00248869
10430748	Rangap1	NM_011241	0.00249183
10338028		---	0.00249559
10413174	Rps24	NM_011297	0.00249716
10439218	Pdia5	NM_028295	0.00249938
10540639	Ogg1	NM_010957	0.00250056
10439780	6430553K19Rik	ENSMUST00000(	0.00250095
10462603	Fas	NM_007987	0.00250202
10498620	Trim59	NM_025863	0.00250205
10406905	Ccdc125	NM_183115	0.00250361
10340605		---	0.00250369
10416437	Lcp1	NM_008879	0.00250387
10408613	Tubb2b	NM_023716	0.00250479
10434446	Ece2	NM_139293	0.00250742
10362350	Themis	NM_178666	0.00250925
10568225	Zfp768	NM_146202	0.00251282
10364728	Mum1	NM_023431	0.00251288
10406067	Clptm1l	NM_146047	0.00251332
10468513	1700001K23Rik	ENSMUST00000(	0.00251426
10447294	Prkce	NM_011104	0.00251487
10444258	Psmb8	NM_010724	0.0025217
10399924	Pik3cg	NM_020272	0.0025221
10549375	Rps26	NM_013765	0.00252213
10400479	Nkx2-9	NM_008701	0.00252412
10384737	Papolg	NM_172555	0.00252428
10490838	Fabp5	NM_010634	0.00252673
10374485	Peli1	NM_023324	0.00253128
10595626		---	0.00253271
10484425	2700094K13Rik	NM_001033166	0.0025332
10464642	Atpgd1	BC023699	0.00253374
10459329	2700046A07Rik	ENSMUST00000(	0.00253504
10393591		---	0.00253642
10442719	Ccdc154	ENSMUST00000(	0.00254167
10460591	Fibp	NM_021438	0.00254186
10444788	H2-Q1	NM_010390	0.00254584
10455531	1700018A14Rik	NM_001145259	0.00254701
10396712	Fut8	NM_016893	0.00255039
10499963	Tdpoz1	NM_148949	0.00255157
10557177	Prkcb	NM_008855	0.00255288
10492195	Tsc22d2	NM_001081229	0.002553
10411078	Spz1	NM_030237	0.00255363
10589940	Osbpl10	NM_148958	0.0025538
10546349	Xpc	NM_009531	0.00255387
10381474	Arl4d	NM_025404	0.00255915
10340059		---	0.0025623
10546180	Gm839	BC147090	0.00256291
10588035	Gm8560	XR_031229	0.00256677
10338065		---	0.00256774
10442258	4930432O21Rik	NM_001025373	0.00256995
10531583	1700010H22Rik	ENSMUST000001	0.00257134
10459066	LOC100044195	BC150900	0.00257213
10426767	Aqp5	NM_009701	0.00257509
10599342	6030498E09Rik	NM_183126	0.00257588
10593219	Nnmt	NM_010924	0.00257617
10503410	Tmem64	NM_181401	0.00258408
10475427	4933406J08Rik	NM_028914	0.00258631
10481135	Surf1	NM_013677	0.00259763
10606714	Gla	NM_013463	0.00260136

10599776	Brs3	NM_009766	0.00260678
10570556	Mcph1	NM_173189	0.00260724
10371082	Itgb1bp3	NM_027120	0.00260934
10339654		---	0.00261091
10419261	Bmp4	NM_007554	0.00261286
10454093	Mrpl27	NM_053161	0.00261436
10420497	Gm6907	EU155106	0.00261559
10432439	Fmnl3	NM_011711	0.00262151
10424404	Pvt1	NR_003368	0.00262362
10437885	Myh11	NM_013607	0.00263449
10434577	Vps8	NM_001081366	0.00263583
10412207	Gpx8	NM_027127	0.00263752
10576532	Tsnax	NM_016909	0.00264419
10341908		---	0.00264492
10443095	Syngap1	ENSMUST00000(	0.00264674
10344895	Gm7560	XR_032183	0.00264858
10517677	Nbl1	NM_008675	0.00264867
10374793	Pnpt1	NM_027869	0.00264979
10466841		---	0.0026627
10453178	Map4k3	NM_001081357	0.00266651
10472469	4932414N04Rik	NM_183113	0.00266831
10514338		---	0.00267369
10374929	4931440F15Rik	BC139107	0.00267663
10469167	Sfmbt2	NM_177386	0.00267695
10341587		---	0.00268254
10450694	H2-T22	NM_010397	0.00268341
10554240	Isg20	NM_020583	0.00268483
10599498	Utp14a	NM_028276	0.00268515
10586184	Tipin	NM_025372	0.00269079
10500990	Atp5f1	NM_009725	0.00269103
10546960		---	0.00269468
10496338	Ppp3ca	NM_008913	0.00269707
10422598	Sepp1	NM_009155	0.00269789
10539669	Add2	NM_013458	0.00270026
10593490	Gm6981	NR_023357	0.00270047
10556082	Ppfibp2	NM_008905	0.00270257
10504646		---	0.00270643
10353947	Tmem131	NM_018872	0.00270715
10339238		---	0.00270787
10397351	Jdp2	NM_030887	0.00270941
10340927		---	0.00271364
10574694	Elmo3	NM_172760	0.00271372
10388884	Nlk	NM_008702	0.00271374
10339504		---	0.00271799
10344010		---	0.00271803
10370007	Gstt4	NM_029472	0.00272352
10494170	Lysmd1	NM_153121	0.00272501
10427492	Gm10050	ENSMUST00000(	0.00272528
10528951	Gpr113	NM_001014394	0.00272855
10338136		---	0.0027293
10402266		---	0.00273046
10544702	Gm5301	XM_621704	0.00273205
10397752	Calm1	NM_009790	0.00273218
10540241	Arl6ip5	NM_022992	0.00273269
10376868	Trpv2	NM_011706	0.00273328
10506594	Acot11	NM_025590	0.0027387
10369388	Unc5b	NM_029770	0.0027391
10418921	Sncg	NM_011430	0.00273911
10538503	Ccdc129	NM_001081665	0.00273985
10519655	Gm8912	XR_002114	0.00274091
10491945	Rab33b	NM_016858	0.00274117
10514466	Jun	NM_010591	0.00274206
10414720		---	0.00274653
10414917		---	0.00274653
10531201	Adamts3	NM_001081401	0.00274681
10440028	2310005G13Rik	NM_183281	0.00274847
10466659	Gda	NM_010266	0.00274865
10338325		---	0.00274895
10561085	Hnrnpul1	NM_144922	0.0027494
10513943	Rasef	ENSMUST000001	0.00275141
10378523	Smg6	NM_001002764	0.00275587
10581073	Dync1li2	NM_001013380	0.0027576
10362102	Gm10825	ENSMUST000001	0.00275863
10385155	Fbll1	NM_001004147	0.00276272
10373912	Osm	NM_001013365	0.0027628
10489261	Emilin3	NM_182840	0.002764
10572596	5430437P03Rik	BC005692	0.00276405
10372082	Nudt4	NM_027722	0.00276655
10525885	Ubc	BC025894	0.00276714
10376803	Fam83g	NM_178618	0.00276895
10457929	Rit2	NM_009065	0.00277017
10410134	Gm7712	XR_033575	0.00277068
10429407		---	0.00277105
10366344	Gm5176	BC054110	0.00277134
10565072	Sec11a	NM_019951	0.00277418
10389719	Scpep1	NM_029023	0.00277639
10485955	Scg5	NM_009162	0.00277689
10351843	Vsig8	NM_177723	0.00278107
10387651	4933402P03Rik	BC137961	0.00279096
10420390	Xpo4	NM_020506	0.00279155
10582922		---	0.00279575
10397148	Acot1	NM_012006	0.00279629
10584162	Tmed2	NM_019770	0.00279668
10338823		---	0.00279792
10355836	Resp18	NM_009049	0.00279993
10342437		---	0.00280083
10488785	E2f1	NM_007891	0.00280603
10358648	Hmcn1	NM_001024720	0.00280626
10596277	Dnajc13	NM_001163026	0.00280628
10421709	Gm6994	NM_001037935	0.00280809
10463630		---	0.00281282
10379153	Aldoc	NM_009657	0.00281568
10489422	Kcns1	NM_008435	0.00281579
10440977	Atp5o	NM_138597	0.00281778
10514926	B230314M03Rik	ENSMUST00000(	0.00281792
10531415	Cxcl10	NM_021274	0.00282012
10416099	Adra1a	NM_013461	0.00282029
10353450	Gm4956	NR_002858	0.0028203
10576328	Mc1r	NM_008559	0.00282281
10422259	Tgds	NM_029578	0.00282343
10521415	Ablim2	NM_177678	0.00282346
10340591		---	0.00282582
10356999	Prdx2	NM_011563	0.00282683
10519105	Ski	NM_011385	0.00282842
10473576	Olfr1152	NM_001011834	0.00282925
10384577		---	0.00283029
10484811	Olfr1232	NM_146323	0.00283186
10431637	Cpne8	NM_025815	0.00283315
10403941	Hist1h3g	NM_145073	0.00283737
10383993	Ccdc117	NM_134033	0.00283904
10472930	Sp9	NM_001005343	0.00284232
10343067		---	0.00284297
10416940	Tpm3	ENSMUST00000(	0.00284983
10412443	Cts3	NM_026906	0.0028507
10409955	Cts3	NM_026906	0.00285074
10552752	Akt1s1	NM_026270	0.00285314
10554419	Vps33b	NM_178070	0.00285391
10557148	Dctn5	NM_021608	0.00285447

10529711	Clnk	NM_013748	0.00286007
10344165		---	0.00286325
10538842	Gng12	NM_025278	0.00286383
10594277	Paqr5	NM_028748	0.00286519
10598178	Disp1	NM_026866	0.00286577
10381870	Gm10842	ENSMUST00000	0.00286641
10449971	Zfp763	NM_028543	0.00286826
10534102	Gusb	NM_010368	0.0028712
10395910	Pnn	NM_008891	0.00287135
10476941		---	0.00287256
10494405	Hist2h3b	NM_178215	0.00287626
10405211	Gadd45g	NM_011817	0.00287819
10445434	Mrps18a	NM_026768	0.0028813
10544932	Inmt	NM_009349	0.0028833
10580986	Rbmxrt	NM_009033	0.00288538
10571310	Rpl29	NM_009082	0.00288891
10587554	Tpbg	NM_011627	0.00289337
10576854	Ctxn1	NM_183315	0.00289402
10559486	Lair1	NM_001113474	0.00289749
10581813	Mlkl	NM_029005	0.00289997
10343049		---	0.00290065
10527646	BC028471	ENSMUST00000	0.00290607
10492091	Smad9	NM_019483	0.0029077
10515220	Faah	NM_010173	0.00290987
10343669		---	0.00290989
10594490	Slc24a1	NM_144813	0.00291524
10338845		---	0.00291735
10593634	Elmod1	NM_177769	0.00292181
10529758	Bod1l	NM_001081422	0.00292486
10547758	Emg1	NM_013536	0.0029249
10601595	3110007F17Rik	BC027572	0.00293245
10354504	Gm5976	XR_031113	0.00293591
10350297	Kif14	NM_001081258	0.0029383
10449377	Spdef	NM_013891	0.00294082
10417013	Dnajc3	NM_008929	0.00294222
10417034	Dnajc3	NM_008929	0.00294222
10342484		---	0.00294247
10455656	Hsd17b4	NM_008292	0.00294784
10438060	Igll1	ENSMUST00000	0.0029501
10502655	Cyr61	NM_010516	0.00295148
10585599	Imp3	NM_133976	0.00295249
10386110	Hand1	NM_008213	0.00295273
10394627	Nbas	BC057020	0.00295372
10409557	H2afy	NM_012015	0.00295467
10556938	E130201H02Rik	NR_024324	0.00295865
10409579	Cxcl14	NM_019568	0.00295873
10394366	Atad2b	NM_001099628	0.00295913
10378440		---	0.00296075
10340580		---	0.00296281
10548086	Rad51ap1	NM_009013	0.00296464
10471171	Fubp3	NM_001033389	0.00296494
10436788	Hunk	NM_015755	0.00297037
10498599	Ift80	NM_026641	0.00297106
10389151	Slfn10	NM_181542	0.00297296
10449225	Decr2	NM_011933	0.0029731
10502240	Npnt	NM_033525	0.00297578
10532616	Myo18b	NM_028901	0.00297753
10377265	Pik3r5	NM_177320	0.00297921
10356020	Dock10	NM_175291	0.00297926
10393341	Rhbdf2	NM_172572	0.00298042
10567297	Itpripl2	NM_001033380	0.00298219
10568355	Pycard	NM_023258	0.00298317
10397002	Sipa1l1	NM_172579	0.00298331
10574985	Slc7a6	NM_178798	0.00298509
10404575		---	0.00298669
10394288	Itsn2	NM_011365	0.00299142
10341039		---	0.00299634
10574184	Rspry1	NM_026274	0.00299827
10339824		---	0.00299844
10586168	Snord16a	AF357363	0.00300056
10435226	Snx4	NM_080557	0.0030011
10483604	Slc25a12	NM_172436	0.00300214
10352143	Kif26b	NM_001161665	0.0030045
10488804	Gm14214	XR_031617	0.003005
10358709	Cox7b	NM_025379	0.00300568
10421184	Loxl2	ENSMUST00000	0.0030077
10339205		---	0.00301018
10470349	Adamts13	NM_001001322	0.00301411
10502405	Metap1	NM_175224	0.0030149
10421581	Lrch1	NM_001033439	0.00301891
10607169	Trpc5	NM_009428	0.00301892
10365290	Chst11	NM_021439	0.00301927
10523168	Epgn	NM_053087	0.00302051
10338367		---	0.0030227
10496555	Gbp1	NM_010259	0.0030245
10505779	Acer2	NM_139306	0.003025
10488459	Zfp442	BC023805	0.00302882
10450814	Ppp1r11	NM_029632	0.00303046
10468891	Gm7102	AK163227	0.00303077
10525381	Vps29	NM_019780	0.00303936
10499095	Fam160a1	NM_172682	0.00304277
10385665	Zfp354b	NM_013744	0.00304589
10344922	Gm16393	XR_031240	0.0030488
10507218	Mknk1	NM_021461	0.00305063
10422707	Prkaa1	NM_001013367	0.00305141
10405464	Grk6	NM_001038018	0.00305155
10357886		---	0.00305187
10401343	Map3k9	NM_177395	0.00305197
10344713	Ahcy	NM_016661	0.00305618
10345032	Il17a	NM_010552	0.00305839
10394593	Fam49a	NM_029758	0.0030587
10441864	Mllt4	NM_010806	0.00305872
10451142	Gm7325	DQ190000	0.00306301
10394892	Cpsf3	NM_018813	0.0030632
10378739	Ywhae	NM_009536	0.00306725
10494551	Acp6	NM_019800	0.00306777
10448089	Oaz1	NM_008753	0.0030689
10566333	9230105E10Rik	NM_001146007	0.00307055
10400544	Rpl21	NM_019647	0.00307296
10433963	Ydjc	NM_026940	0.00307551
10478922		---	0.00307579
10451465	Ptcra	NM_011195	0.00307946
10602501	Huwe1	NM_021523	0.00308236
10338463		---	0.0030853
10548207	Pzp	NM_007376	0.00308555
10498827	Fnip2	NM_001162999	0.00308592
10385518	Tgtp	NM_011579	0.00309066
10473507	Olfr1093	NM_146366	0.00309409
10352150		---	0.00309598
10419742	Olfr49	NM_010991	0.00309904
10339894		---	0.00310157
10515028	Zfyve9	NM_183300	0.00311086
10466938	5033414D02Rik	BC024953	0.00311132
10386949	Hmgb1	NM_010439	0.0031141
10454077	Taf4b	NM_001100449	0.00311434
10565759	Uvrag	NM_178635	0.00311482
10344813	Cspp1	NM_026493	0.00311517
10576639	Nrp1	NM_008737	0.00311609

10577065	Gm7606	XR_032000	0.00311909
10367919	Stx11	BC118516	0.00312979
10555262	Xrra1	BC151014	0.00313123
10482249	Nr6a1	NM_010264	0.00313167
10407707	Gm10336	ENSMUST00000(	0.0031321
10341429		---	0.00313394
10403604	Lyst	NM_010748	0.00313841
10581996	Cdyl2	NM_029441	0.00314952
10385966	Anxa6	NM_013472	0.00314996
10410687		---	0.0031537
10456727	Dym	NM_027727	0.00315486
10566597	Olfr698	NM_146602	0.00315687
10343419		---	0.00316009
10467529	Opalin	NM_153520	0.00316016
10443527	Pim1	NM_008842	0.00316058
10422509		---	0.003165
10517301	Ldlrap1	NM_145554	0.00316563
10383819	Sec14l2	NM_144520	0.00316565
10454606	Wdr36	NM_001110015	0.00316608
10376461	Trim11	NM_053168	0.00316832
10552656	Syt3	NM_016663	0.00316848
10414537	Ang	NM_001161731	0.00317137
10392261	Smurf2	NM_025481	0.00317464
10390519	Plxdc1	NM_028199	0.00318405
10366048	B530045E10Rik	ENSMUST00000(	0.00318513
10340528		---	0.00318555
10384219	Gm11977	XR_033922	0.00318564
10376033	Kif3a	NM_008443	0.00318732
10435769	Zbtb20	NM_019778	0.00318824
10499045	Trim2	NM_030706	0.00319481
10404065	Hist1h3f	NM_013548	0.00319594
10544417	Epha1	NM_023580	0.00319695
10545974	Antxr1	NM_054041	0.00319847
10392098	Ftsj3	NM_025310	0.00320542
10538584		---	0.00320667
10408490	Exoc2	NM_025588	0.00320816
10379228	Nos2	NM_010927	0.00320944
10559853	Clcn4-2	NM_011334	0.00321401
10536973	1700023L04Rik	ENSMUST00000(	0.00321526
10515452	Gm1661	NM_001145637	0.00321559
10383212		---	0.00321945
10405918	Rsl1	NM_001013769	0.00322216
10585990	Myo9a	NM_173018	0.00322313
10341411		---	0.00322589
10473240	Eno1	NM_023119	0.0032262
10415678	Cab39l	NM_026908	0.00322706
10372094		---	0.0032274
10458461	Hdac3	NM_010411	0.0032294
10424363	Nsmce2	NM_026746	0.00323392
10436426	Orai2	NM_178751	0.00323692
10380622	Hoxb9	NM_008270	0.00323782
10422312	Cldn10	NM_023878	0.00323962
10499639	Cks1b	NM_016904	0.00324051
10539433	Mobkl1b	ENSMUST00000(	0.00324141
10605172	Ard1a	NM_019870	0.00324332
10542911	Samd9l	NM_010156	0.00324461
10474793	Pak6	NM_001033254	0.00324644
10340250		---	0.00325069
10338040		---	0.00325323
10561942	Kirrel2	NM_172898	0.0032543
10486026	Zfp770	NM_175466	0.00325593
10544588	Gimap3	NM_031247	0.00325627
10403511	Heatr1	NM_144835	0.00326334
10550102	Lig1	NM_001083188	0.00326362
10344126		---	0.00326397
10542395	Atf7ip	ENSMUST00000(	0.0032673
10552708	Kcnc3	NM_008422	0.00326909
10346348	Spats2l	BC021879	0.00327204
10488589	Fam110a	NM_028666	0.00327464
10488929	Eif6	NM_010579	0.00327473
10475502	Slc28a2	AF079853	0.00327665
10344466		---	0.00327751
10584122	Rpl21	BC094410	0.00327942
10413212	Zmiz1	NM_183208	0.0032908
10487969	Trmt6	NM_175113	0.00329084
10371959	Elk3	NM_013508	0.00329272
10402195	Tc2n	NM_028924	0.0032933
10432972	Rarg	NM_011244	0.00329704
10451195	1600014C23Rik	ENSMUST00000(	0.00329768
10388234	Gsg2	NM_010353	0.00330076
10557405	Rabep2	NM_030566	0.00330552
10490903	Car13	NM_024495	0.00330717
10558150	Htra1	NM_019564	0.00330972
10519691	Gm3360	XR_032742	0.00331098
10496854	Ttll7	NM_027594	0.00331397
10486059		---	0.00331401
10425905	Nup50	NM_016714	0.00331625
10404014	V1ri10	NM_134245	0.00331635
10542596	Slco1c1	NM_021471	0.00331991
10502575	Clca4	NM_139148	0.00332628
10590597	Sacm1l	NM_030692	0.00333042
10397557		---	0.00333082
10375793	Olfr51	NM_146909	0.00333433
10338160		---	0.00333444
10504373	Gm12472	ENSMUST000001	0.00333523
10409709		---	0.0033374
10595622		---	0.00333961
10595636		---	0.00333961
10439411	Nr1i2	NM_010936	0.00334406
10339283		---	0.0033448
10344134		---	0.00334769
10496322	Gm9799	ENSMUST00000(	0.00334806
10561337	1700049G17Rik	NM_028538	0.00334904
10554963	Gm5899	XM_620527	0.00335066
10399696	Rnf144a	NM_001081977	0.00335179
10377725	Dlg4	NM_007864	0.00335276
10533945	Ubc	NM_019639	0.00335636
10369290	Ddit4	NM_029083	0.00335686
10351603	Arhgap30	NM_001005508	0.00335864
10586064	Anp32a	NM_009672	0.00336182
10425421	Fam83f	NM_145986	0.00336204
10455959		---	0.00336368
10481827	Zbtb34	NM_001085507	0.00336632
10473612	Olfr1202	NM_146462	0.00336651
10578277		---	0.00336675
10338969		---	0.00336706
10442147	V1re6	NM_134195	0.00336912
10575095	Has3	NM_008217	0.00337004
10499420	Robld3	NM_031248	0.00337179
10381109	Gm11565	NM_001126323	0.00337375
10557313	4930533L02Rik	ENSMUST00000(	0.00337578
10486681	Tgm5	NM_028799	0.00337596
10438069	Ypel1	NM_023249	0.00337991
10429944	Scrt1	NM_130893	0.00338306
10354404	Dnajb6	NM_001037940	0.00338439
10376897		---	0.00338565
10354203		---	0.0033895
10524227	2410025L10Rik	NM_001142642	0.00339045

10562399	Kctd15	NM_146188	0.00339776
10355668		---	0.00339879
10491014	Hltf	NM_009210	0.00340675
10355931	Farsb	NM_011811	0.00341071
10605566		---	0.00341565
10472197	Rpl29	L08651	0.00341579
10387797	Bcl6b	NM_007528	0.00341594
10603000	Rai2	NM_198409	0.00341811
10371591	4930547N16Rik	NM_029249	0.00342743
10555846		---	0.00342746
10570437	Fbxo25	NM_025785	0.003428
10552258		---	0.00343113
10489004	Nfs1	NM_010911	0.00343258
10526038	Mmp17	NM_011846	0.00343574
10380109	Hsf5	NM_001045527	0.00343577
10352971	Rb1cc1	NM_009826	0.00343766
10503856	Gabrr2	NM_008076	0.00344028
10488642	Defb19	NM_145157	0.00344373
10588893	Rhoa	NM_016802	0.00344649
10489878	Ptgis	NM_008968	0.0034474
10357133		---	0.00344944
10396421	Hif1a	NM_010431	0.00344957
10522749	Lphn3	NM_198702	0.00345623
10367122	Baz2a	NM_054078	0.00345647
10567564	Cdr2	NM_007672	0.00346217
10497944	Mfsd8	NM_028140	0.00346485
10464905	Npas4	NM_153553	0.00346696
10420483	Phf11	NM_172603	0.00346847
10543409	Tas2r118	NM_207022	0.00346888
10417065	Rap2a	NM_029519	0.00347059
10506668	Yipf1	NM_145550	0.00347498
10396795	Eif2s1	NM_026114	0.00347618
10585992	Myo9a	NM_173018	0.00348326
10383502	Slc16a3	NM_030696	0.00348387
10435237	Zfp148	NM_011749	0.00348605
10344127		---	0.00349026
10605455		---	0.00349055
10383756	Ifitm2	NM_030694	0.0034907
10492325	Ube2v1	NM_023230	0.0034912
10541729	Cdca3	NM_013538	0.00349295
10379998	Trim37	NM_197987	0.00349344
10377439	Per1	NM_001159367	0.00349649
10598107	Taf1a	NM_021466	0.00349938
10348739	Sned1	NM_172463	0.00350138
10513112	Epb4.1l4b	NM_019427	0.00350531
10533844	Rilpl2	NM_030259	0.0035084
10518132	Prdm2	NM_001081355	0.00350938
10343036		---	0.00351041
10371877	Slc25a3	NM_133668	0.0035125
10444291	H2-Ab1	NM_207105	0.00351514
10546661	Foxp1	NM_053202	0.00351776
10585706	Cox5a	NM_007747	0.00351934
10497203	Hey1	NM_010423	0.00352077
10445741		---	0.00352086
10568282	Bcl7c	NM_009746	0.00352165
10387525	Mpdu1	NM_011900	0.00352184
10461715	Olfr1441	NM_146683	0.00352187
10386005	Atp5f1	NM_009725	0.0035224
10484756	Olfr1197	NM_001005225	0.003524
10569429	Cdkn1c	NM_001161624	0.00352903
10478718	Ncoa3	NM_008679	0.003531
10421029	Cdca2	NM_175384	0.00353299
10511258	Fam132a	NM_026125	0.00353376
10532741	Tmem119	NM_146162	0.00353507
10554586	4833418N17Rik	AK080712	0.00353527
10583382	Olfr834	NM_001011823	0.00353832
10355511	Tnp1	NM_009407	0.00353859
10520950	Pdlim1	NM_016861	0.0035397
10521690	Ppih	NM_028677	0.00354261
10340497		---	0.00354267
10383214	Rnf213	AK173199	0.0035462
10553092	Dbp	NM_016974	0.00354663
10497321	Pgam1	NM_023418	0.0035509
10503695	Bach2	NM_007521	0.00355455
10581902	Cfdp1	NM_011801	0.00356032
10384622	Ehbp1	NM_153078	0.00356623
10522786		---	0.00356659
10371846	Apaf1	NM_001042558	0.00356705
10428763	Atad2	NM_027435	0.00357101
10487021	Slc30a4	NM_011774	0.00357287
10441394		---	0.00357446
10341998		---	0.00357803
10580169	Ccdc130	NM_026350	0.00358058
10580191	Nfix	NM_001081981	0.00358133
10550400	Pnmal2	NM_001099636	0.00358418
10583386	Olfr835	NM_001012266	0.0035868
10447634	Gm9992	NM_001142539	0.00358861
10379482	Cdk5r1	NM_009871	0.0035888
10544089	Zc3hav1	NM_028421	0.00358902
10489429	Wfdc5	NM_145369	0.00359363
10368654	Nt5dc1	NM_176968	0.00359687
10407892	Cdc2l5	NM_001081058	0.00359864
10491486	Atp11b	NM_029570	0.00359908
10403750	2810021B07Rik	NM_025479	0.00359961
10386789	Ulk2	NM_013881	0.00360036
10463263	Lztfl1	NM_033322	0.00360172
10358563	Hmcn1	NM_001024720	0.00360379
10341046		---	0.0036065
10341813		---	0.00360834
10564888	Unc45a	NM_133952	0.00360906
10490986	4632415L05Rik	BC023403	0.00361054
10455595	Eno1	NM_023119	0.00361155
10431229	Celsr1	NM_009886	0.00361477
10599839	Gm14661	ENSMUST00000(	0.00361664
10572241	Pbx4	NM_001024954	0.00361667
10518113	9030409G11Rik	NM_144531	0.00361716
10596231	Dnajc13	NM_001163026	0.00361948
10341540		---	0.00362486
10498720	Zbbx	NM_172515	0.00362675
10355246	Acadl	NM_007381	0.00362743
10472694	4933404M02Rik	NM_025744	0.00363017
10509023	Syf2	NM_026780	0.0036318
10361104	Ppp2r5a	NM_144880	0.00363784
10570957	Sfrp1	NM_013834	0.00363876
10338209		---	0.00364221
10503188	Chd7	NM_001081417	0.00364529
10471385		---	0.00364713
10363430	Psap	NM_001146120	0.00364743
10578547	Helt	NM_173789	0.00365487
10364468	Bsg	NM_009768	0.00365557
10398297		---	0.00365577
10450868	Olfr98	NM_146510	0.00365898
10392983	Slc25a19	NM_026071	0.00366279
10338051		---	0.00366669
10469559	Msrb2	NM_029619	0.00367206
10565817		---	0.00367279
10473475	Olfr1023	NM_146587	0.00367484
10601854	Wbp5	NM_011712	0.00367689

10523541	Mrps18c	NM_026826	0.00367733
10404402	Foxq1	NM_008239	0.00368705
10350136	Csrp1	NM_007791	0.00369045
10340812		---	0.00369184
10539741	Aak1	NM_001040106	0.00369254
10604599		---	0.00369549
10493076	Sh2d2a	NM_021309	0.00369721
10573112		---	0.00369781
10411532	Mccc2	NM_030026	0.00369822
10562360	Gpi1	NM_008155	0.00371002
10557738	Ctf1	NM_007795	0.00371771
10417273	Esd	NM_016903	0.0037194
10454483	Wdr33	NM_028866	0.00372088
10400357	Baz1a	NM_013815	0.00372101
10603228		---	0.00372159
10475051	Mga	NM_013720	0.00372948
10369586	Supv3l1	NM_181423	0.00373404
10584368	Olfr884	NM_001011798	0.00373837
10469353	Fam23a	NM_001081310	0.00373926
10474006	Phf21a	NM_001109691	0.00374096
10504458	Clta	NM_001080385	0.00374366
10400319	Gm10465	ENSMUST00000(	0.00374891
10568434		---	0.00375056
10535095	Zfand2a	NM_133349	0.00375322
10430593	Josd1	NM_028792	0.00375411
10544501	Ezh2	NM_007971	0.00375714
10467508	Blnk	NM_008528	0.00375775
10589886	4930520O04Rik	ENSMUST00000(	0.00376704
10517383	Nipal3	NM_028995	0.00376922
10548246	Rpl21	BC094410	0.00376994
10385903	Pdlim4	NM_019417	0.00377155
10542691	Lrmp	NM_008511	0.00377318
10458731	Mcc	NM_001085373	0.00377424
10498064	Setd7	NM_080793	0.00377545
10442037	Zfp97	NM_011765	0.00377559
10340478		---	0.00377591
10438109	Ube2l3	NM_009456	0.00378142
10561104	Axl	NM_009465	0.00378554
10554367	Mesp2	NM_008589	0.00378593
10411332	Hmgcr	NM_008255	0.00379133
10452445	4930583I09Rik	ENSMUST00000(	0.0037914
10563718	Gm9717	XM_001474409	0.00379187
10570963	Zmat4	NM_177086	0.00379454
10550601	Snrpd2	NM_026943	0.00379499
10403959	RP23-38E20.1	NM_001097979	0.00379541
10408087	RP23-38E20.1	NM_001097979	0.00379542
10554969	Odz4	NM_011858	0.00379629
10531790	Nkx6-1	NM_144955	0.00379912
10573998	Ogfod1	NM_177767	0.00380044
10592455	Olfr975	NM_146828	0.00380277
10413697	Pbrm1	NM_001081251	0.00380384
10605711	Pdk3	NM_145630	0.00380453
10352798	Kcnh1	NM_010600	0.00380541
10344750	Sgk3	NM_133220	0.00380662
10503194	Chd7	NM_001081417	0.00380662
10574145	Nlrc5	FJ889356	0.00380762
10401365	Zfyve1	NM_183154	0.00381017
10344232		---	0.00381306
10472408	Csrnp3	NM_153409	0.00381534
10420071	Tm9sf1	NM_028780	0.00381666
10571860	Hand2	NM_010402	0.00381913
10349404	Mgat5	NM_145128	0.00382016
10339714		---	0.00382117
10338810		---	0.00382259
10353574	Col19a1	NM_007733	0.00382287
10378754	Fam57a	NM_027773	0.00382458
10378649	Slc43a2	NM_173388	0.00382591
10554249	Acan	NM_007424	0.00382978
10486112	Bmf	NM_138313	0.00383274
10383200		---	0.00383308
10453766	Thoc1	NM_153552	0.00383638
10512774	Coro2a	NM_178893	0.00383777
10416266	Ppp3cc	NM_008915	0.00384222
10366131	1700017N19Rik	NM_001081246	0.00384252
10575034	Cdh3	NM_001037809	0.00384443
10554574	Tm6sf1	NM_145375	0.00384666
10467182	Htr7	NM_008315	0.00384847
10507160	Cyp4a12b	BC060945	0.00385639
10477970	Src	NM_009271	0.00385825
10394862	Ddef2	NM_001135192	0.00385983
10499917	Lce1a2	NM_028625	0.00386027
10594418	Smad6	NM_008542	0.0038649
10455533	Eif1a	NM_010120	0.00386672
10365729	Pctk2	NM_146239	0.00386675
10596904	Ccdc36	NM_001135198	0.00386695
10416371	Lpar6	NM_175116	0.00387652
10388861	Tmem199	NM_199199	0.00387743
10524889	Ksr2	NM_001114545	0.00388101
10598064		---	0.0038821
10345616	Lipt1	NM_001037918	0.0038851
10382846	BC018473	NR_003364	0.00388531
10600545	Hmgb1	NM_010439	0.00388571
10461391	Pcna	NM_011045	0.00388644
10554061	Adamts17	NM_001033877	0.00388902
10378114	Ube2g1	NM_025985	0.00389042
10355806	Tuba4a	NM_009447	0.00389789
10389482		---	0.00389804
10439424	4932425I24Rik	NM_001081025	0.00389817
10498998	D930015E06Rik	BC062940	0.00390074
10408081	Hist1h1b	NM_020034	0.00390204
10457489	6030446N20Rik	BC094382	0.00390349
10339249		---	0.00390437
10514473		---	0.00390604
10607557		---	0.00390724
10434869	Ccdc50	NM_026202	0.00391069
10596747	Sema3f	NM_011349	0.00391074
10502419	Rap1gds1	NM_001040690	0.00391079
10497752	Carhsp1	NM_025821	0.00391255
10511099	A530082C11Rik	NM_177186	0.00391322
10529311	ENSMUSG0000073(	AK144327	0.00391885
10340821		---	0.00391942
10342705		---	0.00392352
10567663	Palb2	NM_001081238	0.00392992
10466288	Olfr1428	NM_146678	0.00393166
10396867	Exd2	NM_133798	0.00393167
10343757		---	0.00393199
10438478	Abcc5	NM_013790	0.00393347
10407509		---	0.00393743
10405870	Vmn2r121	NM_001100616	0.00394277
10388263		---	0.00394501
10608719		---	0.00394859
10598771	Maoa	NM_173740	0.00394941
10460621	1810058N15Rik	ENSMUST00000(	0.00395447
10485607	Qser1	NM_001123327	0.00395663
10398117	Bdkrb2	NM_009747	0.00396669
10343297		---	0.00396723
10580082	C330011M18Rik	ENSMUST00000(	0.00396909
10412665	Rpl21	BC138299	0.00397019

10574139	Nlrc5	FJ889356	0.00397678
10538892	LOC641050	M11024	0.00398034
10338202		---	0.00398041
10566926	Rnf141	NM_025999	0.00398132
10352454		---	0.00398217
10414731	LOC100045878	ENSMUST00000	0.00398323
10496519	Unc5c	NM_009472	0.00398453
10504047	Ube2r2	NM_026275	0.00398729
10471563	C130021I20Rik	ENSMUST00000	0.00399196
10343151		---	0.00399246
10395604	Gm9804	ENSMUST00000	0.00399508
10391625	Ubtf	NM_011551	0.00400086
10575844	Cdh13	NM_019707	0.00400333
10339154		---	0.00400341
10384778		---	0.00400786
10600980	Dgat2l6	NM_001114084	0.00401333
10567086	Cyp2r1	NM_177382	0.00401418
10473024	Agps	NM_172666	0.00401697
10347873	Agfg1	NM_010472	0.00401953
10338150		---	0.00402585
10468180	Psd	NM_028627	0.00402777
10565292	Arnt2	NM_007488	0.00403325
10414493	Olfr748	NM_001011837	0.00403352
10513770		---	0.00403394
10503828	Lyrm2	NM_175364	0.00404326
10379633	Slfn1	NM_011407	0.00404505
10422013	Klf12	NM_010636	0.0040501
10539990	Uroc1	NM_144940	0.00405259
10458873		---	0.00406042
10354111	Aff3	NM_010678	0.00406231
10364841	9030607L17Rik	NM_027829	0.00406283
10379215	Ift20	NM_018854	0.00406508
10471424	Fam102a	NM_153560	0.00406653
10357698	Tmcc2	NM_178874	0.00406726
10466676	1110059E24Rik	BC023385	0.00406774
10596093	Gm5161	XM_356179	0.00406993
10402347	Ifi27l2a	NM_029803	0.00406995
10376956	Hs3st3a1	NM_178870	0.00407552
10499652	4632404H12Rik	ENSMUST00000	0.00407687
10493519	Shc1	NM_001113331	0.00407687
10590791	Birc2	NM_007465	0.0040828
10577412	6820431F20Rik	BC025151	0.00408748
10585986	Myo9a	NM_173018	0.00408794
10375679	Tbc1d9b	NM_029745	0.0040883
10383615	Metrnl	NM_144797	0.00409051
10483624	Dlx1as	NR_002854	0.00409139
10410173	Hiatl1	NM_133680	0.00409432
10604687	Mmgt1	NM_146234	0.00409442
10432243	Fkbp11	NM_024169	0.00409725
10369661	Ccar1	NM_026201	0.0040984
10479159	Zfp831	NM_001099328	0.0041002
10450418	Ly6g6d	NM_033478	0.00410272
10456599	Gm9925	ENSMUST00000	0.0041031
10593384	Dixdc1	NM_178118	0.00410571
10489451	Semg1	NM_017390	0.00410625
10568553	Chst15	NM_029935	0.00410686
10581558	Rps26	NM_013765	0.00410747
10341968		---	0.00411448
10343507		---	0.00411629
10364888	Dot1l	ENSMUST00000	0.00411657
10343365		---	0.00412551
10451860	Pot1b	NM_028370	0.00412719
10380341	Spag9	NM_027569	0.00412822
10341873		---	0.00413061
10416393	Gm6986	XR_034309	0.00413302
10457948	Slc25a46	NM_026165	0.00413348
10430140	Mb	NM_013593	0.00413819
10571321	Ppp1r3b	NM_177741	0.00413849
10477737	Gdf5	NM_008109	0.00414272
10339074		---	0.00414518
10470816	Gle1	NM_028923	0.00414614
10529299	Slbp	NM_009193	0.00415098
10347210	Gm15456	ENSMUST00000	0.00415419
10597960	Slc6a20a	NM_139142	0.00415876
10575861	Hsbp1	NM_024219	0.0041602
10342175		---	0.00416085
10413434	D14Abb1e	NM_001114879	0.00416252
10551791	Zfp420	BC055817	0.00416415
10554723		---	0.00416584
10578193	Eri1	NM_026067	0.00416731
10342411		---	0.004168
10467941	Cwf19l1	NM_001081077	0.00416959
10364417	Olfr8	NM_207201	0.00417158
10380672	Skap1	NM_001033186	0.00417318
10345930	Tpp2	NM_009418	0.00417771
10439471	Ktelc1	NM_172380	0.00417835
10444587		---	0.0041799
10384154	Myo1g	NM_178440	0.00418502
10536635	A430107O13Rik	BC151018	0.00418565
10529082	Mpv17	NM_008622	0.00419051
10398424		---	0.00419527
10486697	Tgm7	NM_001160424	0.00419947
10580282	Junb	NM_008416	0.00420044
10343468		---	0.00420694
10418903	Gprin2	NM_183209	0.00420922
10511069	Gnb1	NM_008142	0.0042099
10557470	Gdpd3	NM_024228	0.0042116
10373498	Rps26	NM_013765	0.00421166
10353341	Hmgb1	NM_010439	0.00421662
10521811		---	0.00421805
10462217	Dmrt1	NM_015826	0.00421829
10544939		---	0.00422014
10412562	Flnb	NM_134080	0.00422196
10342840		---	0.00422259
10601473	Apool	NM_026565	0.00422957
10349316	Tmem185b	NM_146103	0.00423074
10409689	Hnrnpk	NM_025279	0.0042336
10342137		---	0.00423382
10342021		---	0.00423962
10343073		---	0.00423991
10513587	Rnf183	NM_153504	0.00424131
10439881	5330426P16Rik	ENSMUST00000	0.00424285
10559361	Fgf3	NM_008007	0.00424331
10561369	BC089491	NM_175033	0.00424332
10565255	9930013L23Rik	NM_030728	0.00424346
10555007		---	0.00424517
10449596		---	0.00424527
10448748	Nubp2	NM_011956	0.00424683
10560103	Rps8	NM_009098	0.00424956
10495596	Frrs1	NM_001113478	0.0042502
10588243	Ryk	NM_013649	0.00425036
10600765	Pcyt1b	NM_211138	0.00425088
10364824	Csnk1g2	NM_134002	0.00425838
10495405	Slc25a24	NM_172685	0.00426266
10590962	1700012B09Rik	BC092535	0.00426332
10538394	Plekha8	NM_001001335	0.00426412
10489266	Chd6	NM_173368	0.00426419
10425109	Elfn2	NM_183141	0.00426576

10426656	Prph	NM_013639	0.00426691
10481868	Dnajb6	NM_011847	0.00426734
10481114	Abo	NM_030718	0.00427119
10374354		---	0.00427673
10397359	Batf	NM_016767	0.00427887
10536667	Ptprz1	NM_001081306	0.00427971
10441956	Fam120b	NM_024203	0.00428441
10383235	A730011L01Rik	NM_177394	0.00428836
10362245	Epb4.1l2	NM_013511	0.00428998
10423577	Mtdh	NM_026002	0.00429231
10607433	Magea3	NM_020017	0.00429234
10515242	Nsun4	NM_028142	0.00429711
10591706	Elavl3	NM_010487	0.0042985
10506298	Leprot	NM_175036	0.0042988
10380699	Copz2	NM_019877	0.0043011
10608715		---	0.00430426
10449893	A430107D22Rik	NM_178785	0.00430565
10518329	Rpl28	NM_009081	0.00430852
10399290	4930417G10Rik	BC138846	0.0043154
10538590	Herc5	ENSMUST00000(	0.00431551
10427148	Zfp740	NM_153194	0.00431597
10457967	Myo7b	NM_032394	0.00431652
10444717	D17H6S53E	NM_033477	0.0043168
10342472		---	0.00431925
10416242	Pebp4	NM_028560	0.00431931
10475080	Mapkbp1	NM_011941	0.00432339
10469565	Ptf1a	NM_018809	0.00432932
10344321		---	0.00432977
10509620	Capzb	NM_001037761	0.00433384
10522668	Paics	NM_025939	0.00433466
10340849		---	0.00433642
10344244		---	0.00433704
10501744	1700061I17Rik	ENSMUST00000(	0.00433813
10489065	Ndrg3	NM_013865	0.00433904
10481592	Dnm1	NM_010065	0.00434038
10434932	Fam43a	NM_177632	0.00434145
10525961	Piwil1	NM_021311	0.00434206
10392904	Ush1g	NM_176847	0.00434379
10468300	Calhm1	NM_001081271	0.00434503
10397281	Ylpm1	NM_178363	0.00434821
10565422	Tyr	NM_011661	0.00434909
10435271	Heg1	NM_175256	0.00435022
10421970		---	0.00435232
10514388		---	0.004359
10410560	Trip13	NM_027182	0.00435964
10578477	Fam149a	NM_153535	0.00437227
10370334	Lrrc3	NM_145152	0.00437417
10539008	Vps24	NM_025783	0.00437607
10480087	LOC675534	XR_032234	0.00437712
10340007		---	0.00437844
10512024	Mobkl2b	NM_178061	0.00438131
10602975		---	0.00438773
10457203		---	0.00438994
10545720	Stambp	NM_024239	0.00439253
10406466	Tmem161b	NM_175187	0.00439404
10425335	Syngr1	NM_207708	0.00439958
10461594	Ms4a4c	NM_029499	0.0044003
10446771	Lclat1	NM_001081071	0.00440258
10338077		---	0.00440591
10385893	Slc22a4	NM_019687	0.00440772
10598626	Tspan7	NM_019634	0.00440787
10366043	Dusp6	NM_026268	0.00440927
10460466	Ccdc87	NM_207268	0.00441336
10545771	Cyp26b1	NM_175475	0.00441476
10542120	Clec2i	NM_020257	0.00441599
10495964	Neurog2	NM_009718	0.00441881
10412844	Top2b	NM_009409	0.00442435
10527516	Wasf3	NM_145155	0.00442555
10514193		---	0.00442711
10542365		---	0.00442779
10408239	Hist1h3f	NM_013548	0.00442891
10391066	Krt17	NM_010663	0.00443177
10358124	Pkp1	NM_019645	0.00443451
10570236	Mcf2l	NM_178076	0.00443482
10409044	4931429P17Rik	ENSMUST00000(	0.00443484
10388065	Nlrp1b	NM_001162414	0.00443508
10402063	Foxn3	BC029185	0.00443565
10466200	Ms4a7	NM_027836	0.00443821
10568651	Dhx32	NM_133941	0.00443945
10583436	Olfr869	NM_146557	0.00444324
10338581		---	0.00444801
10360334	Olfr1408	NM_146764	0.00445108
10338295		---	0.00445196
10394798	Gm9229	XR_034692	0.00445571
10605429	Gm8666	XR_034103	0.00445682
10420631	Ebpl	NM_026598	0.00445749
10342936		---	0.00445959
10471443	Pip5kl1	NM_198191	0.00446087
10436471	Cggbp1	NM_178647	0.0044645
10422436	Dock9	NM_001081039	0.00446504
10546217	Chchd6	NM_025351	0.0044653
10565689	Capn5	NM_007602	0.00447009
10477551	1700003F12Rik	DQ080431	0.00447086
10603302		---	0.00447273
10563641	Rap1a	NM_145541	0.0044728
10347790		---	0.00447421
10521469		---	0.00447823
10468885	Zfp826	ENSMUST00000(	0.0044822
10343307		---	0.00448321
10553430	Slc6a5	NM_001146013	0.00448617
10565815	Gm4980	ENSMUST00000(	0.0044957
10436253	Senp7	NM_025483	0.00449602
10504755		---	0.00449608
10424064	4930548G14Rik	ENSMUST00000(	0.00449743
10379654	Ap2b1	NM_001035854	0.00449785
10458569	Nr3c1	NM_008173	0.00449894
10434835	Leprel1	NM_173379	0.00449968
10430660	Pdgfb	NM_011057	0.00449969
10346250	Mstn	NM_010834	0.00450635
10467319	Rbp4	NM_001159487	0.00450684
10594246	Gm10655	ENSMUST00000(	0.00452095
10338976		---	0.00452126
10468762	4930506M07Rik	NM_001114312	0.00452273
10357476		---	0.00452681
10588482	Wdr51a	NM_027354	0.00452881
10357381	Ysk4	XM_914055	0.00453216
10479752	Olah	NM_145921	0.00453429
10381860	Mettl2	NM_172567	0.00454046
10607111	Gm4995	XM_908302	0.00454079
10510053	Lrrc38	NM_001162983	0.00454516
10407346		---	0.00454687
10394929	Gm9292	AK132630	0.00454697
10492552	Gm7270	XR_030809	0.00454712
10383767	Osbp2	NM_152818	0.00455527
10578262		---	0.00455974
10379363	Atad5	NM_001029856	0.00456407
10492516	Iqcj	NM_177585	0.00456512
10395259	Nampt	NM_021524	0.00456693

10366350	Krr1	NM_178610	0.00456748
10530283		---	0.00456885
10526564	Ufsp1	NM_027356	0.00457032
10598089		---	0.00457335
10376579	Lrrc48	NM_029044	0.00457961
10487645	Cpxm1	NM_019696	0.00458086
10479869	Cdc123	NM_133837	0.00458421
10521031	Ywhah	NM_011738	0.00458969
10520080	Rint1	NM_177323	0.00459449
10469816	Il1rn	NM_031167	0.00459589
10413282	Fam116a	NM_001134465	0.00459703
10343383		---	0.00459738
10459925	Pqlc1	NM_025861	0.00460119
10497912	Gm5148	NM_198657	0.0046051
10365574	Pmch	NM_029971	0.00460544
10359375	Gpr52	NM_001146330	0.00460951
10593887	Neil1	NM_028347	0.00461228
10491678	Adad1	NM_009350	0.00461275
10404127	Hist1h2aa	NM_175658	0.00461323
10340493		---	0.00461351
10344508		---	0.00461367
10413989	Gm626	XM_985917	0.00461373
10463643		---	0.00461401
10539795	Gkn2	NM_025467	0.00461617
10401511	Tmem90a	NM_001033334	0.00462067
10372230	Myf6	NM_008657	0.0046237
10377782	Mgl1	NM_010796	0.00463401
10339949		---	0.00463472
10504611	E230008N13Rik	ENSMUST00000(	0.00463524
10546884	Lhfpl4	NM_177763	0.00463721
10353288	Ube2w	NM_025773	0.00463884
10386157		---	0.00464234
10392484	Abca8b	NM_013851	0.00464429
10342761		---	0.00464431
10543725	Tsga14	NM_031998	0.00464477
10344173		---	0.00464788
10385500	Irgm1	NM_008326	0.00465064
10491056	Tbl1xr1	NM_030732	0.00465275
10358434	Pla2g4a	NM_008869	0.00465399
10396030	Fancm	NM_178912	0.0046551
10472794	Metapl1	NM_025633	0.00465702
10601705	Cenpi	NM_145924	0.00466271
10533323	Adam1a	NM_172126	0.00466348
10578989	Psd3	NM_177698	0.0046648
10551435	Fcgbp	NM_001122603	0.00466752
10425623	Csdc2	NM_145473	0.00466757
10548614	Tas2r114	NM_207019	0.00467034
10407370	4833420G17Rik	NM_001113550	0.00467073
10555009		---	0.00467344
10478698	Eya2	NM_010165	0.00467855
10420413	Lats2	NM_015771	0.00467886
10399575		---	0.00467926
10414784	Gm26	ENSMUST000001	0.0046919
10414894	Gm26	ENSMUST000001	0.0046919
10380289	Mmd	NM_026178	0.00469757
10476633	Pcsk2	NM_008792	0.0046976
10471770	Olfr357	NM_146623	0.00469879
10340873		---	0.00469935
10458534	Pcdh1	NM_029357	0.00470178
10534654	Znhit1	NM_027318	0.00470515
10415092	4930579G18Rik	ENSMUST00000(	0.00470628
10567645	Ears2	NM_026140	0.00470875
10431546	Tymp	NM_138302	0.00471335
10461553	A430093F15Rik	NR_027805	0.0047141
10378154	Zzef1	NM_001045536	0.00471731
10409915	Cts6	NM_021445	0.00471734
10410929	Agk	NM_023538	0.00471811
10583610	Ilf3	NM_010561	0.00471988
10466404		---	0.00472072
10419850	Cebpe	NM_207131	0.00472765
10369086	Gopc	NM_053187	0.00472801
10373223	Lrp1	NM_008512	0.00472927
10554325	5730590G19Rik	NM_029835	0.00473453
10509253	4930549C01Rik	BC048442	0.00473728
10385507	OTTMUSG00000005	NM_001045540	0.00473839
10395739	Srp54b	NM_001100109	0.00473934
10425814	Mpped1	NM_172610	0.00474174
10394699	Rock2	NM_009072	0.0047427
10507286	Ipp	NM_008389	0.00475203
10414265	BC061237	BC061237	0.00475256
10548810	E330021D16Rik	BC099944	0.00475572
10344346		---	0.00475685
10542857	Far2	NM_178797	0.00475855
10338923		---	0.00476336
10555087		---	0.00476411
10599812	Zic3	NM_009575	0.00476556
10502284	Tet2	NM_001040400	0.00477349
10479402	1600027N09Rik	NM_028479	0.00477813
10495549	Dbt	NM_010022	0.00477911
10348070	Ncl	NM_010880	0.0047798
10368508	2610036L11Rik	NM_001109747	0.00479183
10597969	Gm10052	NR_002885	0.00479193
10405191	Secisbp2	NM_029279	0.00479289
10569994	Hmgb1	NM_010439	0.00479604
10340717		---	0.00479605
10445214	Mut	NM_008650	0.00479738
10451198	Vegfa	NM_001025250	0.00480535
10525179	Oas1d	NM_133893	0.00480975
10566644	Ovch2	NM_172908	0.00481534
10403220	Abcb5	NM_029961	0.00481545
10342263		---	0.00482063
10523376	Fras1	NM_175473	0.00482144
10344717		---	0.00482448
10391755	Ccdc43	NM_025918	0.00482459
10497169	Gm6140	XR_030720	0.00482627
10340118		---	0.00482633
10380944	Zpbp2	NM_027061	0.00483067
10385747	Phf15	NM_199299	0.00483137
10522908	4931407G18Rik	NM_027631	0.00483491
10508800	LOC433762	AY140896	0.00483719
10530652	Scfd2	NM_001114660	0.00484219
10520869	Plb1	NM_001081407	0.00485082
10540118	Lsm3	NM_026309	0.00485237
10585390	Sln	NM_025540	0.00485374
10562670	Zfp715	NM_027264	0.00485778
10360412	Olfr419	NM_146715	0.00485814
10424126	Depdc6	NM_001037937	0.00486274
10413185	Zmiz1	NM_183208	0.00486412
10591608	Dock6	NM_177030	0.00487055
10528457	Orc5l	NM_011959	0.00487396
10435693	Cox17	NM_001017429	0.00487732
10443089	Syngap1	XM_985548	0.00487926
10338018		---	0.00488153
10536118			0.0048839
10432278	Ddn	NM_001013741	0.00488469
10496715	Znhit6	NM_001081094	0.00488584
10376196	Hint1	NM_008248	0.004886
10517090	Arid1a	NM_001080819	0.00489507

10554800	Rab38	NM_028238	0.00489529
10595211	Col12a1	NM_007730	0.00489532
10519490	4921511H03Rik	BC049718	0.00489589
10584067	Zbtb44	NM_001115130	0.00489608
10525365	Hvcn1	NM_001042489	0.00489925
10482712	Stam2	NM_019667	0.00490099
10344950	LOC675046	XM_001473720	0.00490297
10381383		---	0.00490305
10351525	Mpz	NM_008623	0.00490945
10398795	Aspg	NM_001081169	0.00491185
10356267	A530032D15Rik	BC094285	0.0049125
10440206	Arl6	NM_019665	0.00491864
10584288	Robo4	NM_028783	0.0049215
10579703	Cherp	NM_138585	0.00492194
10529567	D5Ertd579e	NM_001081232	0.00493154
10568115	Mvp	NM_080638	0.00493154
10558773	B4galnt4	NM_177897	0.0049321
10607116	Ammecr1	NM_019496	0.00493212
10577312		---	0.00493388
10339559		---	0.00493473
10543067	Asns	NM_012055	0.0049354
10390954	Gm11556	XM_894864	0.00493603
10339640		---	0.00493734
10543772	Klf14	NM_001135093	0.00493917
10395293	Atxn7l1	NM_001033436	0.00494882
10592248	Gm6779	XR_032917	0.00495769
10573152	Ucp1	NM_009463	0.00495831
10597309	Stac	NM_016853	0.00495857
10572975	Ttc29	NM_183096	0.0049593
10514924	Tomm22	NM_172609	0.00496278
10591110	Fat3	NM_001080814	0.00496373
10383192		---	0.00496383
10354845	Orc2l	NM_008765	0.0049661
10445232	3110082D06Rik	BC145294	0.00496873
10416090	Stmn4	NM_019675	0.00497065
10493995	S100a10	NM_009112	0.00497603
10430647		---	0.00498642
10586880	Suhw4	NM_146224	0.00498657
10569057	Rnh1	NM_145135	0.00498832
10496919	Usp33	NM_133247	0.00499046
10555873	Olfr652	NM_147048	0.00499341
10372583	Rab3ip	NM_001003950	0.00499427
10341304		---	0.00499796
10501591	A930005H10Rik	NR_015487	0.00499925
10593508	Ddx10	NM_029936	0.00500755
10505848	Ifnz	NM_197889	0.00500756
10505852	Ifnz	NM_197889	0.00500756
10580061	Il27ra	NM_016671	0.00500792
10342365		---	0.00500884
10340668		---	0.00501141
10604763	Arpc1b	NM_023142	0.00501203
10479112	Gnas	NM_022000	0.00501462
10478938	Hax1	NM_011826	0.00501622
10385870	Irf1	NM_008390	0.00501705
10400089	Gm889	BC147387	0.00501724
10553993	Snrpa1	NM_021336	0.00501744
10445879	Kcnh8	NM_001031811	0.00501909
10504450	Glipr2	NM_027450	0.00501981
10453544	Mettl4	NM_176917	0.00502151
10354803		---	0.00502263
10338008		---	0.00502657
10553646	Herc2	NM_010418	0.00502848
10526098	Scand3	NM_183088	0.00503122
10372177	Tmtc2	NM_177368	0.00503242
10367734	Ust	NM_177387	0.00503425
10403312	Akr1c19	NM_001013785	0.00503483
10473517		---	0.00503572
10511755	Wwp1	NM_177327	0.00503841
10586284	Dpp8	NM_028906	0.0050455
10364051	Snrpd3	NM_026095	0.00504958
10340148		---	0.00505592
10344296		---	0.00505631
10339950		---	0.00505898
10598041		---	0.00506068
10356345	Nppc	NM_010933	0.00506335
10439651	Cd200	NM_010818	0.00506369
10507190	4732418C07Rik	BC059213	0.0050644
10343718		---	0.00506602
10460738	Cdca5	NM_026410	0.00506638
10378059	Txndc17	NM_026559	0.00507331
10554005	H47	NM_024439	0.00507438
10488797	Pxmp4	NM_021534	0.00507497
10338011		---	0.00507645
10340879		---	0.00508112
10584370	Olfr885	NM_001011739	0.00508582
10496822	Gng5	NM_010318	0.005091
10345065	Gsta3	NM_001077353	0.00509207
10433340	4930451G09Rik	ENSMUST00000C	0.00509466
10436828	Olig1	NM_016968	0.00509731
10356968	Pam	NM_013626	0.00509936
10495657	Alg14	ENSMUST00000C	0.00510353
10361139	Traf5	NM_011633	0.00510494
10338597		---	0.00511215
10342770		---	0.00511229
10343540		---	0.00512324
10338061		---	0.00512622
10541301	Tuba8	NM_017379	0.00512704
10446833	Birc6	NM_007566	0.00512804
10401473	Aldh6a1	NM_134042	0.00512916
10349138	Serpinb11	NM_025867	0.00512936
10504905	E130309F12Rik	NM_178756	0.0051372
10415363	Gmpr2	NM_177992	0.00513834
10567316	Tmc7	NM_172476	0.00514114
10407392	BC016423	NM_134063	0.00514125
10511935	Pnrc1	NM_001033225	0.00515009
10344462		---	0.00515312
10562181	Lsr	NM_017405	0.00515488
10484646		---	0.00515588
10385114	Foxi1	NM_023907	0.00515935
10572533	Myo9b	NM_001142322	0.00516039
10462361	Hmgb1	NM_010439	0.00516148
10382152	Helz	NM_198298	0.00516496
10499988	2310007A19Rik	NM_025506	0.00516898
10596652	Hemk1	NM_133984	0.00516924
10373530	Cdk2	NM_183417	0.00517203
10540544	Thumpd3	NM_008188	0.00517358
10437210	Bace2	NM_019517	0.00517622
10441588	Tcp10b	NM_011553	0.00517631
10448441	Amdhd2	NM_172935	0.00518414
10552118	LOC100044517	XM_001472266	0.00519347
10365578	Nup37	NM_028334	0.0051939
10513592	Wdr31	NM_023597	0.00519639
10455738	Snx2	NM_026386	0.00519855
10532330	Ankle2	NM_027922	0.0052001
10352234	Itpkb	NM_001081175	0.00521421
10381738	Wnt3	NM_009521	0.00521513
10526675	Tsc22d4	NM_023910	0.00521555
10424213	Zhx2	NM_199449	0.00521693

10429400		---	0.00522001
10560111	Gm5584	NM_001101534	0.00522041
10352166	9630058J23Rik	BC054802	0.00522226
10395005	Kidins220	NM_001081378	0.00522529
10570344	Lamp1	NM_010684	0.00522622
10516466	Zmym1	NM_026670	0.00523026
10540012	Zxdc	NM_173002	0.00523177
10396108	Arf6	NM_007481	0.00523542
10546086		---	0.00523698
10586458	Csnk1g1	NM_173185	0.00523775
10344425		---	0.00524055
10428809	Klhl38	NM_177755	0.00524257
10341253		---	0.00524384
10534570	Orai2	NM_178751	0.00524424
10350684	Arpc5	NM_026369	0.00524863
10403821	Tcrg-V3	ENSMUST00000	0.005255
10467599	Slit1	NM_015748	0.00525737
10424965	Ppp1r16a	NM_033371	0.0052581
10598586	Xk	NM_023500	0.00526565
10580484	Heatr3	NM_172757	0.00526862
10421810	1190002H23Rik	NM_025427	0.00527314
10413171		---	0.00527946
10501649	Rtcd1	NM_025517	0.00527966
10495332	1700013F07Rik	BC116223	0.00528506
10505858	Ifnz	NM_197889	0.00529061
10516103	Macf1	NM_009600	0.00529266
10399421	Mycn	NM_008709	0.00529309
10343153		---	0.00529506
10557009	Eef2k	NM_007908	0.00529732
10567108	Sox6	NM_011445	0.00529767
10412921	Nid2	NM_008695	0.00530402
10371583	Gm10055	ENSMUST00000	0.00530564
10443830	Cryaa	NM_013501	0.00531112
10605067	Pnck	NM_012040	0.00531829
10402808	Jag2	NM_010588	0.00532078
10601456	Gm6377	NM_001037917	0.00532295
10553897	Mtmr10	NM_172742	0.00532378
10362934		---	0.00532385
10479649	Prpf6	NM_133701	0.00532404
10515744	Cdc20	NM_023223	0.0053295
10606263	Atrx	NM_009530	0.00533598
10365658	Uhrf1bp1l	NM_029166	0.00533647
10442779	Prss29	NM_053260	0.00533784
10340722		---	0.0053394
10521481	Jakmip1	NM_178394	0.00534238
10464363	4930442E04Rik	ENSMUST00000	0.00534298
10505705	Sh3gl2	NM_019535	0.00534319
10549962	Gm5130	XR_033395	0.00535198
10552412	Cldnd2	NM_028849	0.00535362
10602081	Vsig1	NM_030181	0.00536068
10343844		---	0.00536073
10405804	0610007P08Rik	NM_001013608	0.00536325
10497421	Hps3	NM_080634	0.00536387
10495830	Sec24d	NM_027135	0.00536483
10508974	Pafah2	NM_133880	0.00536686
10386058	Sparc	NM_009242	0.00536916
10347117	Cps1	NM_001080809	0.00537687
10566225	Olfr616	NM_147099	0.00537759
10411274	Sv2c	NM_029210	0.0053786
10531952	Abcg3	NM_030239	0.00538133
10459534	Afg3l2	NM_027130	0.00538546
10488860		---	0.00538717
10468527	5830416P10Rik	ENSMUST00000	0.00539753
10490246	3100002L24Rik	ENSMUST00000	0.0053986
10436958	Clic6	NM_172469	0.00540072
10487577	Ckap2l	NM_181589	0.00540643
10341016		---	0.00540889
10541075	Cxcl12	NM_001012477	0.00541143
10398422		---	0.0054131
10426891	Mettl7a1	NM_027334	0.00541567
10398426		---	0.00541929
10515164	Cmpk1	NM_025647	0.0054213
10460693	Frmd8	NM_026169	0.00542276
10523880	Rpap2	NM_144911	0.00542336
10474093	2810002D19Rik	NR_027831	0.00542908
10389775	Pctp	NM_008796	0.00542961
10387170	Ntn1	NM_008744	0.00543456
10338670		---	0.00543862
10570178	A230072I06Rik	ENSMUST00000	0.00543907
10605104	Pdzd4	NM_001029868	0.00543996
10561679	Psmd8	NM_026545	0.00544144
10404566	4933417A18Rik	NM_025750	0.0054461
10465553	Fkbp2	NM_008020	0.00544791
10543319	Fam3c	NM_138587	0.00544803
10449935	Zfp870	NM_207245	0.00544859
10492757	Plrg1	NM_016784	0.00545197
10392347	Pitpnc1	NM_145823	0.00546091
10338588		---	0.00546158
10601086		---	0.00546448
10554162		---	0.00546499
10608716		---	0.00546504
10436519	Robo1	NM_019413	0.00546536
10410364	6720487G11Rik	ENSMUST00000	0.00547104
10359518	Eif4e1b	NM_001033269	0.00547176
10467578	Pik3ap1	NM_031376	0.00547559
10458906	Ppic	NM_008908	0.00548758
10559784	Olfr1347	NM_146385	0.00548838
10546454	Adamts9	NM_175314	0.00549198
10552097	Gm6669	XM_890946	0.0054952
10451884	Rpl21	U93863	0.00549589
10412227	Snx18	NM_130796	0.00549612
10506433	Dab1	NM_177259	0.00550074
10355454	Gm5256	XR_032730	0.0055012
10548829	Gucy2c	NM_001127318	0.00550355
10496539	Gbp5	NM_153564	0.0055087
10442396	Abca3	NM_013855	0.00551477
10344679	St18	NM_173868	0.00551549
10370422	Pwp2	NM_029546	0.00551844
10564375		---	0.00551943
10453082	Hnrpll	NM_144802	0.00552215
10440467	Rpl21	BC094410	0.00553092
10438899	Cpn2	NM_027904	0.00553494
10414175	Gm10713	XM_001474680	0.0055422
10439788		---	0.00554321
10342267		---	0.00554365
10350838	2810417H13Rik	NM_026515	0.0055439
10515481	Dmap1	NM_023178	0.00554421
