# Supplementary material for: miR-182 and miR-10a Are Key Regulators of Treg Specialisation and Stability during Schistosome and Leishmania-associated Inflammation
Source: PLoS Pathog. 2013 Jun 27;9(6):e1003451. doi: 10.1371/journal.ppat.1003451 (PMC3695057; doi:10.1371/journal.ppat.1003451)

Supplementary Table 2

A							
		total	unique	unique	unique	unique	percent unique
sample	sample id	reads	filter	miRNA	genome	total	of total reads
Sm-1	1	12051092	242508	392962	488338	1123808	9.32536238
Sm-2	2	15532258	102801	170083	160818	433702	2.79226626
Sm-3	3	19066403	137009	110736	114473	362218	1.89977103
Sm-4	4	13469369	165857	219945	417708	803510	5.96546134
Lm-1	5	15606322	109158	94163	111199	314520	2.01533712
Lm-2	6	21048407	55239	15743	33249	104231	0.49519662
N1	7	14268959	132450	321171	204430	658051	4.611766
N2	8	16433499	107997	197172	165137	470306	2.86187379
N3	9	21953109	143646	339756	503314	986716	4.49465267
	totals	149429418	1196665	1861731	2198666	5257062	3.51809039

B							
		total	total	total	total	total	percent of all
sample	sample id	reads	filter	miRNA	genome	mapped	reads mapped
Sm-1	1	12051092	4296507	497209	874239	5667955	**47.032709**
Sm-2	2	15532258	7477726	215526	362647	8055899	**51.865601**
Sm-3	3	19066403	9393470	135852	299331	9828653	**51.549592**
Sm-4	4	13469369	4942118	286177	710551	5938846	**44.091494**
Lm-1	5	15606322	7255423	122068	322337	7699828	**49.337877**
Lm-2	6	21048407	11926450	21186	104177	12051813	**57.257601**
N1	7	14268959	4774400	413488	558935	5746823	**40.274998**
N2	8	16433499	6937010	243373	404712	7585095	**46.156299**
N3	9	21953109	11415343	422349	872412	12710104	**57.896601**
	totals	149429418	68418447	2357228	4509341	75285016	**50.381656**

C											miRNA %	5SrRNA %	LSrRNA %	SSrRNA %	tRNA %	
	sample	sample id	total reads	unique mapped	miRNA	5SrRNA	LSrRNA	SSrRNA	tRNA		of uniquely mapped					Other
	Sm-1	1	12051092	1123808	392517	30690	143992	34055	305457		34.927	2.731	12.813	3.03	27.181	
	Sm-2	2	15532258	433702	169779	5119	44164	21620	226896		39.146	1.18	10.183	4.985	52.316	
	Sm-3	3	19066403	362218	110386	10904	55018	21148	164622		30.475	3.01	15.189	5.838	45.448	
	Sm-4	4	13469369	803510	219523	13038	86439	30377	186814		27.321	1.623	10.758	3.781	23.25	
**average**			**15029780.5**	**680809.5**	**223051.25**	**14937.75**	**82403.25**	**26800**	**220947.25**		**32.96725**	**2.136**	**12.23575**	**4.4085**	**37.04875**	**11.20375**
	Lm-1	5	15606322	314520	93750	1761	45086	24692	56519		29.807	0.56	14.335	7.851	17.97	
	Lm-2	6	21048407	104231	15524	1959	8055	3533	90692		14.894	1.879	7.728	3.39	87.011	
**average**			**18327364.5**	**209375.5**	**54637**	**1860**	**26570.5**	**14112.5**	**73605.5**		**22.3505**	**1.2195**	**11.0315**	**5.6205**	**52.4905**	**7.2875**
	N1	7	14268959	658051	320656	7146	58896	30554	150051		48.728	1.086	8.95	4.643	22.802	
	N2	8	16433499	470306	196825	5728	39941	18676	136637		41.85	1.218	8.493	3.971	29.053	
	N3	9	21953109	986716	339233	7626	60180	32664	161140		34.38	0.773	6.099	3.31	16.331	
**average**			**17551855.67**	**705024.3333**	**285571.3333**	**6833.333333**	**53005.66667**	**27298**	**149276**		**41.65266667**	**1.025666667**	**7.847333333**	**3.974666667**	**22.72866667**	**22.771**
		total	149429418	5257062	1858193	83971	541771	217319	1478828							

Example workflow- Sm-1

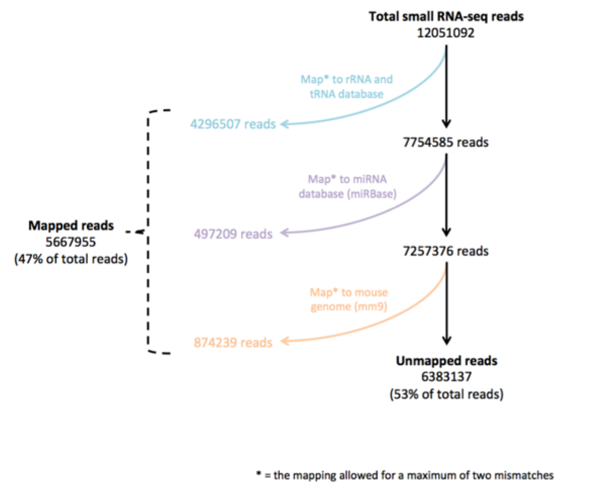

Supplement: Table S2 — Mapping of Deep Sequencing reads. Mapping of data to SOLiD dataset, miRBase and the mouse genome. (A) Uniquely mapped reads, (B) All mapped reads, (C) Representation of other RNA species in dataset. Representative workflow of mapping strategy. Sm = Schistosoma mansoni-derived Treg. Lm = Leishmania major-derived Treg. (PDF) [file ppat.1003451.s010.pdf]
