# Supplementary material for: miR-182 and miR-10a Are Key Regulators of Treg Specialisation and Stability during Schistosome and Leishmania-associated Inflammation
Source: PLoS Pathog. 2013 Jun 27;9(6):e1003451. doi: 10.1371/journal.ppat.1003451 (PMC3695057; doi:10.1371/journal.ppat.1003451)
Supplement: Table S3 — Significantly differentially regulated miRNAs in Foxp3+ populations. Fold change of significantly regulated miRNAs. (PDF) [file ppat.1003451.s011.pdf]

S. mansoni-derived Foxp3$^+$				
**ID given by TargetScanS**	**miRNA**	**Fold-change**	**-log(fold-change)**	**p-value**
miR-210	ID=mmu-mir-210	13.75	3.78	0.0145
miR-183	ID=mmu-mir-183	6.62	2.73	0.0004
miR-182	ID=mmu-mir-182	4.09	2.03	0.0013
miR-125/351	ID=mmu-mir-351	3.71	1.89	0.0003
miR-188	ID=mmu-mir-188	3.52	1.82	0.0422
miR-532	ID=mmu-mir-532	2.83	1.50	0.0014
miR-501	ID=mmu-mir-501	2.67	1.42	0.0122
miR-22	ID=mmu-mir-22	2.41	1.27	0.0351
miR-330	ID=mmu-mir-330	2.12	1.08	0.0111
miR-132/212	ID=mmu-mir-132	2.09	1.07	0.0005
miR-21	ID=mmu-mir-21	2.05	1.04	0.0338
miR-149	ID=mmu-mir-149	1.78	0.83	0.0348
miR-744	ID=mmu-mir-744	1.62	0.70	0.0147
miR-324-5p	ID=mmu-mir-324	1.48	0.57	0.0349
miR-10	ID=mmu-mir-10a	0.62	-0.68	0.0307
miR-192/215	ID=mmu-mir-192	0.59	-0.75	0.016
miR-128	ID=mmu-mir-128-1	0.54	-0.90	0.0212
miR-350	ID=mmu-mir-350	0.48	-1.06	0.0468
miR-339	ID=mmu-mir-339	0.45	-1.16	0.0161
miR-30-5p	ID=mmu-mir-30e	0.42	-1.26	0.0334
miR-203.1	ID=mmu-mir-203	0.38	-1.41	0.0425
miR-455	ID=mmu-mir-455	0.36	-1.46	0.0416
miR-19	ID=mmu-mir-19b-1	0.36	-1.48	0.0015
miR-467	ID=mmu-mir-467e	0.33	-1.62	0.0394
miR-15/16/195/424/497	ID=mmu-mir-15b	0.28	-1.83	0.0425
miR-204/211	ID=mmu-mir-211	0.24	-2.05	0.001
miR-25/32/92/363/367	ID=mmu-mir-32	0.22	-2.20	0.0465
miR-151	ID=mmu-mir-151	0.19	-2.37	0.0077
miR-466	ID=mmu-mir-466d	0.17	-2.59	0.0012
miR-99a	ID=mmu-mir-99a	0.16	-2.62	0.0337
miR-342	ID=mmu-mir-342	0.16	-2.63	0.001

L. major-derived Foxp3$^+$				
**ID given by TargetScanS**	**miRNA**	**Fold-change**	**-log(fold-change)**	**p-value**
miR-99/100	ID=mmu-mir-100	3.52	1.81	0.006
miR-674	ID=mmu-mir-674	0.56	-0.85	0.022
miR-151	ID=mmu-mir-151	0.41	-1.27	0.015
miR-17-5p/20/93.mr/106/519.d	ID=mmu-mir-93	0.37	-1.42	0.004
miR-423	ID=mmu-mir-423	0.36	-1.48	0.029
miR-140	ID=mmu-mir-140	0.34	-1.57	0.034
miR-30-5p	ID=mmu-mir-30e	0.32	-1.64	0.026
let-7/98	ID=mmu-let-7g	0.25	-2.02	0.038
miR-15/16/195/424/497	ID=mmu-mir-15b	0.19	-2.43	0.025
miR-155	ID=mmu-mir-155	0.18	-2.46	0.035
miR-132/212	ID=mmu-mir-132	0.17	-2.56	0.006
miR-805	ID=mmu-mir-805	0.16	-2.65	0.014
miR-466	ID=mmu-mir-466g	0.13	-2.92	0.007
miR-342	ID=mmu-mir-342	0.11	-3.18	0.004
miR-467	ID=mmu-mir-467c	0.11	-3.20	0.027
miR-10	ID=mmu-mir-10a	0.08	-3.65	0.009
miR-25/32/92/363/367	ID=mmu-mir-32	0.07	-3.75	0.029
