# Supplementary material for: miR-182 and miR-10a Are Key Regulators of Treg Specialisation and Stability during Schistosome and Leishmania-associated Inflammation
Source: PLoS Pathog. 2013 Jun 27;9(6):e1003451. doi: 10.1371/journal.ppat.1003451 (PMC3695057; doi:10.1371/journal.ppat.1003451)
Supplement: Table S4 — Candidate master regulatory miRNAs identified from Monte Carlo simulation. (PDF) [file ppat.1003451.s012.pdf]

## *Supplementary Table 4*

**L. major-derived Foxp3+**

| miRNA down-regulated | -LOG(p-value) | Significance threshold | p-value |
|---|---|---|---|
| miR-10 | 2.431798276 | 1.30103 | 0.0037 |
| miR-30-5p | 2.086186148 | 1.30103 | 0.0082 |
| let-7/98 | 1.643974143 | 1.30103 | 0.0227 |
| miR-674 | 1.218244625 | 1.30103 | 0.0605 |
| miR-466 | 0.513569521 | 1.30103 | 0.3065 |
| miR-342 | 0.327902142 | 1.30103 | 0.47 |
| miR-25/32/92/363/367 | 0.218891164 | 1.30103 | 0.6041 |
| miR-155 | 0.171533453 | 1.30103 | 0.6737 |
| miR-15/16/195/424/497 | 0.163422725 | 1.30103 | 0.6864 |
| miR-132/212 | 0.149231273 | 1.30103 | 0.7092 |
| miR-17-5p/20/93.mr/106/519.d | 0.126795691 | 1.30103 | 0.7468 |
| miR-423 | 0 | 1.30103 | 1 |
| miR-805 | 0 | 1.30103 | 1 |
| miR-467 | 0 | 1.30103 | 1 |
| miR-151 | 0 | 1.30103 | 1 |
| miR-140 | 0 | 1.30103 | 1 |

***S. mansoni-derived Foxp3$^+$***

| miRNA up-regulated | -LOG(p-value) | Significance threshold | p-value |
|---|---|---|---|
| miR-182 | 1.43651891 | 1.30103 | 0.0366 |
| miR-21 | 1.2823295 | 1.30103 | 0.0522 |
| miR-132/212 | 1.01772877 | 1.30103 | 0.096 |
| miR-324-5p | 0.77754366 | 1.30103 | 0.1669 |
| miR-183 | 0.77676373 | 1.30103 | 0.1672 |
| miR-22 | 0.46813805 | 1.30103 | 0.3403 |
| miR-330 | 0.32312357 | 1.30103 | 0.4752 |
| miR-125/351 | 0 | 1.30103 | 1 |
| miR-532 | 0 | 1.30103 | 1 |
| miR-501 | 0 | 1.30103 | 1 |
| miR-744 | 0 | 1.30103 | 1 |
| miR-149 | 0 | 1.30103 | 1 |
| miR-188 | 0 | 1.30103 | 1 |

| miRNA down-regulated | -LOG(p-value) | Significance threshold | p-value |
|---|---|---|---|
| miR-192/215 | 0.57642648 | 1.30103 | 0.2652 |
| miR-350 | 0.54836705 | 1.30103 | 0.2829 |
| miR-204/211 | 0.50640255 | 1.30103 | 0.3116 |
| miR-15/16/195/424/497 | 0.33582829 | 1.30103 | 0.4615 |
| miR-203.1 | 0.32294082 | 1.30103 | 0.4754 |
| miR-30-5p | 0.31939257 | 1.30103 | 0.4793 |
| miR-342 | 0.28083451 | 1.30103 | 0.5238 |
| miR-10 | 0.21896306 | 1.30103 | 0.604 |
| miR-19 | 0.18104869 | 1.30103 | 0.6591 |
| miR-128 | 0.17153345 | 1.30103 | 0.6737 |
| miR-466 | 0.14599777 | 1.30103 | 0.7145 |
| miR-25/32/92/363/367 | 0.09366496 | 1.30103 | 0.806 |
| miR-467 | 0.06308432 | 1.30103 | 0.8648 |
| miR-151 | 0 | 1.30103 | 1 |
| miR-339 | 0 | 1.30103 | 1 |
| miR-99a | 0 | 1.30103 | 1 |
| miR-455 | 0 | 1.30103 | 1 |
