# Supplementary material for: miR-182 and miR-10a Are Key Regulators of Treg Specialisation and Stability during Schistosome and Leishmania-associated Inflammation
Source: PLoS Pathog. 2013 Jun 27;9(6):e1003451. doi: 10.1371/journal.ppat.1003451 (PMC3695057; doi:10.1371/journal.ppat.1003451)
Supplement: Table S5 — Predicted mRNA targets of miR-182 and miR-10a identified from Monte Carlo analysis. (PDF) [file ppat.1003451.s013.pdf]

Supplementary Table 5

**Gene (Positionally-conserved in at least one* or two other species)**	***L. major*-derived Foxp3$^+$**	***S. mansoni*-derived Foxp3$^+$**
**Mmu-miR-10a**	– 12.53	– 0.68
**ARRDC3**	1.72	-
**BCL6**	1.79	-
**FBXO30**	1.68	-
**H3F3B**	1.6	-
**NR4A3**	2.27	1.96
**RAP2A**	1.54	-
**SPTY2D1**	1.95	-
**TFAP2A (Tcfap2a)**	1.53	-
**TFRC**	1.71	-
***CLCN5**	1.84	2.22
***FOSL2**	2.77	-
**Mmu-miR-182**	-	4.09
**ARHGEF3**	-	– 1.61
**BACH2**	-	– 1.68
**BCL2**	– 2.13	– 1.61
**CD2AP**	-	– 1.84
**CEBPA**	-	– 1.61
**DSCAM**	-	– 1.70
**HDAC9**	-	– 2.00
**KIF1B**	-	– 1.59
**NRN1**	-	– 2.17
**PGAP1**	-	– 1.71
**QSER1**	-	– 1.94
**RAPGEF4**	– 1.56	– 1.68
***CD96**	– 2.31	– 2.33
***KLF3**	– 2.15	– 1.77
***SGK3**	-	– 2.07
***ST6GAL1**	-	– 1.80
